# Financial incentives for vaccination do not have negative unintended consequences

**DOI:** 10.1038/s41586-022-05512-4

**Published:** 2023-01-11

**Authors:** Florian H. Schneider, Pol Campos-Mercade, Stephan Meier, Devin Pope, Erik Wengström, Armando N. Meier

**Affiliations:** 1 University of Zurich, Zürich, Switzerland; 2 CESifo, Munich, Germany; 3 Lund University, Lund, Sweden; 4 University of Copenhagen, Copenhagen, Denmark; 5 Columbia Business School, New York, NY USA; 6 University of Chicago Booth School of Business, Chicago, IL USA; 7 National Bureau of Economic Research, Cambridge, MA USA; 8 Hanken School of Economics, Helsinki, Finland; 9 Unisanté and Lausanne Center for Health Economics, Behavior, and Policy (LCHE), University of Lausanne, Lausanne, Switzerland; 10 Faculty of Business and Economics, University of Basel, Basel, Switzerland

**Keywords:** Economics, Decision making, Human behaviour, Health policy, Public health

## Abstract

Financial incentives to encourage healthy and prosocial behaviours often trigger initial behavioural change^1–11^, but a large academic literature warns against using them^12–16^. Critics warn that financial incentives can crowd out prosocial motivations and reduce perceived safety and trust, thereby reducing healthy behaviours when no payments are offered and eroding morals more generally^17–24^. Here we report findings from a large-scale, pre-registered study in Sweden that causally measures the unintended consequences of offering financial incentives for taking the first dose of a COVID-19 vaccine. We use a unique combination of random exposure to financial incentives, population-wide administrative vaccination records and rich survey data. We find no negative consequences of financial incentives; we can reject even small negative impacts of offering financial incentives on future vaccination uptake, morals, trust and perceived safety. In a complementary study, we find that informing US residents about the existence of state incentive programmes also has no negative consequences. Our findings inform not only the academic debate on financial incentives for behaviour change but also policy-makers who consider using financial incentives to change behaviour.

## Abstract (web summary)

Findings from large-scale studies in Sweden and the USA indicate that providing financial incentives for vaccination and informing about state incentive programmes do not have any negative unintended consequences.

## Main

Offering financial incentives to encourage healthy and prosocial behaviours often triggers initial behaviour change^1–11^, which is why financial incentives have long been considered by academics and policy-makers. For example, financial incentives have been introduced with the intent to foster blood donations^1^, cancer screening rates^2^, smoking cessation^3,4^ and vaccination uptake^5–9^. However, a large and long-standing literature in the social sciences, philosophy, public health and medicine warns against offering financial incentives because of worries about a wide range of negative unintended consequences that may outweigh any initial behaviour change^12–30^. Such worries have led policy-makers and policy-advisors around the world to recommend against using financial incentives to encourage healthy and prosocial behaviours^1,31,32^.

A first central concern is that financial incentives crowd out prosocial motivations, which could result in less healthy behaviour when no payments are offered and a deterioration of morals and the sense of civic responsibility more generally^12–17,25,26^. Philosopher Michael Sandel^12^, for example, warns that offering financial incentives “erodes people’s sense of obligation” and “diminishes the spirit of altruism”. A second concern is that paying people prompts suspicion, potentially making them more hesitant to engage in certain behaviours when no payments are offered. According to this view, financial incentives signal that engaging in a health behaviour is unpleasant, risky or not as effective in improving health, and decreases trust in healthcare providers^18–22^. Other concerns include that incentives might change people’s values^12^, such as attitudes towards financial incentives, and that incentives could undermine people’s sense of self-determination and make them feel coerced into a certain behaviour^23,24,28,29^.

It is difficult to causally measure the unintended consequences of financial incentives. One key difficulty is finding a situation in which some people were randomly offered payments and others were not. For example, the incentive programmes that many governments introduced to increase COVID-19 vaccination uptake affected everyone at the same time and do not allow for a proper control group^33^. A second key difficulty is that studying the many potential consequences of financial incentives requires access not only to comprehensive data on people’s behaviours but also to data about individuals’ morals, perceptions and feelings, which can only be measured with rich survey data.

Here we report findings from a large-scale, pre-registered study that causally measures the unintended consequences of offering financial incentives to encourage healthy and prosocial behaviour (*n* = 5,019). We overcome the identification and measurement difficulties by using a unique setting that provides random variation in exposure to incentives and by combining population-wide administrative records on health behaviours with detailed survey data. We exploit a randomized controlled trial (RCT) in the context of financial incentives for COVID-19 vaccination (P.C.-M. et al., unpublished, and ref. ^5^). Participants were offered payments of 200 Swedish krona (SEK; about US $24 at the time) for taking a first dose of a COVID-19 vaccine, which increased first-dose uptake by 4 percentage points 30 days after the trial (uptake remained higher even 3 months later). The RCT setting is ideal in that it allows us to compare individuals who were randomly offered financial incentives for vaccination with individuals who were not offered any financial incentives. We combine the RCT data with new Swedish administrative records for second-dose uptake and with rich, individual-level survey data.

We document no negative impacts of offering financial incentives for taking a first dose on the timing or likelihood of participants taking the second or the third dose of a COVID-19 vaccine, for which no financial incentives were offered. We also document no effects on other health behaviours, such as blood donations and flu shots. Notably, we find no negative impacts on morals, sense of civic responsibility, trust in vaccination providers, safety and efficacy perceptions of vaccines, attitudes towards financial incentives, and feelings of self-determination and coercion. We incentivized several of the measures in the survey by implementing the choices of some participants, meaning that some of the survey measures could have real consequences and capture actual behaviour. For all outcomes, we can reject small negative impacts of 0.2 standard deviations or larger (Cohen’s *d*), meaning that we can reject that there were even small negative consequences of offering payments for vaccination.

We complement our evidence from Sweden with evidence on the effects of large-scale incentive programmes implemented by US state governments. In a pre-registered study in the USA (*n* = 3,062), participants randomly assigned to the incentives condition received detailed information about their state’s COVID-19 vaccine incentive programme, whereas participants in the control condition did not receive this information. Because most of the participants were unaware that their state offered incentives for vaccination, this experimental design overcomes the identification problems by creating random variation in perceived exposure to incentives. In line with the evidence from Sweden, we find no negative impacts of being informed about incentive programmes on the willingness of participants to take a further dose, morals, trust in the state government, safety and efficacy perceptions of vaccines, or intentions to donate blood or to receive a flu shot.

The COVID-19 pandemic is the biggest health crisis in recent memory. Without very high vaccination rates, the pandemic is set to have large public health and societal impacts for years to come. With few policy tools left to motivate vaccination^34–36^, governments, private companies and organizations across the globe have considered and introduced payments for vaccination^37^. However, evidence on whether payments for COVID-19 vaccination have negative unintended consequences, as many academics and policy-makers fear they do^17,22,24,32,37–41^, is lacking. We report important first evidence, which is key for policy-making aimed at increasing adherence to vaccination schedules for COVID-19 vaccines, including child vaccination, recurrent booster shots for years to come, as well as for other vaccines^42^.

Our findings are also important because worries about unintended consequences of payments reach well beyond vaccination^43^. Financial incentives intended to motivate healthy and prosocial behaviours have been considered in many contexts, for instance, to motivate blood^1,44,45^ and organ donation^46^, to curtail smoking^3,4,47^, to encourage exercising and healthy eating^10,11,48^, to boost medication adherence^49^, to foster clinical trial participation^50,51^ and to increase uptake of preventive measures, such as cancer screening^2,52^. Our findings and methods inform the large and long-standing academic literature discussing the potential negative consequences of financial incentives for behaviour change more generally.

## Evidence from the Swedish RCT

### Measuring unintended consequences

We use a unique combination of random variation in exposure to financial incentives from a previous RCT with comprehensive administrative and survey data (see ‘Data availability’ in Methods). The previous RCT was conducted from May to July 2021 and enrolled 1,131 participants who were offered SEK 200 (about US $24 at the time) to take the first dose of a COVID-19 vaccine within 30 days, forming the financial incentives condition, and 3,888 participants who were not offered any payment, forming the control condition.

We study the unintended consequences of offering financial incentives by using new administrative data collected by the Public Health Agency of Sweden on second-dose uptake and survey data on morals, safety and efficacy perceptions, feelings of self-determination and coercion, and other health behaviours. The Public Health Agency of Sweden linked the RCT data for all 5,019 participants to the COVID-19 vaccination records in late December 2021. We conducted a first survey with the RCT participants in early January 2022. Because the first survey was carried out before the participants were offered a third dose, we conducted a second survey in June 2022 on third-dose uptake. In total, 3,238 participants (2,706 participants) responded to the first survey (second survey), 726 (606) of the participants in the financial incentives condition and 2,512 (2,100) of the participants in the control condition. In both surveys, survey participation was balanced across both conditions, with no differential attrition based on personality characteristics, vaccination status, vaccine hesitancy or sociodemographics (Supplementary Information section 2.1).

We compare the health behaviours, morals, perceptions and feelings in the financial incentives condition to the control condition. We standardize all outcomes and report all results as pre-registered. All reported results in the text, figures and tables come from ordinary least squares (OLS) regressions with heteroscedasticity-robust standard errors and all *P* values come from two-sided *t*-tests (see Methods for details). The analysis has 80% power to detect even very small effects of −0.12 standard deviations at the 5% level, as stated in our pre-registration plan.

### Results from the Swedish RCT

**Fig. 1:**
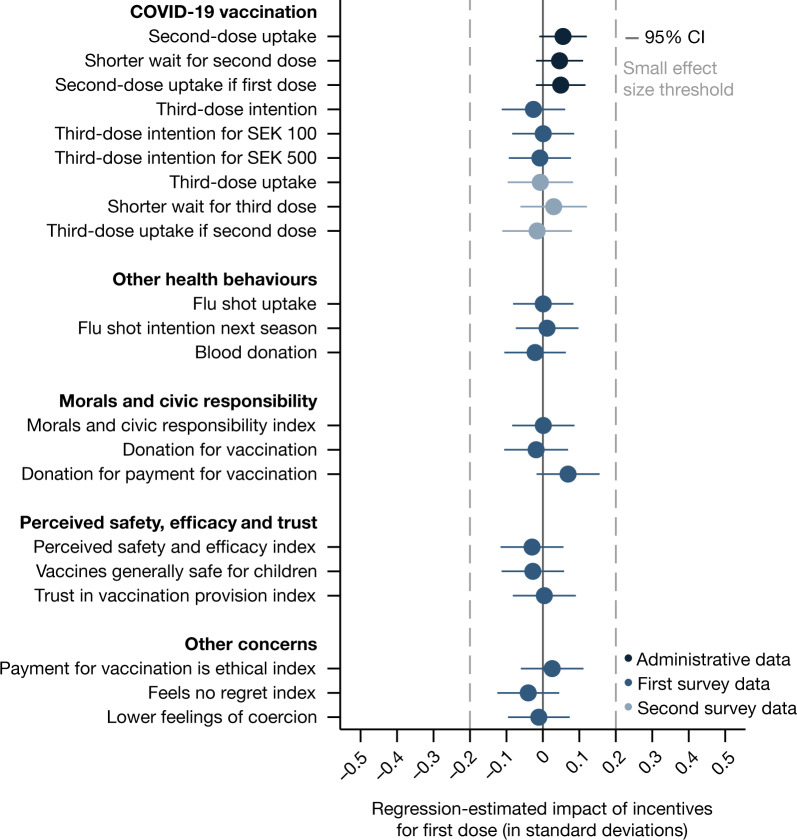
Regression-estimated effects of offering financial incentives for first-dose uptake on further COVID-19 vaccination, other health behaviours, morals and civic responsibility, perceived safety, efficacy and trust, and other concerns. The figure is based on RCT data linked to comprehensive survey data and population-wide Swedish administrative data capturing each vaccination in Sweden. The figure shows regression-estimated effects of the financial incentives condition relative to the control condition. All regressions use the pre-registered controls consisting of gender, age, region, interactions between age and region, being in an at-risk group for COVID-19, civil status, having children in the household, employment status, education, parents’ place of birth and income (see Supplementary Information section 1.1 for details, see Supplementary Information section 2.3 and Extended Data Fig. 1 for results without controls). The blue dots indicate the estimated impact in standard deviations on the respective variables; all outcomes are defined as pre-registered. Error bars represent 95% confidence intervals (two-sided CI: mean ± 1.96 s.e.) from OLS regressions with heteroscedasticity-robust standard errors. The dashed grey lines indicate the threshold for small effect sizes of 0.2 standard deviations (Cohen’s *d*). The sample sizes for the control and incentives conditions across datasets are as follows: administrative data, *n* incentives = 1,132, *n* control = 3,888; first survey data, *n* incentives = 726, *n* control = 2,512; second survey data, *n* incentives = 606, *n* control = 2,100. Source data

We first study the concern that offering financial incentives may reduce future vaccination uptake and other health behaviours when no payments are offered. Using administrative data on vaccination uptake, Fig. 1 and Table 1 show no evidence that participants in the financial incentives condition were less likely to take an unincentivized second dose of a COVID-19 vaccine (if anything, uptake increased; OLS regression,* B* = 0.055, standard error (s.e.) = 0.033,* P* = 0.097) or to delay the uptake of the second dose (OLS regression, *B* = 0.046, s.e. = 0.033,* P* = 0.164). We also do not find any effects on second-dose uptake when we restrict the sample to those who took the first dose (OLS regression, *B* = 0.049, s.e. = 0.035,* P* = 0.158). Using data from the first survey, we do not find evidence that offering monetary incentives affected the intention to take the third dose (OLS regression, *B* = −0.026, s.e. = 0.044,* P* = 0.560) nor the willingness of participants to take a third dose if they were hypothetically offered SEK 100 (OLS regression, *B* = 0.001, s.e. = 0.043,* P* = 0.983) or SEK 500 (OLS regression, *B* = −0.008, s.e. = 0.043,* P* = 0.850). Using data from the second survey, we do not find negative effects on self-reported actual third-dose uptake (OLS regression, *B* = −0.007, s.e. = 0.046,* P* = 0.879), the delay to take the third dose (OLS regression, *B* = 0.030, s.e. = 0.046,* P* = 0.524) or third-dose uptake when we restrict the sample to those who took the second dose (OLS regression, *B* = −0.016, s.e. = 0.049,* P* = 0.745).

**Table 1:** Regression-estimated treatment effects of offering financial incentives for first-dose uptake, corresponding *P* values, 95% confidence intervals and equivalence tests against an effect more negative than −0.2 standard deviations

Dependent variable	Treatment effect (standard deviations)	Standard error	*P* value (two-sided *t*-test)	95% confidence interval	Equivalence testing *P* value
**COVID-19 vaccination:**					
Second-dose uptake	0.055	0.033	0.097	[−0.010, 0.120]	<0.0001
Shorter wait for second dose	0.046	0.033	0.164	[−0.019, 0.110]	<0.0001
Second-dose uptake if first dose	0.049	0.035	0.158	[−0.019, 0.117]	<0.0001
Third-dose intention	−0.026	0.044	0.560	[−0.113, 0.061]	<0.0001
Third-dose intention for SEK 100	0.001	0.043	0.983	[−0.084, 0.086]	<0.0001
Third-dose intention for SEK 500	−0.008	0.043	0.850	[−0.093, 0.077]	<0.0001
Third-dose uptake	−0.007	0.046	0.879	[−0.097, 0.083]	<0.0001
Shorter wait for third dose	0.030	0.046	0.524	[−0.061, 0.121]	<0.0001
Third-dose uptake if second dose	−0.016	0.049	0.745	[−0.111, 0.080]	0.0001
**Other health behaviours:**					
Flu shot uptake	0.001	0.042	0.982	[−0.082, 0.084]	<0.0001
Flu shot intention next season	0.012	0.044	0.792	[−0.074, 0.097]	<0.0001
Blood donation	−0.021	0.043	0.619	[−0.106, 0.063]	<0.0001
**Morals and civic responsibility:**					
Morals and civic responsibility index	0.001	0.043	0.979	[−0.084, 0.086]	<0.0001
Donation for vaccination	−0.018	0.045	0.679	[−0.106, 0.069]	<0.0001
Donation for payment for vaccination	0.069	0.044	0.117	[−0.017, 0.155]	<0.0001
**Perceived safety and efficacy:**					
Perceived safety and efficacy index	−0.030	0.044	0.499	[−0.116, 0.057]	0.0001
Vaccines generally safe for children	−0.028	0.044	0.528	[−0.113, 0.058]	<0.0001
Trust in vaccination provision index	0.004	0.044	0.928	[−0.082, 0.090]	<0.0001
**Other concerns:**					
Payment for vaccination is ethical index	0.025	0.044	0.560	[−0.060, 0.111]	<0.0001
Feels no regret index	−0.040	0.043	0.352	[−0.125, 0.045]	0.0001
Lower feelings of coercion	−0.011	0.043	0.797	[−0.096, 0.074]	<0.0001

**Extended Data Fig. 1:**
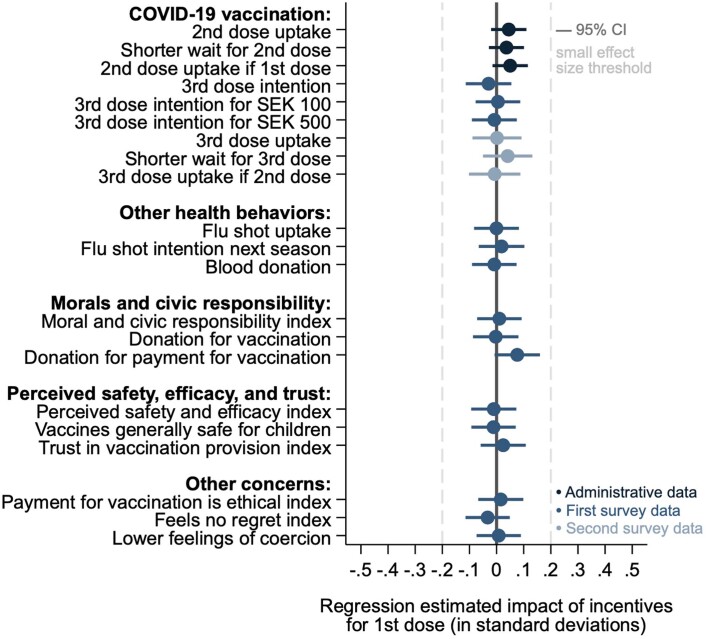
Raw treatment effects of offering financial incentives for first-dose uptake on further COVID-19 vaccination, other health behaviours, morals and civic responsibility, perceived safety, efficacy and trust, and other concerns. The figure is based on RCT data linked to comprehensive survey data and population-wide Swedish administrative data capturing each vaccination in Sweden. The figure shows the raw treatment effects (no control variables included) of the financial incentives condition relative to the control condition. The blue dots indicate the estimated impact in standard deviations on the respective variables; all outcomes are defined exactly as pre-registered. Error bars represent 95% confidence intervals (two-sided CI: mean ± 1.96 s.e.) from OLS regressions with heteroscedasticity-robust standard errors. The dashed grey lines indicate the threshold for small effect sizes of 0.2 standard deviations (Cohen’s *d*). The sample sizes for the control and incentives conditions across datasets are as follows: administrative data, *n* incentives = 1,132, *n* control = 3,888; first survey data, *n* incentives = 726, *n* control = 2,512; second survey data, *n* incentives = 606, *n* control = 2,100.

The table is based on RCT data linked to population-wide Swedish administrative data (second-dose uptake, shorter wait for second dose, second-dose uptake if first dose) and comprehensive data from two surveys (all other outcomes). The table shows coefficient estimates from linear regressions of each standardized outcome on an indicator for the financial incentives condition. Heteroscedasticity-robust standard errors and corresponding *P* values based on two-sided *t*-tests (without multiple comparison adjustments) are also shown. All regressions use the pre-registered controls consisting of gender, age, region, interactions between age and region, being in an at-risk group for COVID-19, civil status, having children in the household, employment status, education, parents’ place of birth and income (see Supplementary Information section 1.1 for details, see Supplementary Information section 2.3 and Extended Data Fig. 1 for results without controls). Equivalence testing corresponds to a one-sided *t*-test of the null hypothesis that the estimated effect is more negative than −0.2 standard deviations (see Supplementary Information section 2.2.4 for details). The sample sizes for the control and incentives conditions across datasets are as follows: administrative data, *n* incentives = 1,132, *n* control = 3,888; first survey data, *n* incentives = 726, *n* control = 2,512; second survey data, *n* incentives = 606, *n* control = 2,100.

In line with these results, we find no evidence that incentives affected other health behaviours, such as flu shot uptake (OLS regression, *B* = 0.001, s.e. = 0.042,* P* = 0.982) and blood donations (OLS regression, *B* = −0.021, s.e. = 0.043,* P* = 0.619) in the previous 5 months. To summarize, we do not find that financial incentives reduced health behaviours when no payments were offered. However, these results do not address the concerns that incentives affect people more broadly, by eroding morals, decreasing safety and efficacy perceptions, and affecting feelings of self-determination and coercion.

Next, we study the concern that financial incentives could erode participants’ morals and civic responsibility by using a combination of survey questions and behavioural data collected in the first survey. Figure 1 and Table 1 show no evidence that incentives affected our pre-registered index of morals and civic responsibility (OLS regression, *B* = 0.001, s.e. = 0.043, *P* = 0.979), which consists of three questions measuring participants’ sense of moral obligation to receive a COVID-19 vaccine for the good of society. We also measure altruism in the context of vaccination by offering participants the possibility to donate money to two non-governmental organizations (NGOs) that promote vaccinations. The first NGO provides COVID-19 vaccines in areas with limited access to vaccination and the second attempts to increase vaccination uptake by offering financial incentives. We do not find any differences in the amount given between the financial incentives condition and the control condition (OLS regressions, *B* = −0.018, s.e. = 0.045,* P* = 0.679 and *B* = 0.069, s.e. = 0.044,* P* = 0.117, respectively).

Third, we study the concern that offering financial incentives signals that vaccines are not safe and effective, which could ultimately decrease trust in vaccination providers. Figure 1 and Table 1 show no evidence that incentives affected our pre-registered index of safety and efficacy of COVID-19 vaccines (OLS regression, *B* = −0.030, s.e. = 0.044,* P* = 0.499), which includes three questions on safety perceptions, vaccine efficacy beliefs and whether participants are worried about the side-effects of COVID-19 vaccines. We also find no evidence that offering incentives affected the belief that vaccines in general are safe for children (OLS regression, *B* = −0.028, s.e. = 0.044,* P* = 0.528), nor an index capturing people’s trust in researchers, the public health agency and pharmaceutical companies concerning the provision of COVID-19 vaccines (OLS regression, *B* = 0.004, s.e. = 0.044,* P* = 0.928).

Further, we study the concerns that incentives could affect participants’ feelings of self-determination and coercion about their decision on whether to receive the first dose of a COVID-19 vaccine. Figure 1 and Table 1 show no evidence that participants who were offered incentives to take the first dose were more likely to say that they felt forced to take it (OLS regression, *B* = −0.011, s.e. = 0.043,* P* = 0.797) or more likely to regret their decision on whether to take the first dose (OLS regression, *B* = −0.040, s.e. = 0.043,* P* = 0.352). Finally, we also do not find any impacts on people’s political views about whether paying people for vaccination is ethically acceptable (OLS regression, *B* = 0.025, s.e. = 0.044, *P* = 0.560).

**Fig. 2:**
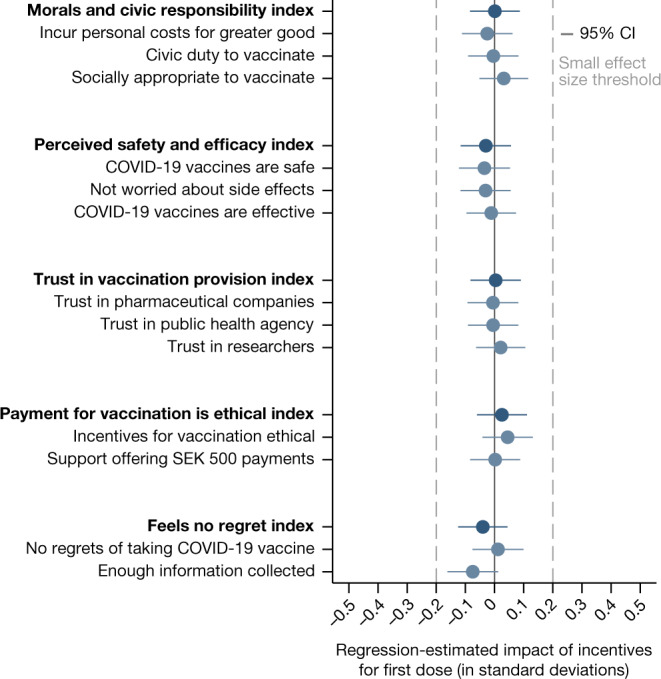
Regression-estimated effects of offering financial incentives for first-dose uptake on single items of indices. The figure is based on RCT data linked to comprehensive survey data. The figure shows regression-estimated effects of the financial incentives condition relative to the control condition on the single items of all indices. All regressions use the pre-registered controls described in Fig. 1 and Supplementary Information section 1.1. The blue dots indicate the estimated impact in standard deviations on the respective variables. Error bars represent 95% confidence intervals (two-sided CI: mean ± 1.96 s.e.) from OLS regressions with heteroscedasticity-robust standard errors. The dashed grey lines indicate the threshold for small effect sizes of 0.2 standard deviations (Cohen’s *d*). The sample sizes for the control and incentives conditions are *n* incentives = 726 and *n* control = 2,512. Source data

Out of 21 coefficient estimates reported in Fig. 1 and Table 1, none of the 95% confidence intervals crosses the Cohen’s *d* small effect size threshold of an effect of 0.2 standard deviations. These results are robust to a battery of robustness checks, such as using each of the items underlying the pre-registered indices separately, as shown in Fig. 2, including different sets of control variables than those we pre-registered (Extended Data Fig. 1 and Supplementary Information section 2.3), and considering secondary outcome variables (Supplementary Information section 2.4).

Overall, Fig. 1 and Table 1 do not show any discernible negative impacts of financial incentives across the distribution of coefficient estimates. The mean coefficient is 0.004, the median coefficient is 0.001, the largest negative coefficient is −0.040 and the largest positive coefficient is 0.069, confirming the visual impression of only very small, if any, impacts. In addition, we test whether the outcomes have a different dispersion in the financial incentives condition than in the control condition and find that outcomes are not only essentially equal in means but also in distribution (for regression results and raw distributions, see Supplementary Information section 2.4.1, Supplementary Figs. S12–S15 and section 2.4.2).

Equivalence testing further demonstrates that there were no meaningful negative impacts across all outcomes. For the equivalence testing^53,54^, we use the standard effect size threshold for small effects of 0.2 standard deviations as the smallest effect size of interest. Table 1 shows that tests for all outcomes are highly statistically significant, clearly rejecting negative impacts more negative than −0.2 standard deviations (largest *P* = 0.0001). The test results are similar when we specify the smallest effect size of interest as the smallest effect size that our study design can reliably detect based on the pre-registration^54^ (see the Supplementary Information section 2.2.4 for details). Overall, the results provide strong evidence against even small negative consequences of offering payments for vaccination.

### No impacts on different groups

We further explore whether any of the treatment effects differ based on variables measured before offering incentives, including vaccine hesitancy, as well as sociodemographics such as income, education, age and gender. The theoretical concerns in the literature on unintended consequences concern mainly individuals with positive vaccination attitudes; these are the individuals whose prosocial and intrinsic motivation could be crowded out and who might start doubting the safety and efficacy of vaccines. On the other hand, offering incentives might make the hesitant even more sceptical of vaccination.

The data do not indicate consistent negative effects for either relatively vaccine positive or hesitant groups. A potential limitation of these findings is that we do not study a very hesitant population^55,56^. In addition, we find similarly muted treatment effects across sociodemographic subgroups. Overall, we do not find that any of the groups suffered from negative unintended consequences (Extended Data Tables 1–4; for further details and regression results, see Supplementary Information section 2.4.3).

### Different entities offering incentives

An open question is whether the impact of incentives differs when paid by public institutions rather than researchers. We examine this question in a complementary study in Sweden (*n* = 1,001). We use the fact that the previous RCT was implemented in a collaboration between researchers and the Public Health Agency of Sweden. Some study participants were informed that “a team of researchers participated in the implementation of the incentive programme”, whereas others were told that the Public Health Agency of Sweden did so (see Supplementary Information section 1.1.6 for details).

**Fig. 3:**
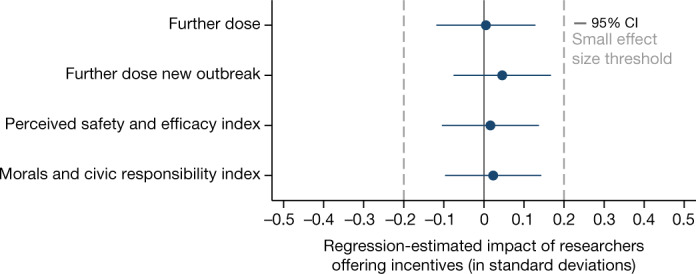
Regression-estimated effects of informing Swedish residents about researchers versus the public health authorities being involved in offering vaccination incentives on further COVID-19 vaccination, morals and civic responsibility, and perceived safety and efficacy. The figure is based on experimental data from a general population sample of Swedish residents. The figure shows regression-estimated effects of the researcher condition (informing participants that researchers participated in the implementation of an incentive programme) relative to the government condition (informing participants that the Public Health Agency of Sweden participated in the implementation of an incentive programme), as pre-registered. All regressions use controls consisting of gender, age, education and income (see Supplementary Information section 2.5 for results without controls). The blue dots indicate the estimated impact in standard deviations on the respective variables; all outcomes are defined as pre-registered. Error bars represent 95% confidence intervals (two-sided CI: mean ± 1.96 s.e.) from OLS regressions with heteroscedasticity-robust standard errors. The dashed grey lines indicate the threshold for small effect sizes of 0.2 standard deviations (Cohen’s *d*). The sample sizes for the control and incentives conditions are *n* researcher = 515 and *n* government = 486. Source data

As shown in Fig. 3, we find no evidence that people’s reactions to financial incentives depended on whether they were informed that the public health authorities or researchers offered the payments. Equivalence testing further shows that we can clearly reject even small negative treatment effects of 0.2 standard deviations (Supplementary Information section 2.5).

## Evidence from US incentive programmes

### Informing people about incentive programmes

In 2021, many US states introduced financial incentives, ranging from small, guaranteed rewards to lotteries that gave vaccinated individuals a chance to win large prizes^57^. Much of the debate in the USA about unintended consequences of financial incentives has focused on these state incentive programmes^22,24,37,41^. In this section, we complement the evidence from Sweden by studying whether learning about the existence of US state incentive programmes had unintended consequences.

Worries about the unintended consequences of monetary incentives apply to this setting as well. Learning about the existence of a state incentive programme could, for example, signal to participants that selfishness is an appropriate response—thereby eroding morals—or that being vaccinated is risky. This could in turn reduce future vaccination uptake. Moreover, although there is debate about the success of some of these state incentive programmes, with mixed empirical evidence^57–62^, unintended consequences can occur in either case (see the discussion in Supplementary Information section 2.6.9). For instance, individuals may not be more likely to vaccinate in response to incentives but may grow more suspicious of vaccinations or change their vaccination morals.

We conducted a pre-registered experiment in June and July 2022 using a general population sample from 12 states that implemented vaccine incentive programmes (*n* = 3,062). We use the fact that many people in the USA (62.3% in our sample) were unaware that state governments rolled out financial incentive programmes, as states often did not publicize the programmes aggressively^58^.

We randomly allocated participants to two treatment conditions, the incentives condition and the control condition (see Methods for details). The participants randomly assigned to the incentives condition received detailed information about their state’s COVID-19 vaccine incentive programme, whereas participants in the control condition did not receive this information. For example, treated participants who resided in California in 2021 were told that the government of California implemented the ‘Vax for the Win’ programme, which distributed more than $100 million in cash prizes and $50 gift or grocery cards from May 2021 to January 2022. Such provision of information creates random variation in perceived exposure to incentives.

**Extended Data Fig. 2:**
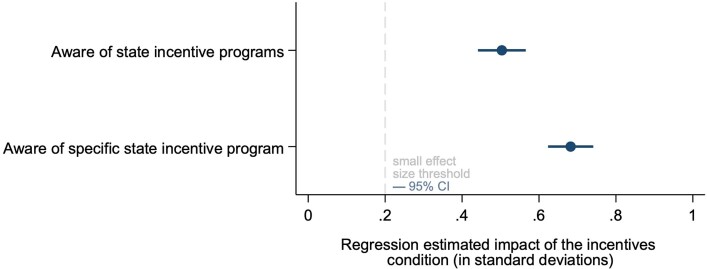
Regression-estimated effects of informing US residents about state vaccination incentive programmes on awareness. The figure is based on experimental data from a general population sample of US residents in 12 states that offered incentive programmes for COVID-19 vaccination. The figure shows regression-estimated effects of the incentives condition (informing participants about the existence of incentive programmes in their state) relative to the control condition. The blue dots indicate the estimated impact in standard deviations on the general awareness about state incentive programmes and the awareness of the specific state incentive programme the participants were informed about. Error bars represent 95% confidence intervals (two-sided CI: mean ± 1.96 s.e.) from OLS regressions with heteroscedasticity-robust standard errors. The dashed grey line indicates the threshold for small effect sizes of 0.2 standard deviations (Cohen’s *d*). The sample sizes for the control and incentives conditions are *n* incentives = 1,521 and *n* control = 1,541.

We analysed whether the provision of information had any impact on the willingness of participants to receive future shots of a COVID-19 vaccine, morals and safety perceptions. To avoid experimenter demand effects, we measured these outcomes in an apparently unrelated follow-up survey about 5 days after the survey in which we provided the information (Methods). The data from the follow-up survey shows that participants who received information about the state incentive programmes were still aware of them 5 days later (Extended Data Fig. 2 and Supplementary Information section 2.6.3). All reported results in the text, figures and tables come from OLS regressions with heteroscedasticity-robust standard errors and all *P* values come from two-sided *t*-tests (see Methods for details).

### Results from the US study

**Fig. 4:**
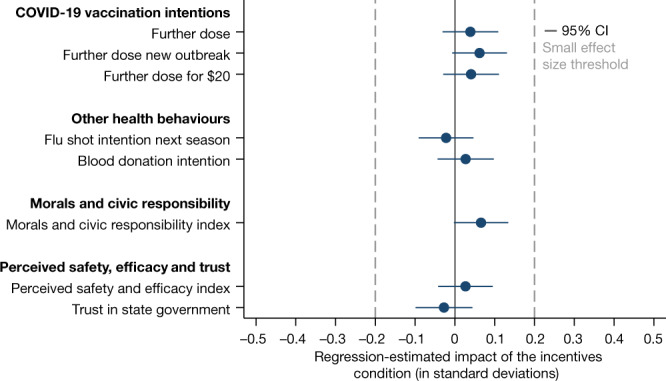
Regression-estimated effects of informing US residents about state vaccination incentive programmes on further COVID-19 vaccination, other health behaviours, morals and civic responsibility, and perceived safety, efficacy and trust. The figure is based on experimental data from a general population sample of US residents in 12 states that introduced incentive programmes for COVID-19 vaccination. The figure shows regression-estimated effects of the incentives condition (informing participants about the existence of incentive programmes in their state) relative to the control condition, as pre-registered. All regressions use controls consisting of gender, age, education, employment status, income and state of residence in 2021 (see Extended Data Fig. 3 for results without controls). The blue dots indicate the estimated impact in standard deviations on the respective variables; all outcomes are defined as pre-registered. Error bars represent 95% confidence intervals (two-sided CI: mean ± 1.96 s.e.) from OLS regressions with heteroscedasticity-robust standard errors. The dashed grey lines indicate the threshold for small effect sizes of 0.2 standard deviations (Cohen’s *d*). The sample sizes for the control and incentives conditions are *n* incentives = 1,521 and *n* control = 1,541. Source data

Figure 4 and Table 2 show no evidence that participants in the incentives condition who were informed about the existence of US state incentive programmes were less willing to receive a further dose within the next 6 months (OLS regression, *B* = 0.039, s.e. = 0.036,* P* = 0.276), to receive a further dose in case there would be a new outbreak (if anything, their willingness increased; OLS regression, *B* = 0.062, s.e. = 0.035,* P* = 0.077) or to receive a further dose if their state government offered them $20 for it (OLS regression, *B* = 0.041, s.e. = 0.036,* P* = 0.256). We also do not find that participants in the incentives condition were less willing to take a flu shot next winter (OLS regression, *B* = −0.022, s.e. = 0.035,* P* = 0.528) or to donate blood (OLS regression, *B* = 0.027, s.e. = 0.036,* P* = 0.454). Finally, we find no evidence that incentives eroded participants’ morals and civic responsibility (if anything, morals improved; OLS regression, *B* = 0.066, s.e. = 0.035,* P* = 0.060), their safety and efficacy perceptions about the COVID-19 vaccines (OLS regression, *B* = 0.027, s.e. = 0.035,* P* = 0.448) or their trust in the state government (OLS regression, *B* = −0.027, s.e. = 0.036,* P* = 0.450).

**Table 2:** Regression-estimated effects of informing US residents about state vaccination incentive programmes, corresponding *P* values, 95% confidence intervals and equivalence tests against an effect more negative than −0.2 standard deviations

Dependent variable	Treatment effect (standard deviations)	Standard error	*P* value (two-sided *t*-test)	95% confidence interval	Equivalence testing *P* value
**COVID-19 vaccination intentions:**					
Further dose	0.039	0.036	0.276	[−0.031, 0.109]	<0.0001
Further dose new outbreak	0.062	0.035	0.077	[−0.007, 0.130]	<0.0001
Further dose for $20	0.041	0.036	0.256	[−0.030, 0.111]	<0.0001
**Other health behaviours:**					
Flu shot intention next season	−0.022	0.035	0.528	[−0.091, 0.047]	<0.0001
Blood donation intention	0.027	0.036	0.454	[−0.044, 0.097]	<0.0001
**Morals and civic responsibility:**					
Morals and civic responsibility index	0.066	0.035	0.060	[−0.003, 0.134]	<0.0001
**Perceived safety, efficacy and trust:**					
Perceived safety and efficacy index	0.027	0.035	0.448	[−0.042, 0.095]	<0.0001
Trust in state government	−0.027	0.036	0.450	[−0.099, 0.044]	<0.0001

**Extended Data Fig. 3:**
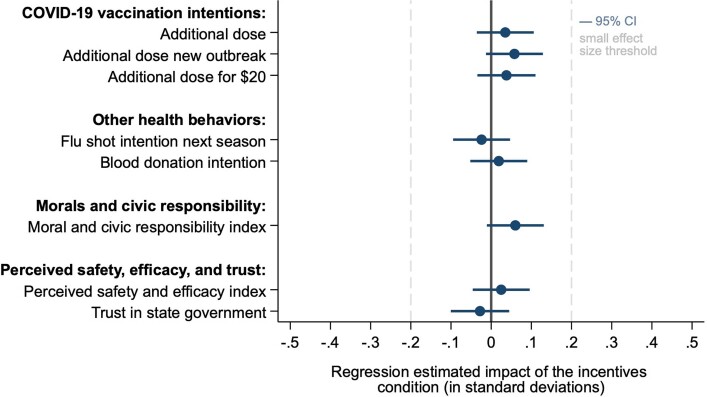
Raw treatment effects of informing US residents about state vaccination incentive programmes on further COVID-19 vaccination, other health behaviours, morals and civic responsibility, and perceived safety, efficacy and trust. The figure is based on experimental data from a general population sample of US residents in 12 states that offered incentive programmes for COVID-19 vaccination. The figure shows the raw effects (no control variables included) of the incentives condition (informing participants about the existence of incentive programmes in their state) relative to the control condition. The blue dots indicate the estimated impact in standard deviations on the general awareness that the state had an incentive programme in place and on the awareness about the specific state incentive programmes participants were informed about. Error bars represent 95% confidence intervals (two-sided CI: mean ± 1.96 s.e.) from OLS regressions with heteroscedasticity-robust standard errors. The dashed grey lines indicate the threshold for small effect sizes of 0.2 standard deviations (Cohen’s *d*). The sample sizes for the control and incentives conditions are *n* incentives = 1,521 and *n* control = 1,541.

The table is based on experimental data from a general population sample of US residents in 12 states that offered incentive programmes for COVID-19 vaccination. The table shows coefficient estimates from linear regressions of each standardized outcome on an indicator for the incentives condition (informing participants about the existence of incentive programmes in their state). Heteroscedasticity-robust standard errors and corresponding *P* values based on two-sided *t*-tests (without multiple comparison adjustments) are also shown. All regressions use controls consisting of gender, age, education, employment status, income and state of residence in 2021 (see Extended Data Fig. 3 for results without controls). Equivalence testing corresponds to a one-sided *t*-test of the null hypothesis that the estimated effect is more negative than −0.2 standard deviations (see Supplementary Information section 2.6.5 for details). The sample sizes for the control and incentives conditions are *n* incentives = 1,521 and *n* control = 1,541.

**Extended Data Fig. 4:**
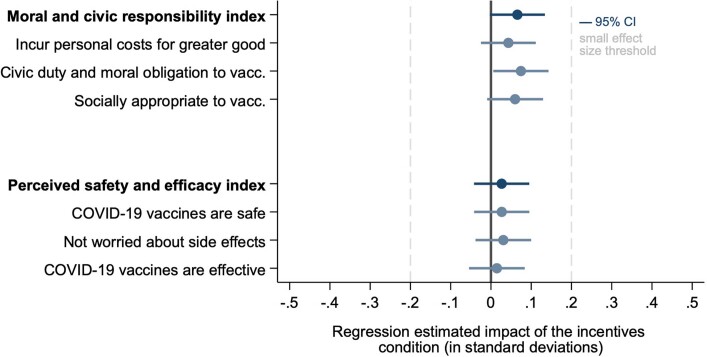
Regression-estimated effects of informing US residents about state vaccination incentive programmes on single items of indices. The figure is based on experimental data from a general population sample of US residents in 12 states that offered incentive programmes for COVID-19 vaccination. The figure shows regression-estimated effects of the incentives condition (informing participants about the existence of incentive programmes in their state) relative to the control condition. The blue dots indicate the estimated impact in standard deviations on the respective variables. Error bars represent 95% confidence intervals (two-sided CI: mean ± 1.96 s.e.) from OLS regressions with heteroscedasticity-robust standard errors. The dashed grey lines indicate the threshold for small effect sizes of 0.2 standard deviations (Cohen’s *d*). The sample sizes for the control and incentives conditions are *n* incentives = 1,521 and *n* control = 1,541.

Overall, Fig. 4 and Table 2 do not show any discernible negative impacts of receiving information about the existence of US state incentive programmes. In Supplementary Information section 2.6, we show that our results are robust to a battery of robustness checks, such as including different sets of control variables (Extended Data Fig. 3), using each of the items underlying the pre-registered indices separately (Extended Data Fig. 4) and using different inclusion criteria.

Out of all coefficient estimates reported in Fig. 4 and Table 2, none of the 95% confidence intervals crosses the Cohen’s *d* small effect size threshold of an effect of 0.2 standard deviations. Equivalence testing confirms strong evidence for the absence of meaningful treatment effects across all outcomes (see Table 2 and Supplementary Information section 2.6.5 for details).

Finally, we explore whether any of the treatment effects differ based on vaccine hesitancy, political attitudes and sociodemographics. We also study potential heterogeneous impacts based on participants’ state of residence, which allows us to examine whether there were unintended consequences in states in which incentive programmes were more or less successful. We find that treatment effects are similarly mute across all subgroups and all states (Supplementary Information sections 2.6.8 and 2.6.9).

## Discussion and conclusions

Our studies document that offering modest payments for vaccination has no sizable unintended consequences. We can reject even small negative impacts of financial incentives for COVID-19 vaccination on people’s future vaccination uptake, other health behaviours, morals and civic responsibility, perceived safety and effectiveness of the vaccines, trust in vaccine providers, and feelings of self-determination and coercion.

Many healthy and prosocial behaviours, such as donating blood, not smoking and vaccinating, have large individual and societal consequences^1,3,5,6,63,64^. Offering financial incentives for behaving healthily and prosocially is widely considered by policy-makers to change behaviour^1,5,31,32,37^. Although offering financial incentives often triggers initial behaviour change, a large academic literature warns against using financial incentives because of unintended consequences. This tension puts policy-makers in a tough spot over the extent to which they should heed or ignore the warnings when considering introducing financial incentives to encourage behaviour change. During the COVID-19 pandemic, for instance, many governments and organizations worldwide offer financial incentives for vaccination, whereas others abstain, worried about grave unintended consequences^37^. Our study provides important evidence that will allow policy-makers to make more informed decisions when weighing the costs and benefits of introducing financial incentives to change behaviour.

Although much of the academic discussion focuses on the negative unintended consequences, financial incentives could, in principle, also have positive unintended consequences^45,48,65^; incentives might not only trigger initial behavioural change but could positively affect future health behaviours, morals, perceptions and feelings. However, we find no support for positive unintended consequences of financial incentives for COVID-19 vaccination.

The evidence from the Swedish RCT and the US state incentive programmes complement each other by using samples with different characteristics, applying different methodologies and studying incentive programmes that differ in scale, incentive type and entity that offers the incentives. Our findings that financial incentives for COVID-19 vaccination do not have negative unintended consequences in both contexts, as well as the lack of consistent negative treatment effects across different sociodemographic and vaccine hesitancy groups, speak to the generalizability of our findings.

However, several limitations remain. First, our studies rely on samples from high-income Western countries. The results may not generalize to low-income countries or to countries with meagre social security systems. Second, to collect encompassing survey data as well as to guarantee compliance with ethical and consent guidelines, participants in our studies were aware that they participated in a study. This awareness could, in principle, affect results but it can hardly be avoided when linking survey with administrative records. Third, our paper focuses on financial incentives for COVID-19 vaccination. Although this is a particularly relevant context given the current debate, we hope that the paper motivates new studies across different contexts (such as organ donation or cancer screening) to improve our understanding of the consequences of offering incentives. Last, although our evidence also informs the normative debate of whether paying for vaccination is ethically permissible^24,25^, ethical debates will not be resolved by empirics alone^66^.

Despite its limitations, our study has a clear finding: offering modest financial incentives for vaccination has limited, if any, negative unintended consequences. Contrary to prominent warnings in the academic literature and public debate, our work suggests that modest financial incentives for vaccination can be used without worries about grave unintended consequences.

## Methods

### Swedish RCT approval and pre-registration

We conducted a pre-registered study with a general population sample of Swedish residents. In two online surveys, we recruited participants from a sample that took part in an earlier RCT^5^. In this earlier RCT, participants were randomly allocated to either a financial incentives condition that offered payments of 200 SEK (about US $24 at the time) conditional on receiving the first dose of a COVID-19 vaccine or a control condition that did not offer any financial incentives (see Supplementary Information section 1.1.5 for details). This earlier RCT provides us with random assignment of participants to financial incentives for taking a first dose. We match the RCT data with exhaustive population-wide Swedish administrative records of COVID-19 vaccinations, which allow us to examine whether and when each of the participants received an unincentivized second dose of a COVID-19 vaccine. We then matched these data with data from two online surveys, in which we measured participants’ health behaviours, morals, perceptions and feelings.

The Swedish ethical review authority (Etikprövningsmyndigheten) approved the protocols of the study (reference number 2021-06367-02). Participants were informed that the study was conducted by researchers and that their data would be matched with vaccination registries by the public health authorities. Informed consent was obtained from all study participants as part of the survey.

We pre-registered the data collection and analysis at the AEA RCT Registry (http://www.socialscienceregistry.org/trials/8727 and http://www.socialscienceregistry.org/trials/9580). Our analysis closely follows the pre-registration plan. In the main analysis, we use the following pre-registered linear regression to estimate treatment effects:

$${Y}_{i}={\beta }_{0}+{\beta }_{1}\times {I}_{i}+{X}_{i}\gamma \text{'}+{{\epsilon }}_{i}$$

in which *Y*_*i*_ captures the outcome variable for participant *i*, *I*_*i*_ is a dummy capturing whether participant *i* is in the financial incentives condition, *β*_1_ estimates the effect of incentives on the outcome variable and *ε*_*i*_ is an individual-specific error. The vector *X*_*i*_ is the vector of pre-registered controls to reduce variability, consisting of participant *i*’s gender, age, region, interactions between age and region, being in an at-risk group for COVID-19, civil status, having children in the household, employment status, education, parents’ place of birth and income (see Supplementary Information section 1.1 for definitions of all variables and further details about the data analysis). We estimate treatment effects using OLS regressions with heteroscedasticity-robust standard errors.

In Supplementary Information section 2.3, we show that the results are robust to including no controls (Extended Data Fig. 1), different sets of control variables, using sample weights and using different inclusion criteria. The battery of further analyses shows no negative unintended consequences of offering financial incentives for vaccination.

### Administrative vaccination records

We use administrative data from COVID-19 national vaccination registers comprising all residents of Sweden. The administrative records include the date of each COVID-19 vaccination of each resident. As it is not possible to opt out of or delete records in the vaccination registry, the administrative records include whether and when each participant received the second dose of a COVID-19 vaccine. Notably, the participants were not offered any payments to take the second dose. Note also that individuals in Sweden had to book the appointments for the first and second doses separately (see Supplementary Information section 1.1.4 for details). The Public Health Agency of Sweden linked the previous RCT data at the individual level with the administrative data on 21 December 2021. As the previous RCT ended on 13 July, we observe for participants whether and when they received the second dose of a COVID-19 vaccine within a time window of 158 days after participation in the trial.

We constructed the following outcomes based on administrative data:

- Second-dose uptake: we measured whether participants took the second dose of a COVID-19 vaccine after participation in the RCT.
- Shorter wait for second dose: we measured how long the participants waited until they received the second dose. For participants who did not take a second dose, we used the maximum wait time that we could observe. We then reverse-coded the outcome so that a positive coefficient indicates shorter wait time.
- Second-dose uptake if first dose: this outcome corresponds to second-dose uptake but we restricted the sample to participants who took a first dose (*n* = 4,358).

We pre-registered second-dose uptake as a main outcome measure based on administrative data.

### Surveys

The survey participants were recruited from a general population panel in Sweden by the survey company Norstat. Norstat actively recruits people by means of phone calls to create a representative panel in terms of age, region and gender. For both surveys, we asked the company to recruit as many participants as possible from the incentives (*n* = 1,131) and control conditions (*n* = 3,888) from the previous RCT (the control condition includes the control and no-reminders conditions from the RCT; see Supplementary Information section 1.1.2 for details). The surveys were programmed in Qualtrics. We provide the questionnaires translated into English in Supplementary Information section 3.1.

Participants in the first survey were paid SEK 50 (about $5.5) for a 10-min survey. Responses were collected in early January 2022. In total, 726 of the participants in the financial incentives condition and 2,512 of the participants in the control condition responded to the survey. Participants in the second survey were paid SEK 10 (about $1) for a 2-min survey. Responses were collected in late June 2022. In total, 606 of the participants in the financial incentives condition and 2,100 of the participants in the control condition responded to the second survey. In both surveys, survey participation was balanced across both conditions, with no differential attrition based on personality characteristics, vaccination status, vaccine hesitancy or sociodemographics (Supplementary Information section 2.1). The survey completion rates were greater than 99% for each survey, with no differences across the incentives and control conditions (Supplementary Information section 2.1).

The participants from the previous RCT are on average 34.6 years old, have an average monthly income of SEK 24,724 and consist of 42% men. In comparison with the Swedish population (in the desired age range of 18–49 years), our sample is representative with respect to age, income and region. However, we have a slight overrepresentation of women and people with a college education and an underrepresentation of people with immigrant background (Supplementary Information section 2.1). In Supplementary Information section 2.3, we show that the results do not change when using sampling weights to adjust for sample composition. In addition, we find that the sociodemographics of participants are comparable across experimental conditions (Supplementary Information section 2.1).

In the first survey, we measured participants’ behaviours, morals, perceptions and feelings related to COVID-19 vaccination. For some outcomes, we aggregated several items into an index, exactly as pre-registered. We measured the following main survey outcomes:

- Third-dose intention: we asked participants whether they are planning to take the third dose of a COVID-19 vaccine (booster shot) when it becomes available to them.
- Third-dose intention for SEK 100/SEK 500: we asked participants to assume that their region pays SEK 100/SEK 500 for everyone who takes the third dose and asked them how likely they would be to take the third dose.
- Flu shot uptake: we asked participants whether they have taken a flu shot in the past 5 months.
- Flu shot intention next season: we asked participants how likely they are to receive a flu shot next season (from fall 2022 to spring 2023).
- Blood donation: we asked participants whether they donated blood in the past 5 months.
- Morals and civic responsibility index: we aggregated the answers to the following items on morals and civic responsibility: (i) I am willing to take the personal costs of receiving a COVID-19 vaccine (such as time, discomfort, mild side effects) for the greater good of society; (ii) I think people have a civic duty or a moral obligation to receive a COVID-19 vaccine; (iii) not taking a COVID-19 vaccine would be generally viewed as socially inappropriate.
- Perceived safety and efficacy index: we aggregated the answers to the following three risk and efficacy perceptions: (i) in general, COVID-19 vaccines are safe; (ii) I am worried about the side effects from COVID-19 vaccines (reverse-coded); (iii) COVID-19 vaccines are highly effective at protecting my health.
- Vaccines generally safe for children: we asked participants whether they think, in general, vaccines given to children, such as the measles vaccine, are safe for healthy children.
- Trust in vaccination provision index: we aggregated the answers to the following three questions on trust: when it comes to the COVID-19 vaccine process, I trust: (i) the pharmaceutical or drug companies; (ii) the researchers studying the effects of the vaccines; (iii) the Public Health Agency of Sweden.
- Feels no regret index: we aggregated the answers to the following two items: (i) we asked participants whether they regret the decision they made on whether to receive the first dose of a COVID-19 vaccine (reverse-coded); (ii) we asked participants whether they gathered enough information to feel well informed about the benefits and risks of the vaccine when deciding to receive the first dose of a COVID-19 vaccine or not. The first question is based on a survey item taken from Ambuehl et al.^67^.
- Lower feelings of coercion: we asked participants whether, when deciding to receive the first dose of a COVID-19 vaccine or not, they felt forced to take or not take the COVID-19 vaccine (reverse-coded). This question is based on a survey item taken from Ambuehl et al.^67^.

To aggregate the individual items into the indices, we standardized each item (subtracted the mean and then divided it by the standard deviation), added the items and divided the result by the number of items. We then standardized all outcomes, including the indices, such that effect sizes are comparable across outcomes.

We also measured the following behaviours, which we standardized for the analysis:

- Donation for vaccination: subjects divided SEK 100 between themselves and the Global Alliance for Vaccines and Immunization. The Global Alliance for Vaccines and Immunization collects donations to provide COVID-19 vaccines in areas with otherwise limited access to vaccination. We incentivized this question by implementing the choice of ten randomly drawn participants.
- Donation for payment for vaccination: subjects divided SEK 100 between themselves and the New Incentives organization. The New Incentives organization is a NGO that attempts to increase vaccination uptake for diseases such as measles by paying people for being vaccinated. We incentivized this question by implementing the choice of ten randomly drawn participants.
- Payment for vaccination is ethical index: we aggregated the answers to the following two items: (i) financial rewards for vaccinating against COVID-19 are unethical (reverse-coded); (ii) I would support the introduction of monetary payments of SEK 500 for those who get vaccinated (or are already vaccinated) against COVID-19. We followed the approach by Elías et al.^68^ and told participants that their views would be shared with policy-makers.

We pre-registered third-dose intention, perceived safety and efficacy index, and morals and civic responsibility index as the main survey outcome variables of this survey.

In the second survey, we measured participants’ third-dose vaccination uptake. We measured the following survey outcomes:

- Third-dose uptake: we asked participants whether they took the third dose of a COVID-19 vaccine.
- Shorter wait for third dose: we asked participants when they took the third dose of a COVID-19 vaccine.
- Third-dose uptake if second dose: this outcome corresponds to “third-dose uptake” but we restricted the sample to participants who took a second dose (*n* = 2,463).

Supplementary Information section 2.4.1 gives the distributions of all survey measures.

### Swedish complementary study

In June 2022, we conducted a pre-registered online study using a general population sample of 1,001 Swedish participants (similar to the sample of the previous RCT; 46% men, average age = 31.56 years, standard deviation = 8.47) recruited by the survey company Norstat. The study examined whether people react differently when they are told that the government or researchers paid people for COVID-19 vaccination. We use the fact that most people in Sweden are unaware of the previous Swedish RCT and that the previous RCT was implemented in collaboration with a governmental organization.

The study randomly allocated participants into two treatment conditions, the government condition and the researcher condition. In both conditions, we first described the earlier RCT. The participants in the researcher condition were then told that “a team of researchers participated in the implementation of the incentive programme”, whereas the participants in the government condition were told that “the Public Health Agency of Sweden participated in the implementation of the incentive programme”. Finally, we measured our outcome measures, represented in Fig. 3. See Supplementary Information section 1.1.6 for a more detailed description of the study.

We pre-registered the data collection and analysis at the AEA RCT Registry (http://www.socialscienceregistry.org/trials/9584). Our analysis closely followed the pre-registration plan. Our analysis has 80% power to detect smaller effects than 0.2 standard deviations at the 5% level, as stated in our pre-registration plan. The Human Subjects Committee of the Faculty of Economics, Business Administration and Information Technology at the University of Zurich approved the protocols of the complementary study (reference number 2022-045). Informed consent was obtained from all study participants as part of the survey.

### US state incentive programmes study

In June and July 2022, we conducted a pre-registered study with 3,062 participants from a general population sample of US residents to study whether COVID-19 financial incentive programmes implemented by US states had negative unintended consequences. We use the fact that many people in the USA (around 62.3% in our sample) are unaware that state governments implemented financial incentive programmes. The survey was programmed in Qualtrics. We provide the questionnaire items in Supplementary Information section 3.

We recruited participants from 12 states that implemented vaccine incentive programmes either at the state or the county level: California, Florida, Illinois, Kentucky, Louisiana, Michigan, Missouri, New York, North Carolina, Ohio, Pennsylvania and Texas (see Supplementary Information sections 1.2.2 and 3.3 for a description of the state incentive programmes).

In the study, we first measured participants’ sociodemographics, including their state of residence in 2021. Next, we measured whether participants knew about the existence of state incentive programmes; we asked them whether, in 2021, any governmental organization in their state offered any financial compensation to people who were vaccinated against COVID-19. We continued by eliciting COVID-19 vaccination history and vaccination attitudes. Finally, we randomly allocated participants into one of two treatment conditions: the incentives condition or the control condition. The participants in the incentives condition received detailed information about their state government’s COVID-19 vaccine incentive programme (Supplementary Information section 3), whereas participants in the control condition did not receive this information. This procedure creates random variation in perceived exposure to incentives, allowing us to study the unintended consequences of being exposed to incentives (for treatment effects on awareness, see Extended Data Fig. 2).

To avoid experimenter demand effects^69^, we elicited the outcome measures in an ostensibly unrelated second study^70^. We blur the connection between the two surveys by letting 4–6 days pass between the two surveys and by using different fonts, formats and university affiliations. In the second survey, we elicited our outcome measures. We measured flu shot intention next season and all survey items included in the morals and civic responsibility index and the perceived safety and efficacy index. In addition, we elicited the following measures:

- Further dose: we asked participants whether they planned to take a further COVID-19 vaccine dose (regardless of the number of doses they received in the past) within the next 6 months.
- Further dose new outbreak: we told participants to assume that there would be a new outbreak of the COVID-19 pandemic in 6 months and the Centers for Disease Control and Prevention would recommend people to take a further COVID-19 vaccine dose (regardless of the number of doses they received in the past). We asked participants whether, in this situation, they would take a further dose.
- Further dose for $20: we told participants to assume that there would be a new outbreak of the COVID-19 pandemic in 6 months, the Centers for Disease Control and Prevention would recommend people to take a further COVID-19 vaccine dose (regardless of the number of doses they received in the past) and that every person receiving a further dose would receive $20. We asked participants whether, in this situation, they would take a further dose.
- Blood donation intention: we asked participants whether they plan to donate blood in the next 6 months.
- Trust in state government: we asked participants how much trust they have in the government of their state of residence when it comes to handling problems.

The survey participants were recruited from a general population panel in the USA by the survey company Prolific. Participants in the first survey were paid $1 for a 4-min survey and participants in the follow-up survey were paid $0.5 for a 2-min survey. In total, 3,980 people responded to the first survey and 3,062 people responded to the follow-up survey. We can therefore match the two surveys for 3,062 participants (50% men, average age = 36.76 years, standard deviation = 13.54, 41% Democrats). Participation in the follow-up survey was balanced across both conditions, with no differential attrition based on vaccination status, vaccine hesitancy or sociodemographics (Supplementary Information section 2.6.1).

We pre-registered the data collection and analysis at the AEA RCT Registry (http://www.socialscienceregistry.org/trials/9607). Our analysis closely follows the pre-registration plan. We use a linear regression to estimate treatment effects using OLS regressions with heteroscedasticity-robust standard errors. We control for gender, age, education, employment status, income and state of residence in 2021. In Supplementary Information section 2.6, we show that the results are robust to including no controls and different sets of control variables. Our analysis has 80% power to detect smaller effects than 0.2 standard deviations at the 5% level, as stated in our pre-registration plan.

The Human Subjects Committee of the Faculty of Economics, Business Administration and Information Technology at the University of Zurich approved the protocols of the study (reference number 2022-045). Participants were informed that the study was conducted by researchers and informed consent was obtained from all study participants as part of the survey.

### Reporting summary

Further information on research design is available in the Nature Portfolio Reporting Summary linked to this article.

## Online content

Any methods, additional references, Nature Portfolio reporting summaries, source data, extended data, supplementary information, acknowledgements, peer review information; details of author contributions and competing interests; and statements of data and code availability are available at 10.1038/s41586-022-05512-4.

### Supplementary information

Supplementary Information This file contains Supplementary Methods, Supplementary Tables and Figures, and Questionnaires.

Reporting Summary

### Source data

Source Data Fig. 1

Source Data Fig. 2

Source Data Fig. 3

Source Data Fig. 4

## Figures and Tables

**Extended Data Table 1:**
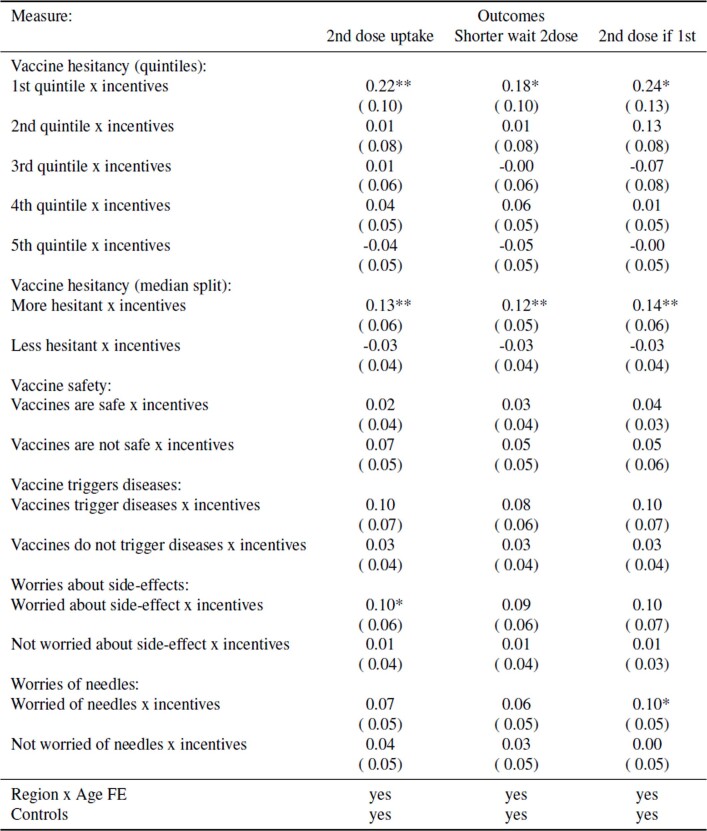
Heterogeneous treatment effects based on vaccine hesitancy on second-dose uptake

The table is based on RCT data linked to population-wide Swedish administrative data on second-dose uptake. The table shows coefficient estimates from linear regressions of the standardized outcome on an indicator for the financial incentives condition interacted with indicators capturing in which quantile a participant’s vaccine hesitancy is (for example, above or below the median). The corresponding coefficients indicate the total effect for each subgroup. Considering all heterogeneity checks, we do not find any robust heterogeneities across 567 coefficient estimates. This holds true whether we use within or between participant specifications. For more details on the specification and interpretation and for the results for the other outcomes, see Supplementary Information section 2.4.3. Heteroscedasticity-robust standard errors (without multiple comparison adjustments) are also shown in parentheses. All regressions use the pre-registered controls consisting of gender, age, region, interactions between age and region, being in an at-risk group for COVID-19, civil status, having children in the household, employment status, education, parents’ place of birth and income. The sample sizes for the control and incentives conditions are *n* incentives = 1,132 and *n* control = 3,888.

**Extended Data Table 2:**
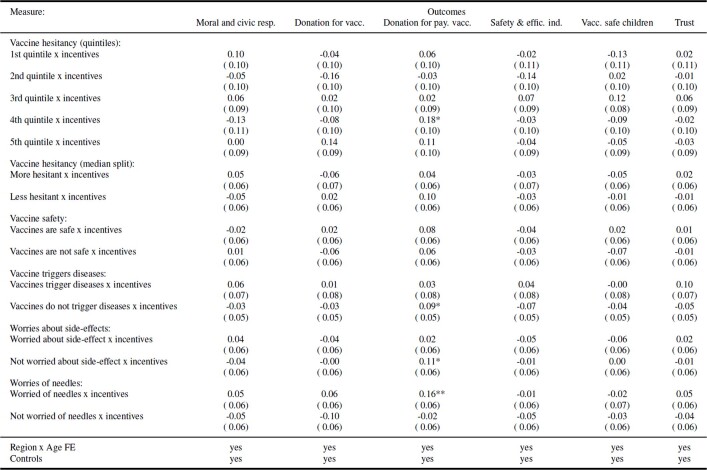
Heterogeneous treatment effects based on vaccine hesitancy on morals and civic responsibility, perceived safety and efficacy, and trust

The table is based on RCT data linked to comprehensive data from the first survey. The table shows coefficient estimates from linear regressions of the standardized outcome on an indicator for the financial incentives condition interacted with indicators capturing in which quantile a participant’s vaccine hesitancy is (for example, above or below the median). The corresponding coefficients indicate the total effect for each subgroup. Considering all heterogeneity checks, we do not find any robust heterogeneities across 567 coefficient estimates. This holds true whether we use within or between participant specifications. For more details on the specification and interpretation and for the results for the other outcomes, see Supplementary Information section 2.4.3. Heteroscedasticity-robust standard errors (without multiple comparison adjustments) are also shown in parentheses. All regressions use the pre-registered controls consisting of gender, age, region, interactions between age and region, being in an at-risk group for COVID-19, civil status, having children in the household, employment status, education, parents’ place of birth and income. The sample sizes for the control and incentives conditions are *n* incentives = 726 and *n* control = 2,512.

**Extended Data Table 3:**
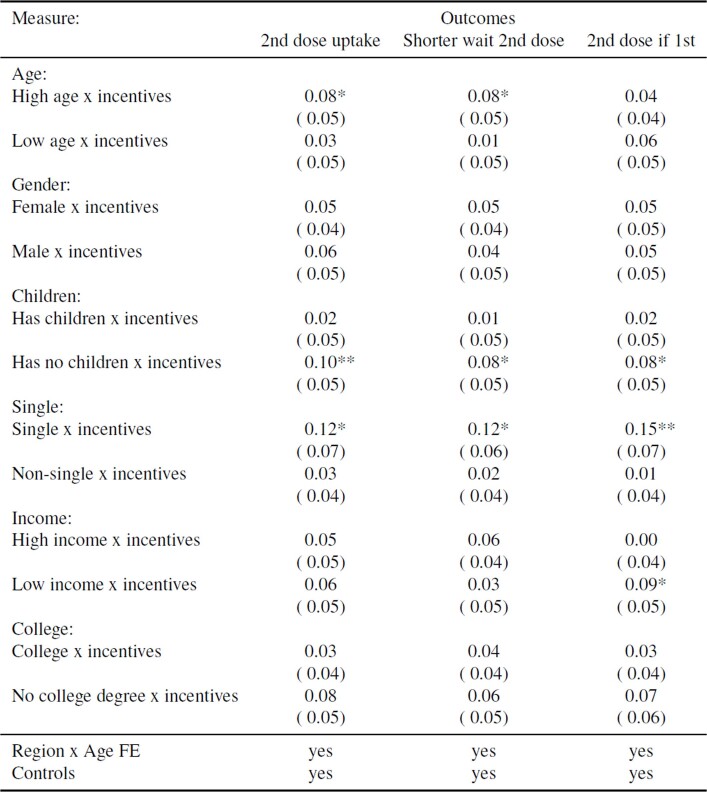
Heterogeneous treatment effects based on sociodemographics on second-dose uptake

The table is based on RCT data linked to population-wide Swedish administrative data on second-dose uptake. The table shows coefficient estimates from linear regressions of the standardized outcome on an indicator for the financial incentives condition interacted with an indicator each for above and below median value of the participant characteristic. The corresponding coefficients indicate the total effect for each subgroup. Considering all heterogeneity checks, we do not find any robust heterogeneities across 567 coefficient estimates. This holds true whether we use within or between participant specifications. For more details on the specification and interpretation and for the results for the other outcomes, see Supplementary Information section 2.4.3. Heteroscedasticity-robust standard errors (without multiple comparison adjustments) are also shown in parentheses. All regressions use the pre-registered controls consisting of gender, age, region, interactions between age and region, being in an at-risk group for COVID-19, civil status, having children in the household, employment status, education, parents’ place of birth and income. The sample sizes for the control and incentives conditions are *n* incentives = 1,132 and *n* control = 3,888.

**Extended Data Table 4:**
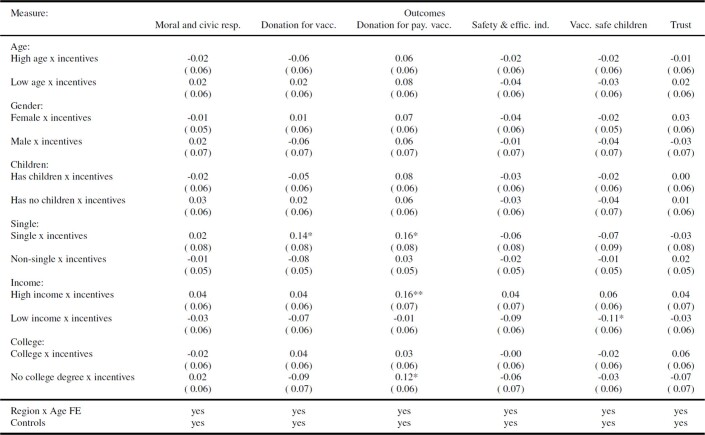
Heterogeneous treatment effects based on sociodemographics on morals and civic responsibility, perceived safety and efficacy, and trust

The table is based on RCT data linked to comprehensive data from the first survey. The table shows coefficient estimates from linear regressions of the standardized outcome on an indicator for the financial incentives condition interacted with an indicator each for above and below median value of the participant characteristic. The corresponding coefficients indicate the total effect for each subgroup. Considering all heterogeneity checks, we do not find any robust heterogeneities across 567 coefficient estimates. This holds true whether we use within or between participant specifications. For more details on the specification and interpretation and for the results for the other outcomes, see Supplementary Information section 2.4.3. Heteroscedasticity-robust standard errors (without multiple comparison adjustments) are also shown in parentheses. All regressions use the pre-registered controls consisting of gender, age, region, interactions between age and region, being in an at-risk group for COVID-19, civil status, having children in the household, employment status, education, parents’ place of birth and income. The sample sizes for the control and incentives conditions are *n* incentives = 726 and *n* control = 2,512.

## Data Availability

The data used in the analyses and figures in the article are available on Zenodo at 10.5281/zenodo.7214856. Source data are provided with this paper.

## Code availability

The code to replicate the analyses and figures in the article is available on Zenodo at 10.5281/zenodo.7214856. Analyses were conducted using STATA 16.

## References

[1] Lacetera N, Macis M, Slonim R (2013). Economic rewards to motivate blood donations. Science.

[2] Stone EG (2002). Interventions that increase use of adult immunization and cancer screening services: a meta-analysis. Ann. Intern. Med..

[3] Volpp KG (2009). A randomized, controlled trial of financial incentives for smoking cessation. N. Engl. J. Med..

[4] Halpern SD (2018). A pragmatic trial of e-cigarettes, incentives, and drugs for smoking cessation. N. Engl. J. Med..

[5] Campos-Mercade P (2021). Monetary incentives increase COVID-19 vaccinations. Science.

[6] Banerjee AV, Duflo E, Glennerster R, Kothari D (2010). Improving immunisation coverage in rural India: clustered randomised controlled evaluation of immunisation campaigns with and without incentives. Br. Med. J..

[7] Alsan M, Garrick O, Graziani G (2019). Does diversity matter for health? Experimental evidence from Oakland. Am. Econ. Rev..

[8] Klüver H, Hartmann F, Humphreys M, Geissler F, Giesecke J (2021). Incentives can spur COVID-19 vaccination uptake. Proc. Natl Acad. Sci..

[9] Serra-Garcia, M. & Szech, N. Incentives and defaults can increase COVID-19 vaccine intentions and test demand. *Manage. Sci.*10.1287/mnsc.2022.4405 (2022).

[10] Volpp KG (2008). Financial incentive-based approaches for weight loss: a randomized trial. JAMA.

[11] Charness G, Gneezy U (2009). Incentives to exercise. Econometrica.

[12] Sandel, M. *What Money Can’t Buy: The Moral Limits of Markets* (Macmillan, 2012).

[13] Titmuss, R. M. *The Gift Relationship: From Human Blood to Social Policy* (Policy Press, 1970). 4670163

[14] Satz, D. *Why Some Things Should Not Be for Sale: The Moral Limits of Markets* (Oxford Univ. Press, 2010).

[15] Bowles S (2008). Policies designed for self-interested citizens may undermine “the moral sentiments”: evidence from economic experiments. Science.

[16] Frey BS, Jegen R (2001). Motivation crowding theory. J. Econ. Surv..

[17] Largent EA, Miller FG (2021). Problems with paying people to be vaccinated against COVID-19. JAMA.

[18] Frey BS, Oberholzer-Gee F (1997). The cost of price incentives: an empirical analysis of motivation crowding-out. Am. Econ. Rev..

[19] Bénabou R, Tirole J (2003). Intrinsic and extrinsic motivation. Rev. Econ. Stud..

[20] Ellingsen T, Johannesson M (2008). Pride and prejudice: the human side of incentive theory. Am. Econ. Rev..

[21] Cryder CE, London AJ, Volpp KG, Loewenstein G (2010). Informative inducement: study payment as a signal of risk. Soc. Sci. Med..

[22] Volpp, K. G. & Cannuscio, C. C. Incentives for immunity—strategies for increasing COVID-19 vaccine uptake. *N. Engl. J. Med.* **385**, e1 (2021). 10.1056/NEJMp2107719 34038633

[23] Deci EL (1971). Effects of externally mediated rewards on intrinsic motivation. J. Pers. Soc. Psychol..

[24] Jecker NS (2021). Cash incentives, ethics, and COVID-19 vaccination. Science.

[25] Savulescu J (2021). Good reasons to vaccinate: mandatory or payment for risk?. J. Med. Ethics.

[26] Fehr E, Rockenbach B (2003). Detrimental effects of sanctions on human altruism. Nature.

[27] Böhm R (2022). Crowdsourcing interventions to promote uptake of COVID-19 booster vaccines. EClinicalMedicine.

[28] Falk A, Kosfeld M (2006). The hidden costs of control. Am. Econ. Rev..

[29] Roth AE (2007). Repugnance as a constraint on markets. J. Econ. Perspect..

[30] Gneezy U, Rustichini A (2000). Pay enough or don't pay at all. Q. J. Econ..

[31] World Health Organization (WHO). *The Melbourne Declaration on 100% Voluntary Non-remunerated Donation of Blood and Blood Components*. https://www.who.int/publications/m/item/who-global-consultation-100-voluntary-non-remunerated-blood-donation-of-blood-and-blood-components (WHO, 2009).

[32] European Centre for Disease Prevention and Control (ECDC). *Facilitating COVID-19 Vaccination Acceptance and Uptake in the EU/EEA* (ECDC, 2021).

[33] Haushofer J, Metcalf CJE (2020). Which interventions work best in a pandemic?. Science.

[34] Dai H (2021). Behavioural nudges increase COVID-19 vaccinations. Nature.

[35] Saccardo, S. et al. Assessing nudge scalability: two lessons from large-scale RCTs. *SSRN*, 10.2139/ssrn.3971192 (2022).

[36] Bartoš V, Bauer M, Cahlíková J, Chytilová J (2022). Communicating doctors’ consensus persistently increases COVID-19 vaccinations. Nature.

[37] Oza A (2021). Studies probe how payouts affect U.S. vaccination rates. Science.

[38] Loewenstein, G. & Cryder, C. Why paying people to be vaccinated could backfire. *The New York Times* https://www.nytimes.com/2020/12/14/upshot/covid-vaccine-payment.html (2020).

[39] Persad G, Emanuel EJ (2021). Ethical considerations of offering benefits to COVID-19 vaccine recipients. JAMA.

[40] Thaler, R. More than nudges are needed to end the pandemic. *The New York Times* https://www.nytimes.com/2021/08/05/business/vaccine-pandemic-nudge-passport.html (2021).

[41] Volpp KG, Loewenstein G, Buttenheim AM (2021). Behaviorally informed strategies for a national COVID-19 vaccine promotion program. JAMA.

[42] Brewer NT, Chapman GB, Rothman AJ, Leask J, Kempe A (2018). Increasing vaccination: putting psychological science into action. Psychol. Sci. Public Interest.

[43] Gneezy U, Meier S, Rey-Biel P (2011). When and why incentives (don’t) work to modify behavior. J. Econ. Perspect..

[44] Lacetera N, Macis M, Slonim R (2014). Rewarding volunteers: a field experiment. Manage. Sci..

[45] Goette L, Stutzer A (2020). Blood donations and incentives: evidence from a field experiment. J. Econ. Behav. Organ..

[46] Becker GS, Elías JJ (2007). Introducing incentives in the market for live and cadaveric organ donations. J. Econ. Perspect..

[47] Berlin I (2021). Financial incentives for smoking cessation in pregnancy: multicentre randomised controlled trial. Br. Med. J..

[48] Milkman KL (2021). Megastudies improve the impact of applied behavioural science. Nature.

[49] Barankay I (2020). Effect of patient financial incentives on statin adherence and lipid control: a randomized clinical trial. JAMA Netw. Open.

[50] Halpern SD (2021). Effectiveness and ethics of incentives for research participation: 2 randomized clinical trials. JAMA Intern. Med..

[51] Hyun I (2006). Fair payment or undue inducement?. Nature.

[52] Facciorusso A, Demb J, Mohan BP, Gupta S, Singh S (2021). Addition of financial incentives to mailed outreach for promoting colorectal cancer screening: a systematic review and meta-analysis. JAMA Netw. Open.

[53] Berger RL, Hsu JC (1996). Bioequivalence trials, intersection-union tests and equivalence confidence sets. Stat. Sci..

[54] Lakens D (2017). Equivalence tests: a practical primer for t tests, correlations, and meta-analyses. Soc. Psychol. Per. Sci..

[55] Rabb N (2022). Evidence from a statewide vaccination RCT shows the limits of nudges. Nature.

[56] Jacobson M, Chang TY, Shah M, Pramanik R, Shah S (2022). Can financial incentives and other nudges increase COVID-19 vaccinations among the vaccine hesitant? A randomized trial. Vaccine.

[57] Thirumurthy, H., Milkman, K. L., Volpp, K. G., Buttenheim, A. M. & Pope, D. G. Association between statewide financial incentive programs and COVID-19 vaccination rates. *PLoS ONE* **17**, e0263425 (2022). 10.1371/journal.pone.0263425 PMC8966995 35353815

[58] Brewer NT (2022). Incentives for COVID-19 vaccination. Lancet Reg. Health Americas.

[59] Acharya B, Dhakal C (2021). Implementation of state vaccine incentive lottery programs and uptake of COVID-19 vaccinations in the United States. JAMA Netw. Open.

[60] Barber A, West J (2022). Conditional cash lotteries increase COVID-19 vaccination rates. J. Health Econ..

[61] Milkman, K. L. et al. A citywide experiment testing the impact of geographically targeted, high-pay-off vaccine lotteries. *Nat. Hum. Behav.*10.1038/s41562-022-01437-0 (2022). 10.1038/s41562-022-01437-0 36050387

[62] Robertson C, Schaefer KA, Scheitrum D (2021). Are vaccine lotteries worth the money?. Econ. Lett..

[63] Campos-Mercade P, Meier AN, Schneider FH, Wengström E (2021). Prosociality predicts health behaviors during the COVID-19 pandemic. J. Public Econ..

[64] Andersson O, Campos-Mercade P, Meier AN, Wengström E (2021). Anticipation of COVID-19 vaccines reduces willingness to socially distance. J. Health Econ..

[65] Royer H, Stehr M, Sydnor J (2015). Incentives, commitments, and habit formation in exercise: evidence from a field experiment with workers at a Fortune-500 company. Am. Econ. J. Appl. Econ..

[66] Ngo S, Kim AS, Chiong W (2021). Evidence for the ethics of incentivizing clinical trial enrollment?. JAMA Intern. Med..

[67] Ambuehl S, Niederle M, Roth AE (2015). More money, more problems? Can high pay be coercive and repugnant?. Am. Econ. Rev..

[68] Elías JJ, Lacetera N, Macis M (2019). Paying for kidneys? A randomized survey and choice experiment. Am. Econ. Rev..

[69] Rosenthal, R. *Experimenter Effects in Behavioral Research* (Appleton-Century-Crofts, 1969).

[70] Haaland, I., Roth, C. & Wohlfart, J. Designing information provision experiments. *J. Econ. Lit.* (2021).

