# Supplementary material for: Financial incentives for vaccination do not have negative unintended consequences
Source: Nature. 2023 Jan 11;613(7944):526–33. doi: 10.1038/s41586-022-05512-4 (PMC9833033; doi:10.1038/s41586-022-05512-4)
Supplement: Supplementary file 1 — This file contains Supplementary Methods, Supplementary Tables and Figures, and Questionnaires. [file 41586_2022_5512_MOESM1_ESM.pdf]

nature portfolio

https://doi.org/10.1038/s41586-022-05512-4

Supplementary information

Financial incentives for vaccination do not have negative unintended consequences

In the format provided by the authors and unedited

Supplementary Information for

Financial incentives for vaccination do not have negative unintended consequences

Florian H. Schneider*, Pol Campos-Mercade*, Stephan Meier,
Devin Pope, Erik Wengström, Armando N. Meier*

*Equal lead authors, correspondence to:
florian.schneider2@uzh.ch, pol.campos@nek.lu.se, armando.meier@unil.ch.

This PDF file includes:

Supplementary Methods
Supplementary Tables and Figures
Questionnaires

Detailed list of contents:

1 Supplementary Methods 9
1.1 Swedish study 9
1.1.1 Sample size and power 9
1.1.2 Exclusion criteria 9
1.1.3 Definition of control variables 10
1.1.4 Swedish setting 11
1.1.5 Detailed description of the previous RCT in Sweden 12
1.1.6 Swedish complementary study 13
1.2 US study 15
1.2.1 Sample size and power 15
1.2.2 Description of the US incentive programs 15

2 Supplementary Tables and Figures 17
2.1 Summary statistics, balance, survey participation, and sample 17
2.1.1 Summary statistics 17
2.1.2 Balance and survey participation 21
2.1.3 General population sample 27
2.2 Main results 28
2.2.1 Regression results for main outcome variables 28
2.2.2 Treatment effects on second dose uptake only using data from survey participants 32
2.2.3 Timing of second dose uptake 34
2.2.4 Equivalence testing 35
2.3 Robustness checks 37
2.3.1 Different sets of controls 37
2.3.2 Sample weights 41
2.3.3 Including participants who did not finish the survey 42
2.4 Additional results 43
2.4.1 Distribution of raw outcomes depending on condition 43
2.4.2 Treatment effects on dispersion of outcome variables 48
2.4.3 Treatment effects based on participants' characteristics 49

2.4.4 Results for single items of indices 61
2.4.5 Secondary and exploratory outcome variables 64
2.5 Results Swedish complementary study 66
2.5.1 Overview 66
2.5.2 Summary statistics 66
2.5.3 General population sample 67
2.5.4 Treatment effects 68
2.5.5 Equivalence testing 69
2.5.6 Different sets of controls 70
2.5.7 Treatment effects for individuals that were not aware of the Swedish RCT. 73
2.5.8 Treatment effects based on participants' characteristics 73
2.5.9 Treatment effects selection experiment 77
2.6 Results US study 78
2.6.1 Balance and survey participation 78
2.6.2 Summary statistics 79
2.6.3 Treatment effects on awareness 80
2.6.4 Treatment effects 83
2.6.5 Equivalence testing 85
2.6.6 Different sets of controls 86
2.6.7 Treatment effects for individuals that were not aware of US state incentive programs. 89
2.6.8 Treatment effects based on participants' characteristics 91
2.6.9 Treatment effects based on state 97
2.6.10 Results for single items of indices 103

3 Questionnaires **105**
3.1 Surveys Sweden 105
3.1.1 Consent form 105
3.1.2 Outcome measures first survey 107
3.1.3 Outcome measures second survey 110
3.2 Swedish complementary study 110
3.2.1 Consent Form 110
3.2.2 Vaccine history and attitudes 111

3.2.3 Information of the incentive program 112
3.2.4 Filler questions (preferences and sociodemographics) 113
3.2.5 Outcome measures 113
3.3 US study 114
3.3.1 Consent form 114
3.3.2 Sociodemographics, beliefs about existence of state incentive program and vaccination attitudes 115
3.3.3 Incentives condition 117
3.3.4 Outcome measures 121
3.3.5 Awareness incentive programs 123

4 References **124**

Figs. S1 to S34

S1 Treatment effects on 2nd dose uptake for only participants first survey 32
S2 Treatment effects on 2nd dose uptake for only participants second survey 33
S3 Proportion of participants who got the 2nd dose of a COVID-19 vaccine per day after the previous trial 34
S4 Different controls: no controls 37
S5 Different controls: controlling for region x age fixed effects (FE) 38
S6 Different controls: controlling for region x age fixed effects and gender 38
S7 Different controls: controlling for region x age FE, gender, and sociodemographics (education groups, income groups, occupation groups, parental origin) 39
S8 Different controls: controlling for region x age FE, sociodemographics, and family characteristics (has children, marital status) 39
S9 Main specification: controlling for region x age FE, sociodemographics, family characteristics, and COVID-19 risk group status 40
S10 Treatment effects with sample weights 41
S11 Treatment effects including participants who did not finish the survey 42
S12 Distribution of raw outcomes depending on condition, Part 1 44
S13 Distribution of raw outcomes depending on condition, Part 2 45
S14 Distribution of raw outcomes depending on condition, Part 3 46
S15 Distribution of raw outcomes depending on condition, Part 4 47
S16 Treatment effects on dispersion of outcome variables 48
S17 Indices and single items 63
S18 Treatment effects Swedish complementary study 69
S19 Different controls: no controls 71
S20 Different controls: controlling for age 71
S21 Different controls: controlling for age and gender 72
S22 Different controls: controlling for age, gender and education 72
S23 Focusing on individuals that were not aware of the Swedish RCT 73
S24 Treatment effects selection experiment 77
S25 Distribution awareness of state incentive programs 81
S26 Distribution awareness of specific state incentive program 82
S27 Treatment effects on awareness 82

S28 Treatment effects US study . . . 83
S29 Different controls: no controls . . . 87
S30 Different controls: controlling for state fixed effects . . . 87
S31 Different controls: controlling for state fixed effects, age, gender and ethnicity . . . 88
S32 Different controls: controlling for state fixed effects, age, gender, ethnicity, education and employment status . . . 88
S33 Focusing on individuals that were not aware of the state incentive programs . . . 91
S34 Treatment effects on each single item of the indices . . . 104

Tables S1 to S59

S1 US incentive programs . . . 16
S2 Summary statistics . . . 18
S3 First survey participation and completion by condition . . . 23
S4 First survey participation based on COVID-19 related variables . . . 23
S5 First survey participation based on personality and economic preferences . . . 24
S6 First survey participation based on sociodemographics . . . 24
S7 Second survey participation and completion by condition . . . 24
S8 Second survey participation based on COVID-19 related variables . . . 25
S9 Second survey participation based on personality and economic preferences . . . 26
S10 Second survey participation based on sociodemographics . . . 26
S11 Trial sample, survey sample, and the Swedish population . . . 27
S12 Treatment effects on 2nd dose uptake . . . 28
S13 Treatment effects on 3rd dose uptake . . . 29
S14 Treatment effects on other health behaviors . . . 29
S15 Treatment effects on morals and civic responsibility . . . 30
S16 Treatment effects on perceived safety, efficacy, and trust . . . 30
S17 Treatment effects on other concerns . . . 31
S18 Equivalence testing . . . 36
S19 Heterogeneous treatment effects based on vaccine hesitancy on 2nd dose uptake . . . 53
S20 Heterogeneous treatment effects based on vaccine hesitancy on 3rd dose uptake . . . 54
S21 Heterogeneous treatment effects based on vaccine hesitancy on morals and civic responsibility, perceived safety and efficacy, and trust . . . 55

S22 Heterogeneous treatment effects based on vaccine hesitancy on other health behaviors and feelings of regret 56
S23 Heterogeneous treatment effects based on sociodemographics on 2nd dose uptake . 57
S24 Heterogeneous treatment effects based on sociodemographics on 3rd dose uptake . 58
S25 Heterogeneous treatment effects based on sociodemographics on morals and civic responsibility, perceived safety and efficacy, and trust 59
S26 Heterogeneous treatment effects based on sociodemographics on other health behaviors and feelings of regret 59
S27 Within participant changes in worries about side-effects and safety perceptions . . . 60
S28 Single items: morals and civic responsibility 61
S29 Single items: safety and efficacy 61
S30 Single items: trust in vaccination provision 62
S31 Single items: payment for vaccination is ethical 62
S32 Single items: feelings of regret 63
S33 Secondary and exploratory outcomes 64
S34 Additional outcomes on 3rd dose intention 65
S35 Summary statistics 67
S36 Trial sample, survey sample, and the Swedish population 68
S37 Treatment effects Swedish complementary study 69
S38 Equivalence testing 70
S39 Heterogeneous treatment effects Swedish complementary study 76
S40 Participation follow-up survey by condition 78
S41 Survey participation based on COVID-19 related variables 79
S42 Survey participation based on sociodemographics 79
S43 Summary statistics 80
S44 Treatment effects US study (COVID-19 vaccination) 84
S45 Treatment effects US study (Other health behavior) 84
S46 Treatment effects US study (Perceived safety and morals) 85
S47 Equivalence testing 86
S48 Awareness of state incentive programs among different groups 90
S49 Awareness of specific state incentive programs among different groups 90
S50 Heterogeneous treatment effects based on vaccine attitudes 94

S51 Heterogeneous treatment effects based on vaccine attitudes 95
S52 Heterogeneous treatment effects based on sociodemographics 96
S53 Heterogeneous treatment effects based on sociodemographics 97
S54 Heterogeneous treatment effects based on state 100
S55 Heterogeneous treatment effects based on state 101
S56 Heterogeneous treatment effects based on state 102
S57 Heterogeneous treatment effects based on state 102
S58 Heterogeneous treatment effects based on state 103
S59 Heterogeneous treatment effects based on state 103

1 Supplementary Methods

1.1 Swedish study

1.1.1 Sample size and power

We aimed to have enough power to detect even small impacts of offering financial incentives on behaviors, morals, perceptions, and feelings. Hence, we asked the survey company Norstat to recruit as many participants as possible who participated in the incentives (N=1,131) and control conditions (N=3,888) in the previous RCT, which was also implemented in the panel by Norstat. The reason for the higher number of observations in the control condition is that the previous trial oversampled the control condition.

We use the full sample (N=5,019) to study treatment effects on second dose vaccination uptake using the administrative data. This sample gives us 80% power to detect an effect size (= Cohen's d) of about 0.08 standard deviations.

We pre-registered that we would expect around 3,000 participants to participate in the first survey. This would give us 80% power to detect an effect size (= Cohen's d) of about 0.11 standard deviations for the survey outcome measures. At the end of data collection, the survey company was able to recruit 3,238 participants who completed the survey.

We pre-registered that we would expect around 2,500 participants to participate in the second survey. The reason we expected fewer participants than in the first survey is that people naturally drop out of the panel over time. This would give us 80% power to detect an effect size of about 0.12 standard deviations for the survey outcome measures. At the end of data collection, the survey company was able to recruit 2,706 participants who completed the survey.

1.1.2 Exclusion criteria

When analyzing the Swedish administrative records, we include the participants who in the RCT were in the Incentives, Control, and No reminders conditions, as pre-registered. We invited all of these individuals to participate in the two surveys. Participants in the Incentives condition in the earlier RCT form the *financial incentives condition*, and participants in the Control and No reminders conditions form the *control condition*. The reason we include both Control and No Reminders in the control condition is that none of them included any nudge or financial incentive, and participants' vaccination uptake was not affected by the reminders (*3*).

When analyzing the survey outcome variables, we include all individuals who completed the surveys. The survey completion rates were over 99% for each survey, with no differences across the financial incentives and control condition.

1.1.3 Definition of control variables

The pre-registered controls consist of each participant's gender, age, region, interactions between age and region, being in an at-risk group for COVID-19, civil status, having children in the household, employment status, education, parents' place of birth, and income. We constructed all variables exactly as pre-registered:

- Gender: dummies for the categories indicating male/female.
- Age: We first construct an indicator for each age group capturing the following ages: 18-19, 20-24, 25-29, 30-34, 35-39, 40-44, or 45-49. In all regressions with pre-registered controls, we then control for a fixed effect for each of those age groups and for each region. We also control for the 161 interactions of each region fixed effect with each age-group fixed effect.
- Region: dummies for each of the 21 counties in Sweden.
- Being in a COVID-19 risk group: dummy for the category "yes."
- Civil status dummies for each status: single, sarbo, couple, married, others.
- A dummy for whether children live in the participant household: dummy for number of children in the household > 0.
- Employment status dummies for each status: full-time, part-time, work, unemployed, student, pensioner, others.
- Educational attainment dummies for each group: elementary, high-school, professional training, ongoing university studies, university studies, research studies.
- Parental place of birth dummies: dummies for each place of origin of the mother and the father. (Sweden, Another European country, North America, South America, Africa, Middle East, Rest of Asia, Oceania)
- Income dummies for each category of incomes used in the survey. (0-5000kr, 5001-10000kr, 10001-15000kr, 15001-20000kr, 20001-25000kr, 25001-30000kr, 30001-35000kr, 35001-

40000kr , 40001-45000kr, 45001-50000kr, 50000kr-55000kr, more than 55000kr, coded as the midpoint value for the values below 55000kr and for the ones above as 60000kr)

These variables were measured in the previous RCT before offering incentives. The RCT data also includes information on economic preferences and personality (altruism, reciprocity, trust, patience, and risk-affinity, adherence to social norms), safety perception of COVID-19 vaccines, and vaccine knowledge in general.

1.1.4 Swedish setting

Vaccination against COVID-19 in Sweden is administered by 21 regional health authorities, coordinated by the Public Health Agency of Sweden through recommendations about priority groups and other aspects of the vaccination campaign. In 2021, vaccination was offered to all adults living in Sweden free of charge. For the age groups that our participants belong to (18-49), only the Pfizer-BioNTech and Moderna vaccines were used. While the Swedish government did not officially state what percentage of the population it aimed to vaccinate, the authorities repeatedly recommended that every eligible adult should be vaccinated. The Swedish government implemented very few restrictions for people who had taken fewer than two vaccine doses. Restrictions only applied for indoor public gatherings and events, where people were required to show a vaccination certificate if there were more than 50 participants.

For the age groups that our participants belong to, the first dose of the COVID-19 vaccine became available between May 2021 and July 2021. In most regions, individuals had to book the appointment for the first and second dose independently. They first booked and received the first dose. After receiving the first dose, they could make a separate appointment to get the second dose. The authorities recommended booking a second dose at least 7 weeks after receiving the first dose. The rollout of the third dose for those aged 18 to 49 started in early-January 2022, with a required interval of at least six months between the second and third dose. The recommended shortest interval between doses 2 and 3 was reduced to three months on January 12, 2022. At the time of the survey at the beginning of January 2022, less than 10% of the population aged 18 to 49 had received a third dose.

The vaccination data from the regions are sent to the Public Health Agency of Sweden and included in the National Vaccination Register (*Nationella vaccinationsregistret*). The vaccination data we use was extracted by the Public Health Agency of Sweden on December 21, 2021, and linked to our trial data by the Public Health Agency of Sweden.

Sweden is similar to other western countries in terms of vaccination rates (*72*), inequality (*73*), and economic preferences such as prosociality (*74*, *75*).

1.1.5 Detailed description of the previous RCT in Sweden

We conducted the previous RCT (*5*) with a general population sample of Swedish residents. In an online survey conducted with the panel of the survey company Norstat, we randomly allocated participants to interventions designed with the goal to increase vaccination uptake. We matched the data from the online survey with population-wide Swedish administrative records for vaccinations, which allowed us to examine whether the participants got vaccinated. We asked the company to recruit as many participants as possible between 18-49 years old. (We did not recruit participants older than 50 because the rollout for ages 50 and older started before we received ethics approval.) Participants were asked to fill out an online survey, and responses were collected between May 28, 2021 and July 13, 2021. We ended up with a sample of 8,286 participants. The Swedish ethical review authority (Etikprövningsmyndigheten) approved the protocols of the randomized controlled trial (reference number 2021-01658). Informed consent was obtained from all study participants as part of the enrollment process.

Participants first answered a series of survey questions on personal characteristics such as socio-demographics. We then randomly assigned participants to one of six experimental conditions: the Incentives, Control, and No reminders conditions, and three additional conditions that included nudges to increase vaccination uptake. To study the the unintended consequences of offering financial incentives we only focus on participants who were in the Incentives, Control, and No reminders conditions, as pre-registered. Hence, in the following we will focus on these three conditions; for a description of the nudges conditions see Campos-Mercade et al. (*5*).

We allocated 1/3 of all participants to the Control condition. We oversampled the control condition because we were particularly interested in understanding which experimental conditions would increase vaccination uptake relative to the control condition. Given our interest in this comparison, power calculations indicated that oversampling the control condition increased power.

As in all experimental conditions except the No reminders condition, we encouraged participants to vaccinate within 30 days after the vaccine became available to them ("We would like to encourage you to get a COVID-19 vaccine as soon as possible, ideally within the first 30 days after the vaccine becomes available to you"), we included a link where they could get informa-

tion on how to book an appointment for their vaccination, and we sent two reminders with the encouragement and the link within the following four weeks.

To study the impact of monetary incentives on vaccination uptake, we allocated 2/15 of participants to the Incentives condition. In the Incentives condition, we offered participants SEK 200 (around USD 24 at the time of data collection) if they got vaccinated within 30 days after they were eligible to get vaccinated or, in case they were already eligible, 30 days after they filled out the survey. We informed participants that we would check whether they got vaccinated using administrative data.

To study the impact of different behavioral nudges on vaccination uptake, we allocated 2/15 of participants to each of the three nudge conditions (see Campos-Mercade et al, 3).

Finally, we allocated 2/15 of participants to the No reminders condition. In contrast to all other conditions, the No reminders condition did not include an encouragement to vaccinate, did not include a link to schedule an appointment, and did not include reminders. However, as in the Control condition, the No reminders condition did not include any specific nudge.

1.1.6 Swedish complementary study

Using a general population sample of 1,001 Swedish participants (similar to the sample of the previous RCT), we conducted a pre-registered online study in June 2022 to examine whether people react differently when they are told that the government or researchers paid people for COVID-19 vaccination. We use the facts that most people in Sweden are unaware of the previous Swedish RCT and that the previous RCT was implemented in collaboration with a governmental organization. We provide the questionnaire items translated to English in SI section 3.

In the study, we first measured participants' COVID-19 vaccination history and vaccination attitudes. Next, we randomly allocated participants into two treatment conditions, the government condition and the researcher condition. In both conditions, we first described the earlier RCT. The participants in the researcher condition were then told that "A team of researchers participated in the implementation of the incentive program" while the participants in the government condition were told that "The Public Health Agency of Sweden participated in the implementation of the incentive program." To avoid experimenter demand effects (*70*), we next added a screen of filler questions unrelated to COVID-19 to obfuscate the purpose of the study. Finally, we measured our pre-registered outcome measures: we elicited all survey items included in the "Morals and civic responsibility index" and the "Perceived safety and efficacy index" (see section 'Surveys on morals,

perceptions, feelings, and health behaviors' in the main text). In addition, we elicited participants' planned future COVID-19 vaccination uptake:

- **Additional dose**: We asked participants whether they planned to take an additional COVID-19 vaccine shot (regardless of the number of shots they got in the past) within the next 6 months.

- **Additional dose new outbreak**: We told participants to assume that there would be a new outbreak of the COVID-19 pandemic in 6 months and the Swedish health authorities would recommend people to take an additional COVID-19 vaccine shot (regardless of the number of shots they got in the past). We asked participants whether, in this situation, they would take an additional shot.

The survey participants were recruited from a general population panel in Sweden (representative in terms of age, region, and gender) by the survey company Norstat and were paid SEK 4 (about $0.4) for a 4 minutes survey. In comparison with the Swedish population, our sample is representative with respect to age, gender, income, and education (see SI section 2.5.3). These variables are balanced across treatment conditions.

We pre-registered the data collection and analysis at the AEA RCT Registry (www.socialscienceregistry.org/trials/9583 and www.socialscienceregistry.org/trials/9584). Our analysis closely follows the pre-registration plan. We use a linear regression to estimate treatment effects using ordinary least squares regressions with heteroscedasticity-robust standard errors. We control for gender, age, education and income. In SI section 2.5 we show that results are robust to including no control variables and different sets of control variables. Our analysis has 80% power to detect even small effects of 0.2 standard deviations at the 5% level, as stated in our pre-registration plan.

The Swedish complementary study contained an additional small experiment. Participants in the previous RCT were informed in the consent form that the study was about COVID-19 vaccination, which could potentially lead to selection issues. To address this potential concern of the RCT data, we randomly allocated participants to a condition where the consent form mentions that the study is about COVID-19 vaccination or to a condition without this information (with the same wording used in the original study; see SI Section 3). We find that mentioning COVID-19 vaccination in the consent form did not impact participation rates or selection into the study (see SI section 2.5.9).

The Human Subjects Committee of the Faculty of Economics, Business Administration, and Information Technology at University of Zurich approved the protocols of the study (reference numbers 2022-045). Informed consent was obtained from all study participants as part of the survey.

1.2 US study

1.2.1 Sample size and power

We aimed to have enough power to detect even small impacts of informing participants about the incentive programs implemented in their state on behaviors, morals, and perceptions about the COVID-19 vaccines.

We pre-registered that we would expect around 3,000 participants to participate in both the baseline and the follow-up surveys. This would give us 80% power to detect an effect size (= Cohen's d) smaller than 0.2 standard deviations for our outcome measures. At the end of the data collection, we recruited 3,062 participants.

1.2.2 Description of the US incentive programs

We recruited participants from twelve states that implemented incentive programs for COVID-19 vaccination either at the state or county level: California, Florida, Illinois, Kentucky, Louisiana, Michigan, Missouri, New York, North Carolina, Ohio, Pennsylvania and Texas. The following table provides a description of these twelve incentive programs (for a more detailed description of all US state incentive programs for COVID-19 vaccination, see *56*). Incentive programs differed by the type of incentive (participation in a lottery or a guaranteed payment), the size of the incentives, eligibility (only newly vaccinated or all vaccinated residents) and the entity providing the incentive (state or county). Table S1 provides an overview of the incentive programs in the twelve states that we use in our analysis.

Table S1: US incentive programs

State	Eligibility	Incentive type	State or county level	Description program
California	New	Lottery + Guaranteed	State	Two Million $50 gift card<br>+ Ten $1.5 Million prizes<br>+ 30 $50,000 prizes
Florida	New	Guaranteed	County<br>(Alachua, Flagler)	$25 gift cards<br>+ $10 food coupons
Illinois	All	Lottery	State	Three $1 Million prizes<br>+ 40 $100'000 prizes<br>+ 20 $150,000 scholarships
Kentucky	All	Lottery	State	Three $1 Million prizes<br>+ 15 scholarships
Louisiana	All	Guaranteed	State	$100
Michigan	All	Lottery	State	One $2 Million prize<br>+ one $1 Million prize<br>+ 30 $50,000 prizes<br>+ nine $55,000 scholarships
Missouri	All	Lottery	State	900 $10,000 prizes
New York	New	Lottery	State	One $5 Million prize
North Carolina	All	Lottery	State	Four $1 Million prizes
Ohio	All	Lottery	State	Five $1 Million prizes<br>+ five scholarships
Pennsylvania	New	Guaranteed	County<br>(Philadelphia)	$100
Texas	New	Lottery	County<br>(Houston)	14 $1,000 prizes

2 Supplementary Tables and Figures

In this section, we provide the key tables and figures that are not shown in the main text. Sections 2.1 to 2.4 focus on the Swedish study. First, we provide summary statistics for our variables, show that our sample is balanced across the financial incentives and control conditions, and study its representativeness by comparing the sociodemographics of our sample to the Swedish population. Second, we provide tables with the results that we illustrate in Fig. 1 of the main text and a battery of robustness checks. Finally, we provide additional results, including whether incentives had heterogeneous treatment effects for participants with different characteristics. Section 2.5 discusses the results of the Swedish complementary study and Section 2.6 focuses on the US study.

2.1 Summary statistics, balance, survey participation, and sample

In this section, we provide an overview of the data for the Swedish study. We first provide summary statistics of all of our outcome and control variables. Next, we study whether participants are comparable across treatment conditions based on their background characteristics. Finally, we use data from Statistics Sweden to study the representativeness of our sample.

2.1.1 Summary statistics

Table S2 below provides descriptive statistics of our sample. The table first reports the summary statistics for our outcome variables. Note that all the presented indices are standardized. All other outcome variables are not standardized in Table S2, but are standardized for all analyses. We then report the proportion randomly assigned to the financial incentives treatment. Finally, we report the summary statistics for the background variables, including participants' sociodemographics and answers to vaccine and COVID-19 specific questions in the initial trial measured *before* financial incentives were offered (see SI Section 1.1.3).

Table S2: Summary statistics

Variable	Mean	SD	Min.	Max.	N
Outcome variables					
2nd dose uptake	0.84	0.36	0.0	1.0	5,019
Shorter wait for 2nd dose	62.60	37.75	20.0	147.0	5,019
2nd dose uptake if 1st dose	0.97	0.17	0.0	1.0	4,358
3rd dose intention	0.87	0.33	0.0	1.0	3,238
3rd dose intention for SEK 100	4.33	1.28	1.0	5.0	3,238
3rd dose intention for SEK 500	4.39	1.23	1.0	5.0	3,238
3rd dose uptake	0.64	0.48	0.0	1.0	2,706
Shorter wait for 3rd dose	4.20	2.59	1.0	8.0	2,706
3rd dose uptake if 2nd dose	0.71	0.46	0.0	1.0	2,463
Flu shot uptake	0.06	0.24	0.0	1.0	3,238
Flu shot intention next season	22.35	27.65	0.0	100.0	3,238
Blood donation	0.11	0.31	0.0	1.0	3,238
Moral and civic responsibility index	-0.00	1.00	-3.5	0.9	3,246
Donation for vaccination	66.66	38.93	0.0	100.0	3,238
Donation for payment for vaccination	34.95	41.18	0.0	100.0	3,238
Perceived safety and efficacy index	-0.00	1.00	-2.9	1.1	3,253
Vaccines generally safe for children	4.75	0.61	1.0	5.0	3,238
Trust in vaccination provision index	-0.00	1.00	-2.8	0.9	3,246
Payment for vaccination is ethical index	-0.00	1.00	-1.3	1.8	3,243
Feels no regret index	-0.00	1.00	-4.8	0.9	3,244
Lower feelings of coercion	4.02	1.30	1.0	5.0	3,238
Treatment					
Financial incentives condition	0.23	0.42	0.0	1.0	5,019
Sociodemographics					
Age	34.62	8.27	18.0	49.0	5,019
Female	0.58	0.49	0.0	1.0	5,019
Single	0.26	0.44	0.0	1.0	5,019

Continued on next page

Table S2 – *Continued from previous page*

Variable	Mean	SD	Min.	Max.	N
Sarbo	0.06	0.23	0.0	1.0	5,019
Couple	0.35	0.48	0.0	1.0	5,019
Married	0.31	0.46	0.0	1.0	5,019
Other Civil Status	0.02	0.15	0.0	1.0	5,019
Has Children	0.53	0.50	0.0	1.0	5,019
Elementary School or Lower	0.02	0.16	0.0	1.0	5,019
High-school	0.31	0.46	0.0	1.0	5,019
Professional Training	0.13	0.33	0.0	1.0	5,019
In College	0.07	0.26	0.0	1.0	5,019
College Degree	0.45	0.50	0.0	1.0	5,019
PhD	0.02	0.13	0.0	1.0	5,019
Employed	0.81	0.39	0.0	1.0	5,019
Unemployed	0.04	0.19	0.0	1.0	5,019
In College	0.11	0.31	0.0	1.0	5,019
Retired	0.01	0.08	0.0	1.0	5,019
Other Professional Situation	0.04	0.20	0.0	1.0	5,019
Mother from Sweden	0.85	0.36	0.0	1.0	5,019
Mother from Rest of Europe	0.10	0.30	0.0	1.0	5,019
Mother from North America	0.00	0.05	0.0	1.0	5,019
Mother from South America	0.01	0.09	0.0	1.0	5,019
Mother from Africa	0.00	0.06	0.0	1.0	5,019
Mother from the Middle-east	0.02	0.13	0.0	1.0	5,019
Mother from the Rest of Asia	0.01	0.12	0.0	1.0	5,019
Mother from Oceania	0.00	0.02	0.0	1.0	5,019
Father from Sweden	0.85	0.36	0.0	1.0	5,019
Father from Rest of Europe	0.10	0.30	0.0	1.0	5,019
Father from North America	0.00	0.06	0.0	1.0	5,019
Father from South America	0.01	0.09	0.0	1.0	5,019
Father from Africa	0.01	0.07	0.0	1.0	5,019

Continued on next page

Table S2 – *Continued from previous page*

Variable	Mean	SD	Min.	Max.	N
Father from the Middle-east	0.02	0.15	0.0	1.0	5,019
Father from the Rest of Asia	0.01	0.11	0.0	1.0	5,019
Father from Oceania	0.00	0.02	0.0	1.0	5,019
Income 0-5000kr	0.03	0.18	0.0	1.0	5,019
Income 5001-10000kr	0.05	0.22	0.0	1.0	5,019
Income 10001-15000kr	0.11	0.31	0.0	1.0	5,019
Income 15001-20000kr	0.10	0.31	0.0	1.0	5,019
Income 20001-25000kr	0.23	0.42	0.0	1.0	5,019
Income 25001-30000kr	0.20	0.40	0.0	1.0	5,019
Income 30001-35000kr	0.13	0.33	0.0	1.0	5,019
Income 35001-40000kr	0.08	0.27	0.0	1.0	5,019
Income 40001-45000kr	0.03	0.18	0.0	1.0	5,019
Income 45001-50000kr	0.01	0.12	0.0	1.0	5,019
Income 50000kr-55000kr	0.01	0.10	0.0	1.0	5,019
Income more than 55000kr	0.01	0.12	0.0	1.0	5,019
COVID-19 related baseline var.					
Ever tested positive for COVID-19	0.23	0.42	0.0	1.0	5,019
COVID-19 vaccines are safe	4.11	1.05	1.0	5.0	5,019
Diseases can be triggered by vaccinations	1.96	1.07	1.0	5.0	5,019
Worried about side-effects	2.61	1.37	1.0	5.0	5,019
Worried about needles	2.20	1.43	1.0	5.0	5,019

2.1.2 Balance and survey participation

Of 5,019 trial participants, 3,238 participated in and finished the first survey. There was no substantial or statistically significant difference across conditions with a 64.1% first survey participation rate in the financial incentives condition (726 in survey vs. 1,131 in trial) and a 64.6% participation rate in the control condition (2,512 in survey vs. 3,888 in trial, see also column 1 in Table S3). Survey completion was very high: 99.4% of participants who started the survey also completed the first survey. Completion rates were equivalent in the financial incentives (99.3%) and the control condition (99.4%).

Regression results confirm equivalent participation and completion rates across conditions. Table S3 first tests whether first survey participation and completion differ by condition. Participants in the financial incentives condition did not participate at a different rate than participants in the control condition. When splitting up the control condition into the trial control condition and the trial no reminders condition (see SI Section 1.1.2), we also do not find any differences in first survey participation. The same holds for completion rates, which again do not differ statistically significantly or in terms of magnitude across conditions.

The previous RCT randomly assigned participants to each condition. Tables S4 to S6 examine whether first survey participation in both treatment conditons is balanced in terms of sociodemographics, personality, economic preferences and COVID-19 related variables (measured before survey participation).

To do so, we regress (OLS) each of these variables on "participated", an indicator for survey participation, and "participated x financial", the interaction of "participated" and an indicator for being in the incentives condition (without any controls). We then report the corresponding coefficients, standard errors, and significance levels. We do not see substantial differences in overall survey participation or condition-specific survey participation across 25 variables. This implies that survey participation is indeed balanced across the treatment conditions. SI Section 2.3 also shows no substantial differences in the coefficients of our main results when using different sets of controls, again confirming random assignment and no differential survey participation.

Tables S7-S10 replicate the same results looking at second survey participation. In total, 2,706 participated in and finished the second survey. There was no substantial or statistically significant difference across conditions with a 53.6% second survey participation rate in the financial incentives condition (606 in survey vs. 1,131 in trial) and a 54.0% participation rate in the control condition (2,100 in survey vs. 3,888 in trial, see also column 1 in Table S7). Survey completion

was very high: 99.0% of participants who started the survey also completed the second survey. Completion rates were equivalent in the financial incentives (98.4%) and the control condition (99.2%).

Regression results confirm similar participation and completion rates across conditions. Table S7 first tests whether second survey participation and completion differ by condition. Participants in the financial incentives condition did not participate at a different rate than participants in the control condition. When splitting up the control condition into the trial control condition and the trial no reminders condition (see SI Section 1.1.2), we also do not find any differences in second survey participation. The same holds for completion rates, which again do not differ statistically significantly or in terms of magnitude across conditions.

The previous RCT randomly assigned participants to each condition. Tables S7 to S10 examine whether second survey participation in both treatment conditons is balanced in terms of sociodemographics, personality, economic preferences and COVID-19 related variables (measured before survey participation).

To do so, we regress (OLS) each of these variables on "participated", an indicator for survey participation, and "participated x financial", the interaction of "participated" and an indicator for being in the incentives condition (without any controls). We then report the corresponding coefficient, standard errors, and significance levels. We find that some subgroups of participants are more likely to participate in the second survey, likely because these individuals are less likely to drop out from the survey panel. However, most importantly, we do not see any treatment differences in the types of participants who participated in the second survey. This implies that survey participation is indeed balanced across the treatment conditions. SI Section 2.3 also shows no substantial differences in the coefficients of our main results when using different sets of controls, again confirming random assignment and no differential survey participation.

Table S3: First survey participation and completion by condition

Dependent Variable	Survey participation		Completed survey	
	(1)	(2)	(3)	(4)
Financial incentives condition	-0.00<br>(0.02)	-0.01<br>(0.02)	-0.00<br>(0.00)	-0.00<br>(0.00)
No reminders condition		-0.01<br>(0.02)		-0.00<br>(0.00)
Observations	5,019	5,019	3,257	3,257

Note: The table shows coefficient estimates from linear regressions of an indicator capturing participation in the survey (only 1 for participants who finished the survey) or finishing the survey (only 1 for participants who finished the survey, 0 for participants who started but did not finish the survey) on an indicator for the financial incentives condition and the no reminders condition. Heteroscedasticity robust standard errors are shown in parentheses.
* $p < 0.10$, ** $p < 0.05$, *** $p < 0.01$

Table S4: First survey participation based on COVID-19 related variables

Dependent Variable	1st dose 30 days	2nd dose uptake	2nd dose if 1st	Short wait 2nd dose	Tested positive	Risk group	Vacc. safe	Vacc. cause disease	Worried side-effects	Worried needles
	(1)	(2)	(3)	(4)	(5)	(6)	(7)	(8)	(9)	(10)
Participated	0.04<br>(0.03)	0.03<br>(0.03)	-0.01<br>(0.04)	-0.04<br>(0.03)	-0.01<br>(0.03)	-0.00<br>(0.03)	-0.00<br>(0.03)	0.01<br>(0.03)	-0.03<br>(0.03)	-0.04<br>(0.03)
Participated x financial inc.	-0.06<br>(0.07)	-0.04<br>(0.07)	0.03<br>(0.07)	0.02<br>(0.07)	0.06<br>(0.07)	0.02<br>(0.07)	-0.02<br>(0.07)	-0.03<br>(0.07)	0.07<br>(0.07)	0.09<br>(0.07)
Financial incentives condition	0.06<br>(0.06)	0.07<br>(0.06)	0.03<br>(0.06)	-0.05<br>(0.06)	-0.01<br>(0.06)	0.01<br>(0.06)	0.05<br>(0.05)	0.00<br>(0.06)	-0.06<br>(0.06)	-0.07<br>(0.06)
Observations	5,019	5,019	4,358	5,019	5,019	5,019	5,019	5,019	5,019	5,019

Note: The table shows coefficient estimates from linear regressions of the standardized characteristic variable on an indicator for the financial incentives condition, an indicator for survey participation, and the interaction of the two. 1st dose 30 days is an indicator variable indicating whether participants got a first dose of a COVID-19 vaccine within 30 days of trial participation. Tested positive is an indicator indicating whether the participant ever tested positive for COVID-19, risk group captures whether a participant is in a COVID-19 risk group, and columns (7) to (10) capture worries about safety and risk of vaccinations based on a 5-point Likert scale. Heteroscedasticity robust standard errors are shown in parentheses.
* $p < 0.10$, ** $p < 0.05$, *** $p < 0.01$

Table S5: First survey participation based on personality and economic preferences

Dependent Variable	Altruism	Risk-affinity	Patience	Reciprocity	Trust	Procrastination	Norm-adherence
	(1)	(2)	(3)	(4)	(5)	(6)	(7)
Participated	-0.04	0.03	0.01	-0.04	0.01	-0.05	0.01
	(0.03)	(0.03)	(0.03)	(0.03)	(0.03)	(0.03)	(0.03)
Participated x financial inc.	0.09	0.07	0.05	0.00	0.07	0.03	-0.03
	(0.07)	(0.07)	(0.07)	(0.07)	(0.07)	(0.07)	(0.07)
Financial incentives condition	-0.09	-0.08	-0.07	-0.06	-0.00	-0.01	0.02
	(0.06)	(0.06)	(0.06)	(0.06)	(0.06)	(0.06)	(0.05)
Observations	5,019	5,019	5,019	5,019	5,019	5,019	5,019

Note: The table shows coefficient estimates from linear regressions of the standardized characteristic variable on an indicator for the financial incentives condition, an indicator for survey participation, and the interaction of the two. The outcome variables capture personality and economic preferences based on a 10-point likert scale. Heteroscedasticity robust standard errors are shown in parentheses.
* $p < 0.10$, ** $p < 0.05$, *** $p < 0.01$

Table S6: First survey participation based on sociodemographics

Dependent Variable	Age	Female	Single	Has childr.	College	Income	Immigr.	Unemployed
	(1)	(2)	(3)	(4)	(5)	(6)	(7)	(8)
Participated	-0.01	-0.05	-0.04	-0.00	0.02	0.08**	0.02	-0.03
	(0.03)	(0.03)	(0.03)	(0.03)	(0.03)	(0.03)	(0.03)	(0.03)
Participated x financial inc.	-0.02	0.10	0.17**	-0.03	-0.04	-0.09	-0.04	0.07
	(0.07)	(0.07)	(0.07)	(0.07)	(0.07)	(0.07)	(0.07)	(0.07)
Financial incentives condition	0.03	-0.01	-0.09*	-0.02	0.02	0.04	0.00	0.02
	(0.06)	(0.06)	(0.05)	(0.06)	(0.06)	(0.06)	(0.06)	(0.06)
Observations	5,019	5,019	5,019	5,019	5,019	5,019	5,019	5,019

Note: The table shows coefficient estimates from linear regressions of the standardized characteristic on an indicator for the financial incentives condition, an indicator for survey participation, and the interaction of the two. Heteroscedasticity robust standard errors are shown in parentheses.
* $p < 0.10$, ** $p < 0.05$, *** $p < 0.01$

Table S7: Second survey participation and completion by condition

Dependent Variable	Survey participation		Completed survey	
	(1)	(2)	(3)	(4)
Financial incentives condition	-0.00	-0.01	-0.01	-0.01
	(0.02)	(0.02)	(0.01)	(0.01)
No reminders condition		-0.02		-0.00
		(0.02)		(0.00)
Observations	5,019	5,019	2,733	2,733

Note: The table shows coefficient estimates from linear regressions of an indicator capturing participation in the survey (only 1 for participants who finished the survey) or finishing the survey (only 1 for participants who finished the survey, 0 for participants who started but did not finish the survey) on an indicator for the financial incentives condition and the no reminders condition. Heteroscedasticity robust standard errors are shown in parentheses.
* $p < 0.10$, ** $p < 0.05$, *** $p < 0.01$

Table S8: Second survey participation based on COVID-19 related variables

Dependent Variable	1st dose 30 days	2nd dose uptake	2nd dose if 1st	Short wait 2nd dose	Tested positive	Risk group	Vacc. safe	Vacc. cause disease	Worried side-effects	Worried needles
	(1)	(2)	(3)	(4)	(5)	(6)	(7)	(8)	(9)	(10)
Participated	0.09***	0.10***	0.05	-0.11***	-0.12***	0.04	-0.10***	-0.00	0.04	-0.03
	(0.03)	(0.03)	(0.04)	(0.03)	(0.03)	(0.03)	(0.03)	(0.03)	(0.03)	(0.03)
Participated x financial inc.	-0.04	-0.06	-0.06	0.09	0.06	0.06	-0.06	0.16**	0.05	0.02
	(0.07)	(0.07)	(0.07)	(0.07)	(0.07)	(0.07)	(0.07)	(0.07)	(0.07)	(0.07)
Financial incentives condition	0.05	0.08	0.08*	-0.08*	-0.01	-0.00	0.07	-0.10**	-0.05	-0.02
	(0.05)	(0.05)	(0.05)	(0.05)	(0.05)	(0.05)	(0.05)	(0.05)	(0.05)	(0.05)
Observations	5,019	5,019	4,358	5,019	5,019	5,019	5,019	5,019	5,019	5,019

Note: The table shows coefficient estimates from linear regressions of the standardized characteristic variable on an indicator for the financial incentives condition, an indicator for survey participation, and the interaction of the two. 1st dose 30 days is an indicator variable indicating whether participants got a first dose of a COVID-19 vaccine within 30 days of trial participation. Tested positive is an indicator indicating whether the participant ever tested positive for COVID-19, risk group captures whether a participant is in a COVID-19 risk group, and columns (7) to (10) capture worries about safety and risk of vaccinations based on a 5-point Likert scale. Heteroscedasticity robust standard errors are shown in parentheses.
* $p < 0.10$, ** $p < 0.05$, *** $p < 0.01$

Table S9: Second survey participation based on personality and economic preferences

Dependent Variable	Altruism	Risk-affinity	Patience	Reciprocity	Trust	Procrastination	Norm-adherence
	(1)	(2)	(3)	(4)	(5)	(6)	(7)
Participated	-0.08**	-0.04	-0.10***	-0.05	-0.04	-0.11***	0.01
	(0.03)	(0.03)	(0.03)	(0.03)	(0.03)	(0.03)	(0.03)
Participated x financial inc.	-0.03	-0.08	-0.02	-0.06	-0.05	0.03	-0.03
	(0.07)	(0.07)	(0.07)	(0.07)	(0.07)	(0.07)	(0.07)
Financial incentives condition	-0.02	0.01	-0.02	-0.03	0.07	-0.00	0.01
	(0.05)	(0.05)	(0.05)	(0.05)	(0.05)	(0.05)	(0.05)
Observations	5,019	5,019	5,019	5,019	5,019	5,019	5,019

Note: The table shows coefficient estimates from linear regressions of the standardized characteristic variable on an indicator for the financial incentives condition, an indicator for survey participation, and the interaction of the two. The outcome variables capture personality and economic preferences based on a 10-point likert scale. Heteroscedasticity robust standard errors are shown in parentheses.
* $p < 0.10$, ** $p < 0.05$, *** $p < 0.01$

Table S10: Second survey participation based on sociodemographics

Dependent Variable	Age	Female	Single	Has childr.	College	Income	Immigr.	Unemployed
	(1)	(2)	(3)	(4)	(5)	(6)	(7)	(8)
Participated	0.17***	0.02	0.01	-0.01	0.06*	0.02	0.01	-0.05
	(0.03)	(0.03)	(0.03)	(0.03)	(0.03)	(0.03)	(0.03)	(0.03)
Participated x financial inc.	0.11	-0.03	-0.02	0.03	0.07	0.06	0.05	0.07
	(0.07)	(0.07)	(0.07)	(0.07)	(0.07)	(0.07)	(0.06)	(0.07)
Financial incentives condition	-0.04	0.07	0.02	-0.05	-0.04	-0.05	-0.05	0.02
	(0.05)	(0.05)	(0.05)	(0.05)	(0.05)	(0.05)	(0.04)	(0.05)
Observations	5,019	5,019	5,019	5,019	5,019	5,019	5,019	5,019

Note: The table shows coefficient estimates from linear regressions of the standardized characteristic on an indicator for the financial incentives condition, an indicator for survey participation, and the interaction of the two. Heteroscedasticity robust standard errors are shown in parentheses.
* $p < 0.10$, ** $p < 0.05$, *** $p < 0.01$

2.1.3 General population sample

Next we compare the demographic composition of our general population sample in both the administrative data and the survey data with the Swedish population (considering the same age restriction of ages 18 to 49). The survey company can only provide a sample that is representative in terms of age, gender, and region. However, we also compare other characteristics to the general population. In addition to age, gender, and region, we also document how our sample compares to the general population in terms of average income, education, and immigration background.

We obtained the Swedish population data from the public registry data reported by Statistics Sweden. While the sample is similar to the general Swedish population in terms of age, region, and income, people with a college education are slightly overrepresented in our sample, immigrants are underrepresented in our sample, and women are slightly overrepresented. In SI Section 2.3.2 we show that results do not change when using sampling weights to reweight our sample.

Table S11: Trial sample, survey sample, and the Swedish population

**Variable**	Trial data		Survey 1 data		Survey 2 data		Sweden Ages 18-49
	N	Mean	N	Mean	N	Mean	Mean
Age	5,019	34.62	3,238	34.58	2,706	35.34	33.71
Female	5,019	0.58	3,238	0.58	2,706	0.58	0.49
Region Stockholm	5,019	0.24	3,238	0.23	2,706	0.23	0.25
Region Östra Mellansverige	5,019	0.16	3,238	0.15	2,706	0.16	0.17
Region Småland med öarna	5,019	0.08	3,238	0.08	2,706	0.08	0.08
Region Sydsverige	5,019	0.15	3,238	0.15	2,706	0.15	0.15
Region Västsverige	5,019	0.22	3,238	0.22	2,706	0.21	0.20
Region Norra Mellansverige	5,019	0.07	3,238	0.07	2,706	0.07	0.07
Region Mellersta Norrland	5,019	0.04	3,238	0.04	2,706	0.04	0.03
Region Övre Norrland	5,019	0.05	3,238	0.06	2,706	0.06	0.05
Share university education	5,019	0.54	3,238	0.54	2,706	0.56	0.44
Average monthly income (SEK)	5,019	24,724	3,238	24,949	2,706	24,884	24,211*
Both parents born in Sweden	5,019	0.90	3,238	0.90	2,706	0.90	0.60†

Note: Comparison of the trial data on age, gender, region, university education, average income, and immigration background with public registry data from Statistics Sweden. We constructed the variables such that they match the Statistics Sweden definition, with two exceptions: * is based on age >19, † is based on both parents were born in Sweden *and* the individual was born in Sweden (due to privacy concerns, we did not elicit whether the individual was born in Sweden in our survey).

2.2 Main results

In this section, we provide the key tables and figures of the analysis of the Swedish study that are not shown in the main text. All the tables are based on exactly the pre-registered specification described in the Methods section of the main text and Supplementary Methods, i.e. OLS regressions with heteroscedasticity-robust standard errors. The controls are the pre-registered controls specified in SI Section 1.

2.2.1 Regression results for main outcome variables

Tables S12 to S17: Treatment effects on main outcomes. These tables provide the effect sizes from our main specification with the pre-registered set of controls (SI Section 1). The tables show that offering financial incentives does not have a statistically significant negative impact on any of the outcomes. All coefficient estimates are small and scattered around 0.

Table S12: Treatment effects on 2nd dose uptake

Dependent Variable	2nd dose uptake	Shorter wait 2nd dose	2nd dose if 1st dose
	(1)	(2)	(3)
Financial incentives condition	0.06*<br>(0.03)	0.05<br>(0.03)	0.05<br>(0.03)
Age x Region FE	yes	yes	yes
Controls	yes	yes	yes
Observations	5,019	5,019	4,358

Note: The table shows coefficient estimates from linear regressions of the standardized outcome on an indicator for the financial incentives condition. Heteroscedasticity robust standard errors are shown in parentheses. All regressions use exactly the pre-registered controls, consisting of sociodemographics controls and age x region fixed effects.
* $p < 0.10$, ** $p < 0.05$, *** $p < 0.01$

Table S13: Treatment effects on 3rd dose uptake

Dependent Variable	3rd dose intention	3rd dose SEK 100	3rd dose SEK 500	3rd dose uptake	Shorter wait 3rd dose	3rd dose if 2nd dose
	(1)	(2)	(3)	(4)	(5)	(6)
Financial incentives condition	-0.03	0.00	-0.01	-0.01	0.03	-0.02
	(0.04)	(0.04)	(0.04)	(0.05)	(0.05)	(0.05)
Age x Region FE	yes	yes	yes	yes	yes	yes
Controls	yes	yes	yes	yes	yes	yes
Observations	3,238	3,238	3,238	2,706	2,706	2,463

Note: The table shows coefficient estimates from linear regressions of the standardized outcome on an indicator for the financial incentives condition. Heteroscedasticity robust standard errors are shown in parentheses. All regressions use exactly the pre-registered controls, consisting of sociodemographics controls and age x region fixed effects.
* $p < 0.10$, ** $p < 0.05$, *** $p < 0.01$

Table S14: Treatment effects on other health behaviors

Dependent Variable	Flu shot uptake	Flu shot intention next season	Blood donation
	(1)	(2)	(3)
Financial incentives condition	0.00	0.01	-0.02
	(0.04)	(0.04)	(0.04)
Age x Region FE	yes	yes	yes
Controls	yes	yes	yes
Observations	3,238	3,238	3,238

Note: The table shows coefficient estimates from linear regressions of the standardized outcome on an indicator for the financial incentives condition. Heteroscedasticity robust standard errors are shown in parentheses. All regressions use exactly the pre-registered controls, consisting of sociodemographics controls and age x region fixed effects.
* $p < 0.10$, ** $p < 0.05$, *** $p < 0.01$

Table S15: Treatment effects on morals and civic responsibility

Dependent Variable	Moral and civic. resp.	Donation vaccination	Donation payment for vacc.
	(1)	(2)	(3)
Financial incentives condition	0.00<br>(0.04)	-0.02<br>(0.04)	0.07<br>(0.04)
Age x Region FE	yes	yes	yes
Controls	yes	yes	yes
Observations	3,238	3,238	3,238

Note: The table shows coefficient estimates from linear regressions of the standardized outcome on an indicator for the financial incentives condition. Heteroscedasticity robust standard errors are shown in parentheses. All regressions use exactly the pre-registered controls, consisting of sociodemographics controls and age x region fixed effects.
* $p < 0.10$, ** $p < 0.05$, *** $p < 0.01$

Table S16: Treatment effects on perceived safety, efficacy, and trust

Dependent Variable	Safety and efficacy	Vaccines safe for children	Trust in vacc. provision
	(1)	(2)	(3)
Financial incentives condition	-0.03<br>(0.04)	-0.03<br>(0.04)	0.00<br>(0.04)
Age x Region FE	yes	yes	yes
Controls	yes	yes	yes
Observations	3,238	3,238	3,238

Note: The table shows coefficient estimates from linear regressions of the standardized outcome on an indicator for the financial incentives condition. Heteroscedasticity robust standard errors are shown in parentheses. All regressions use exactly the pre-registered controls, consisting of sociodemographics controls and age x region fixed effects.
* $p < 0.10$, ** $p < 0.05$, *** $p < 0.01$

Table S17: Treatment effects on other concerns

Dependent Variable	Payments for vacc. ethical	Feels no regret	Lower feelings of coercion
	(1)	(2)	(3)
Financial incentives condition	0.03	-0.04	-0.01
	(0.04)	(0.04)	(0.04)
Age x Region FE	yes	yes	yes
Controls	yes	yes	yes
Observations	3,238	3,238	3,238

Note: The table shows coefficient estimates from linear regressions of the standardized outcome on an indicator for the financial incentives condition. Heteroscedasticity robust standard errors are shown in parentheses. All regressions use exactly the pre-registered controls, consisting of sociodemographics controls and age x region fixed effects.
* $p < 0.10$, ** $p < 0.05$, *** $p < 0.01$

2.2.2 Treatment effects on second dose uptake only using data from survey participants

Figures S1 and S2: Treatment effects for 2nd dose uptake using only data on uptake from participants who finished the surveys. Figures S1 and S2 show that the results are equivalent when we examine the impacts on 2nd dose only for first and second survey participants (respectively), again confirming the absence of substantial impacts and no differential survey participation by condition.

Figure S1: Treatment effects on 2nd dose uptake for only participants first survey

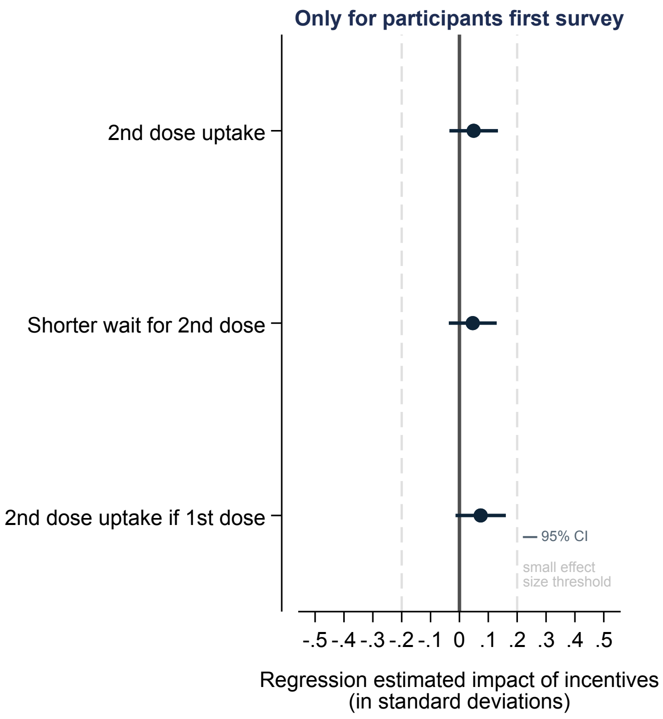

Figure S2: Treatment effects on 2nd dose uptake for only participants second survey

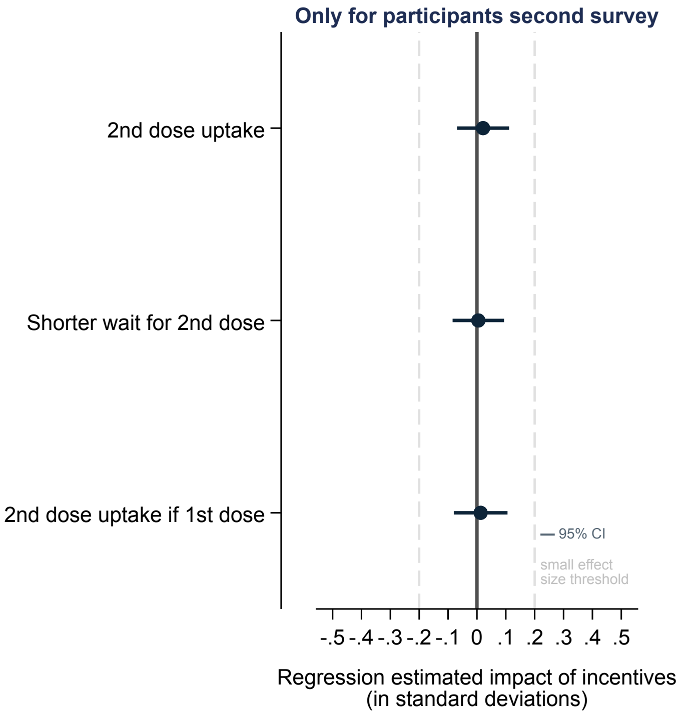

2.2.3 Timing of second dose uptake

Figure S3: Proportion of participants who got the 2nd dose of a COVID-19 vaccine per day after the previous trial. This figure plots the Kaplan-Meier curves of the proportion of participants who got the 2nd dose of a COVID-19 vaccine by a given day after participation in the previous trial.

Figure S3: Proportion of participants who got the 2nd dose of a COVID-19 vaccine per day after the previous trial

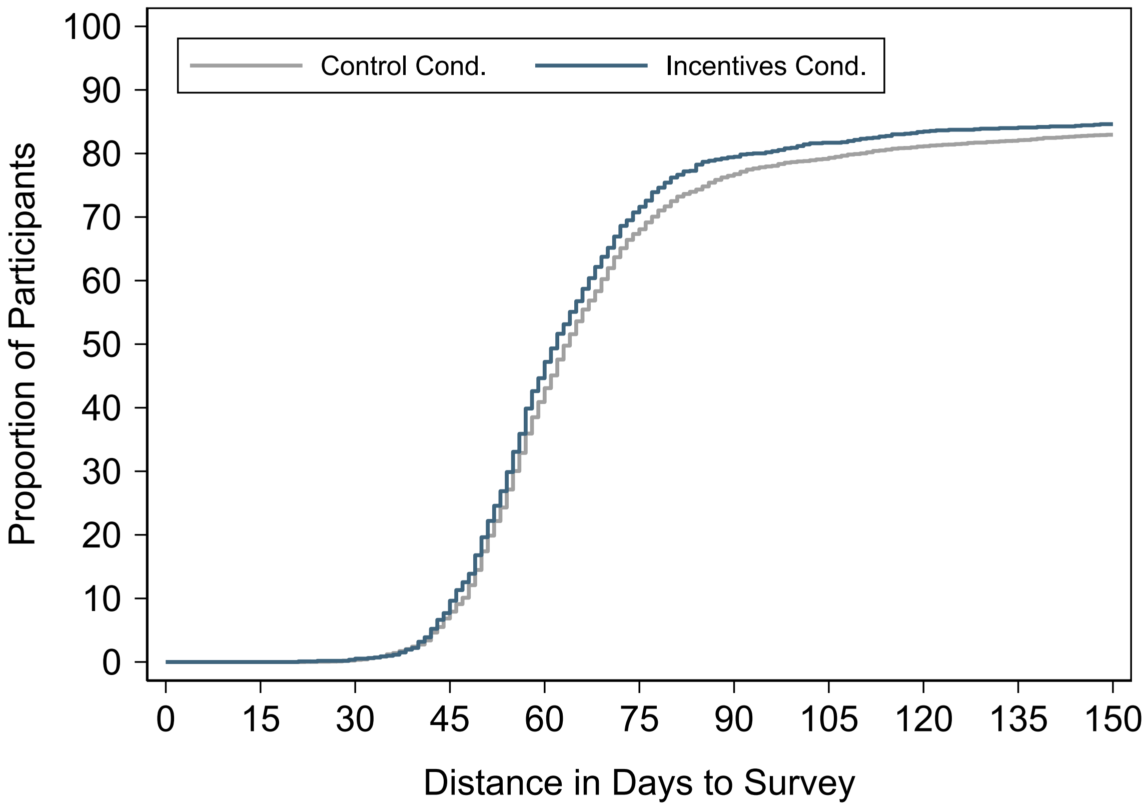

2.2.4 Equivalence testing

In this section we use equivalence tests to study whether we can reject meaningful negative impacts of financial incentives (*54*, *55*). For each outcome, we test the null hypothesis that the estimated effect size is more negative than a small negative effect benchmark.

Equivalence tests require researchers to specify the smallest effect size of interest (SESOI) as a benchmark. In cases where previous research firmly established a plausible range of effect sizes, researchers can often rely on these previously estimated effect sizes to specify the SESOI (*55*). Given the limited field evidence on unintended consequences of financial incentives, however, it is not possible to select a SESOI based on previously reported effect sizes. There are also no precise theoretical predictions on the size of the effects (there are only predictions about the sign of the effects). To specify the SESOI, we therefore first rely on the standard, conservative definition of a small effect size of -0.2 standard deviations based on Cohen's d (*76*). Accordingly, the null hypothesis (H0) we test against is that the effect size is more negative than -0.2 standard deviations, and the alternative hypothesis (H1) is that the effect size is less negative than -0.2 standard deviations. In practice, this means that we do a one-sided t-test of each estimated effect size being less negative than the SESOI of -0.2 (*55*).

Table S18 provides the results of these equivalence tests. We can clearly reject impacts more negative than -0.2 standard deviations (H0) for each outcome. All tests are highly statistically significant, with the largest p-value among all outcomes being 0.0001. Hence, for each outcome, we can rule out even a small effect of -0.2 standard deviations. We conclude that there is strong evidence for the absence of even small negative effects.

An alternative approach to using the standard definition of a small effect based on Cohen's d is to specify the SESOI as the minimum effect size that the study design has power to detect (*55*). As stated in our pre-registration plan, our analysis has 80% power to detect effects of up to -0.12 standard deviations at the 5% level (see SI section 1.1). In a second set of tests, we therefore specify the SESOI as -0.12.

The rightmost column in Table S18 shows the results of equivalence testing based on the null hypothesis that the effect size is more negative than the trivially small effect of -0.12 standard deviations. Once again, the tests reject the null hypothesis (H0) of negative effects larger than -0.12 standard deviations for each outcome: All p-values indicate statistical significance at least at the 5% level that we pre-registered. Again, we conclude that our data provides strong evidence for the absence of even very small negative effects.

Table S18: Equivalence testing

Outcome	P-values equivalence testing	
	SESOI = -0.2	SESOI = -0.12
COVID-19 vaccination:		
2nd dose uptake	0.0000	0.0000
Shorter wait for 2nd dose	0.0000	0.0000
2nd dose uptake if 1st dose	0.0000	0.0000
3rd dose intention	0.0000	0.0171
3rd dose intention for SEK 100	0.0000	0.0026
3rd dose intention for SEK 500	0.0000	0.0050
3rd dose uptake	0.0000	0.0067
Shorter wait for 3rd dose	0.0000	0.0006
3rd dose uptake if 2nd dose	0.0001	0.0161
Other health behaviors:		
Flu shot uptake	0.0000	0.0021
Flu shot intention next season	0.0000	0.0013
Blood donation	0.0000	0.0108
Moral and civic responsibility:		
Moral and civic responsibility index	0.0000	0.0027
Donation for vaccination	0.0000	0.0115
Donation for payment for vaccination	0.0000	0.0000
Perceived safety and efficacy:		
Perceived safety and efficacy index	0.0001	0.0201
Trust in vaccination provision index	0.0000	0.0024
Vaccines generally safe for children	0.0000	0.0170
Other concerns:		
Payment for vaccination is ethical index	0.0000	0.0004
Feels no regret index	0.0001	0.0323
Lower feelings of coercion	0.0000	0.0059

2.3 Robustness checks

This section presents robustness checks for the Swedish study. We first show that our results are robust to including different subsets of the pre-registered controls. While our sample is largely representative of the Swedish population in terms of age, income, and region, we have a slight misrepresentation in terms of gender, education, and immigration status. Second, we show that the results remain equivalent when we perform our main analysis reweighting the sample to make it representative of the Swedish population. Finally, we show that our results remain consistent when we also include participants for whom we observe certain outcome variables, but who did not finish the survey.

2.3.1 Different sets of controls

Figures S4 to S9: Treatment effects including different sets of controls. We pre-registered that for our main analysis we would control for gender, age, region, interactions between age and region, being in an at-risk group for COVID-19, civil status, having children in the household, employment status, education, parents' place of birth, and income. The following figures show that our results are robust to including different sets of control variables.

Figure S4: Different controls: no controls

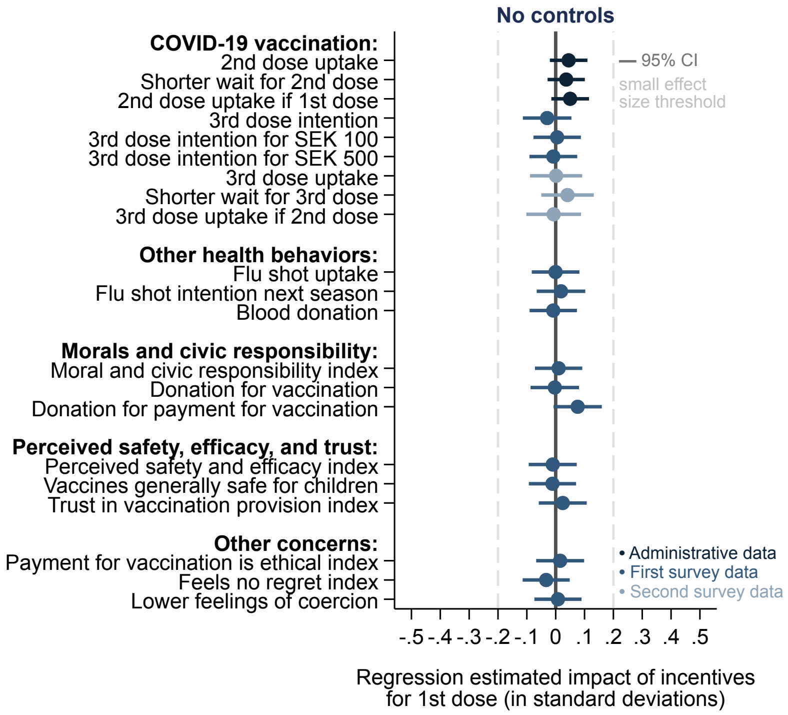

Figure S5: Different controls: controlling for region x age fixed effects (FE)

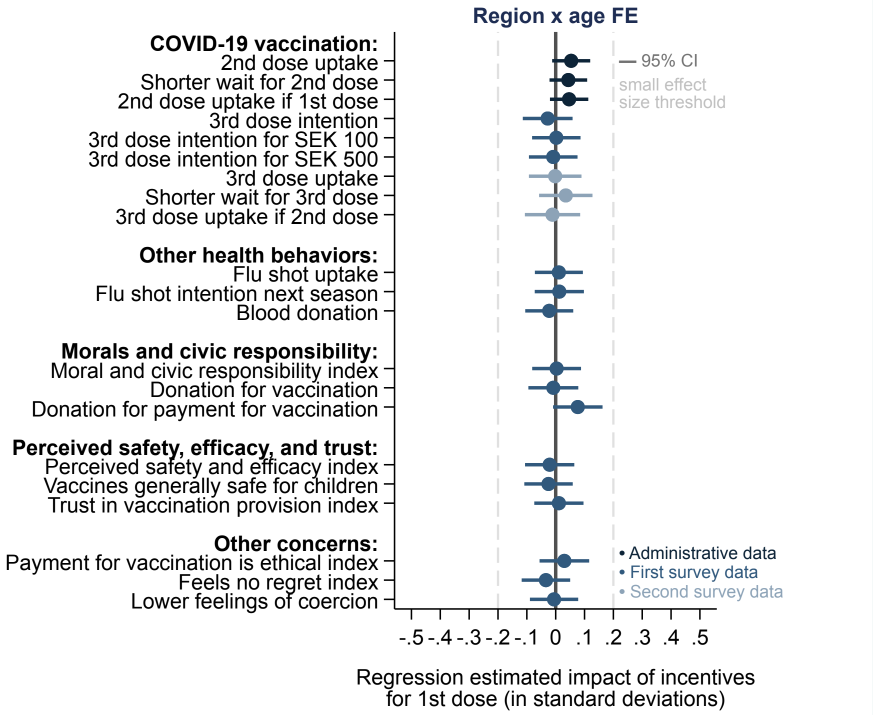

Figure S6: Different controls: controlling for region x age fixed effects and gender

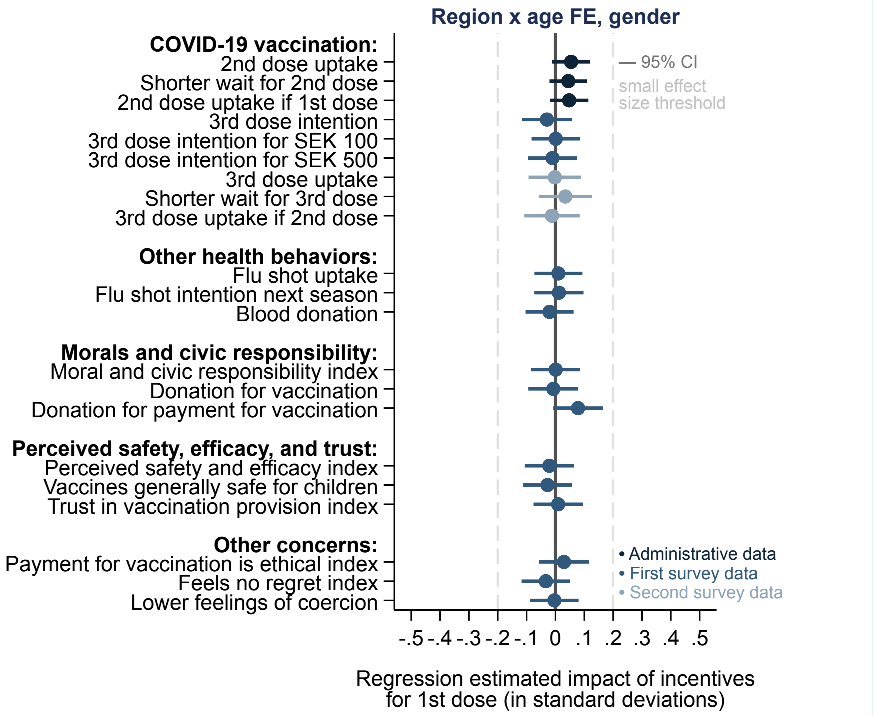

Figure S7: Different controls: controlling for region x age FE, gender, and sociodemographics (education groups, income groups, occupation groups, parental origin)

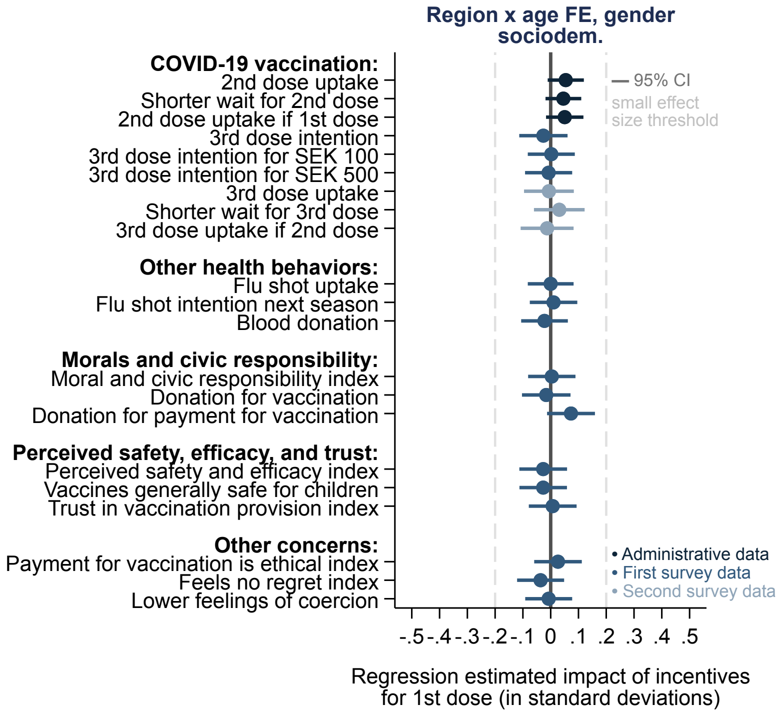

Figure S8: Different controls: controlling for region x age FE, sociodemographics, and family characteristics (has children, marital status)

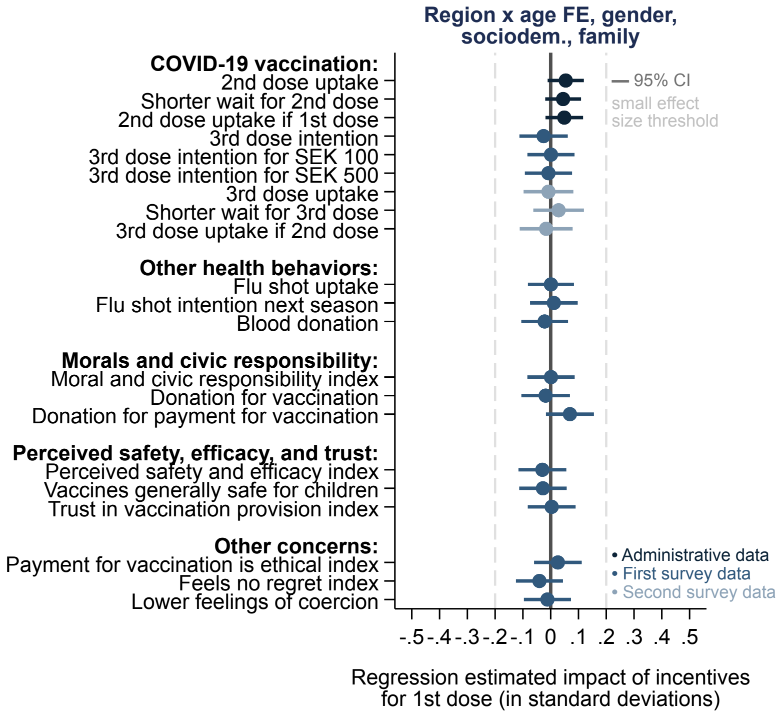

Figure S9: Main specification: controlling for region x age FE, sociodemographics, family characteristics, and COVID-19 risk group status

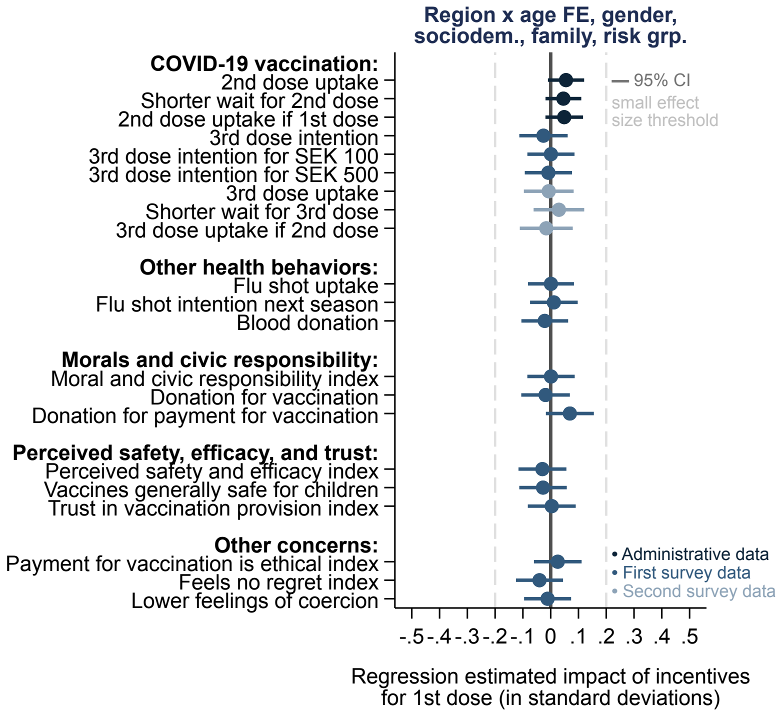

2.3.2 Sample weights

Figure S10: Treatment effects with sample weights. Our sample is representative of the Swedish population (aged 18-49) in terms of age, region, and income. However, we have a slight overrepresentation of people with college education, an underrepresentation of people whose parents or themselves were born abroad, and a slight overrepresentation of women (as discussed in SI Section 2.1.3).

In this figure, we show that results do not change when using sampling weights for adjustment. We replicate our main analysis (see SI Section 2.2.1), but reweight individuals with college education, immigration background, and women in such a way that the estimates are based on a sample which has the same characteristics as the general population in Sweden (we used raking adjusted weights based on the variable definitions according to Statistics Sweden and as discussed in SI Section 2.1.3 using the characteristics of all participants for whom we have administrative data).

Figure S10: Treatment effects with sample weights

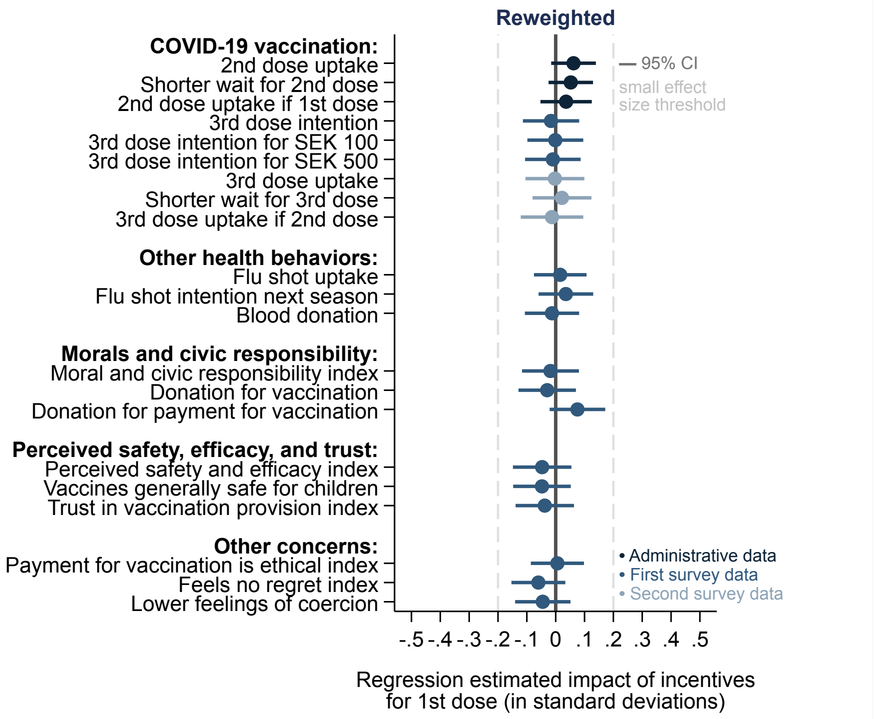

2.3.3 Including participants who did not finish the survey

Figure S11: Treatment effects including participants who did not finish the survey. The survey completion rate were over 99% with no differential survey completion rates across conditions (see SI Section 2.1.2). In the main specification we exclude observations from all participants who did not finish the survey. In this figure, we show that including all participants, including those who did not finish the survey, does not affect our results.

Figure S11: Treatment effects including participants who did not finish the survey

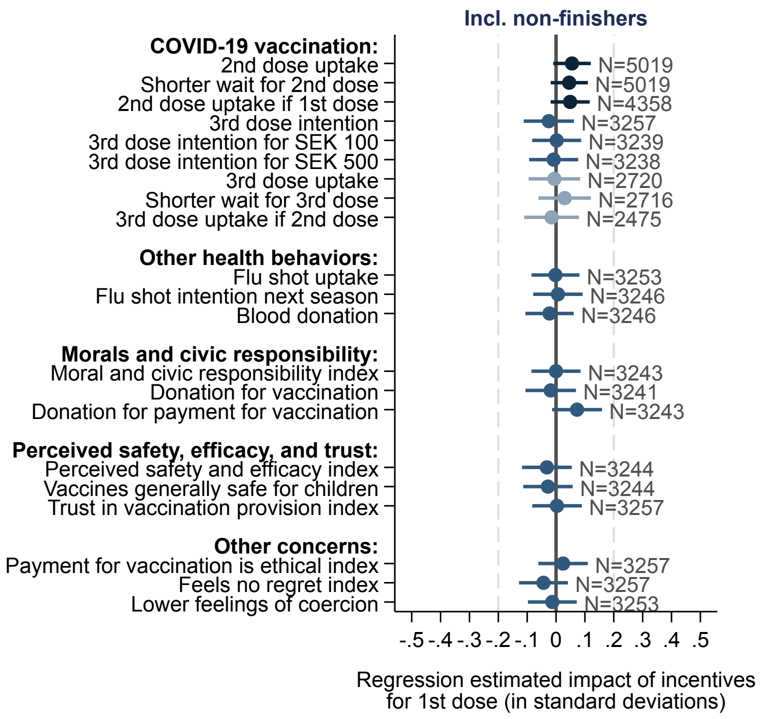

2.4 Additional results

This section presents additional results from the Swedish study. We first present the distributions of the main outcome variables for participants in the control and financial incentives conditions. Second, we show null treatment effects on the dispersion (rather than the average) of each of the outcome variables. Third, we study in detail whether there were any heterogeneous treatment effects: this is, whether incentives lead to unintended consequences for some subgroup of individuals. We find no consistent treatment effects for any of the subgroups. Fourth, we show null treatment effects for each of the items of the indices that we use as outcome variables. Finally, we similarly show the treatment effects for the rest of our secondary and exploratory outcome variables.

2.4.1 Distribution of raw outcomes depending on condition

Figures S12 to S15: Distribution of raw outcomes depending on condition. The following figures show the distributions of the untransformed outcomes, except for indices which are standardized as described in the Methods section of the paper. The figures indicate that the variance of the raw outcomes did not increase, nor were the means affected negatively by the financial incentives condition. See also SI Section 2.4.2 for an analysis of the dispersion of the outcome variables.

Figure S12: Distribution of raw outcomes depending on condition, Part 1

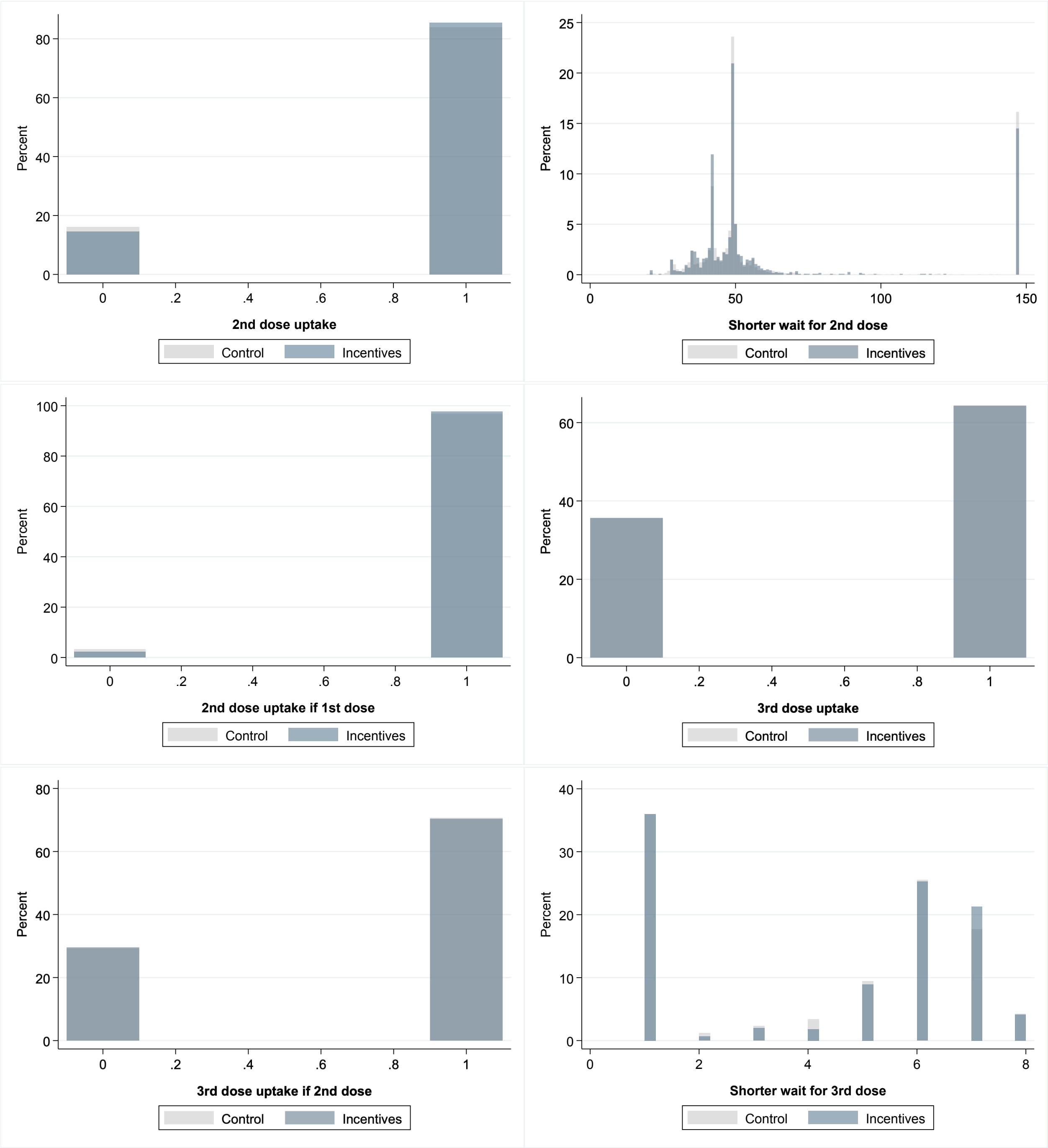

Figure S13: Distribution of raw outcomes depending on condition, Part 2

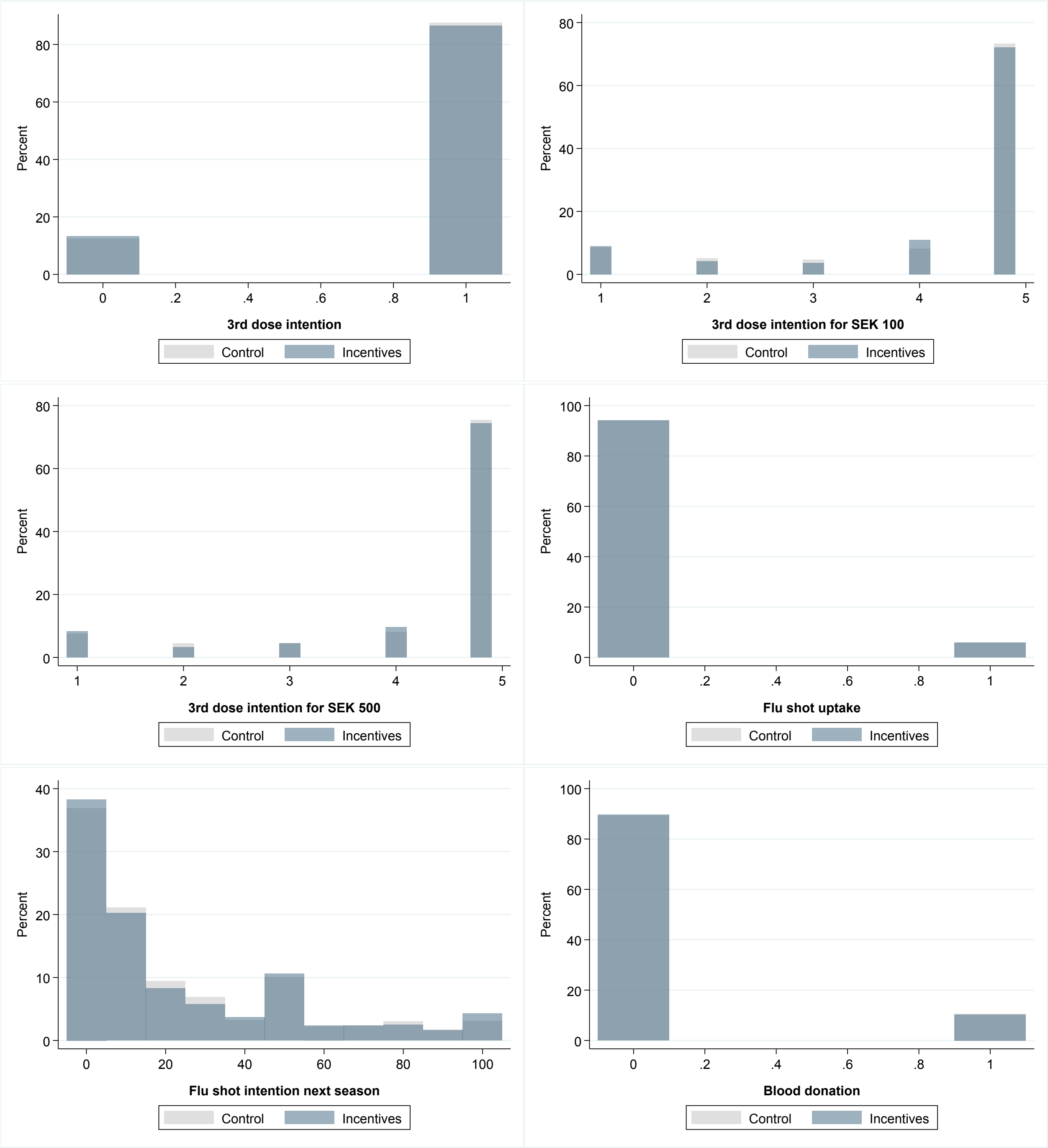

Figure S14: Distribution of raw outcomes depending on condition, Part 3

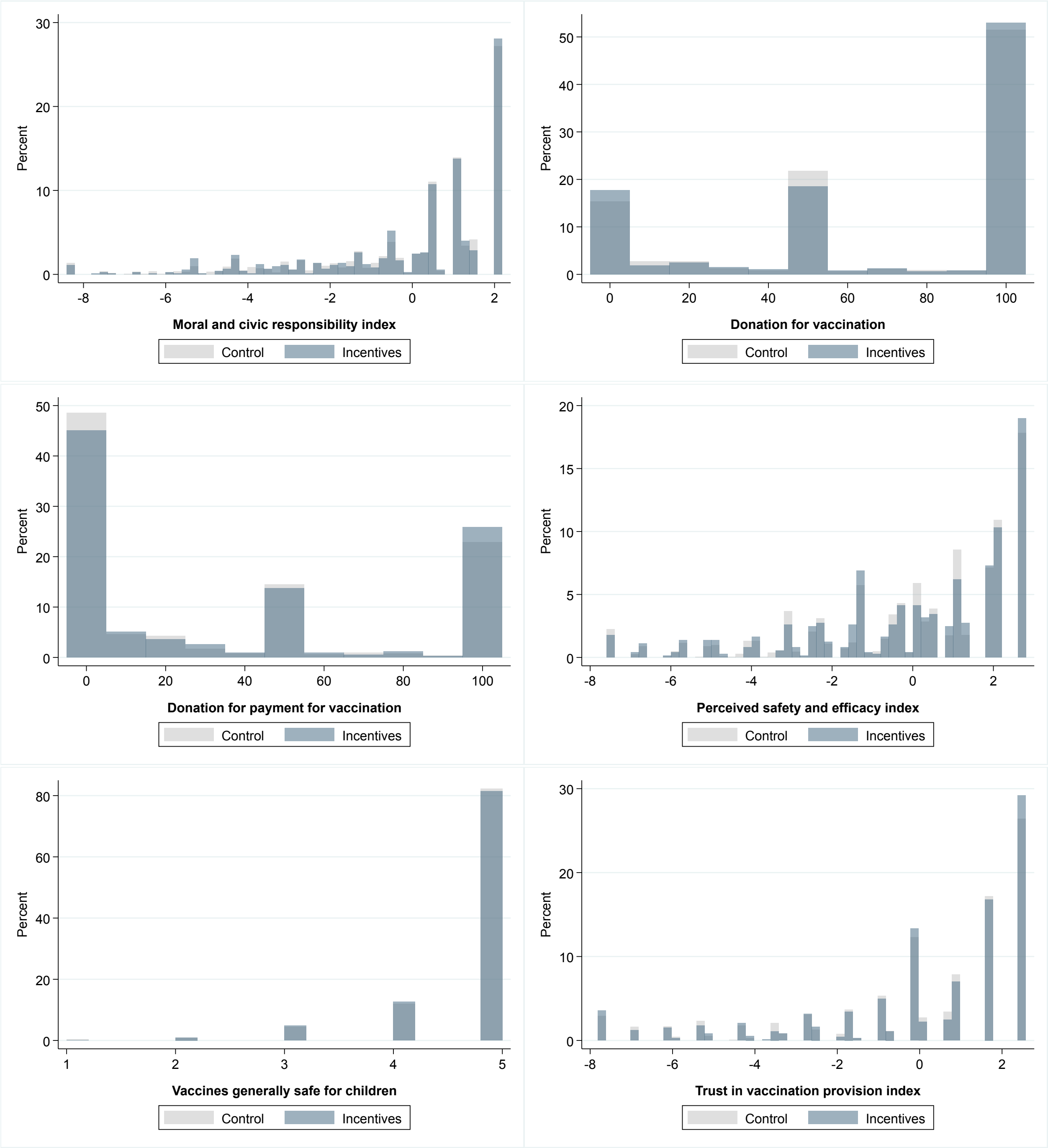

Figure S15: Distribution of raw outcomes depending on condition, Part 4

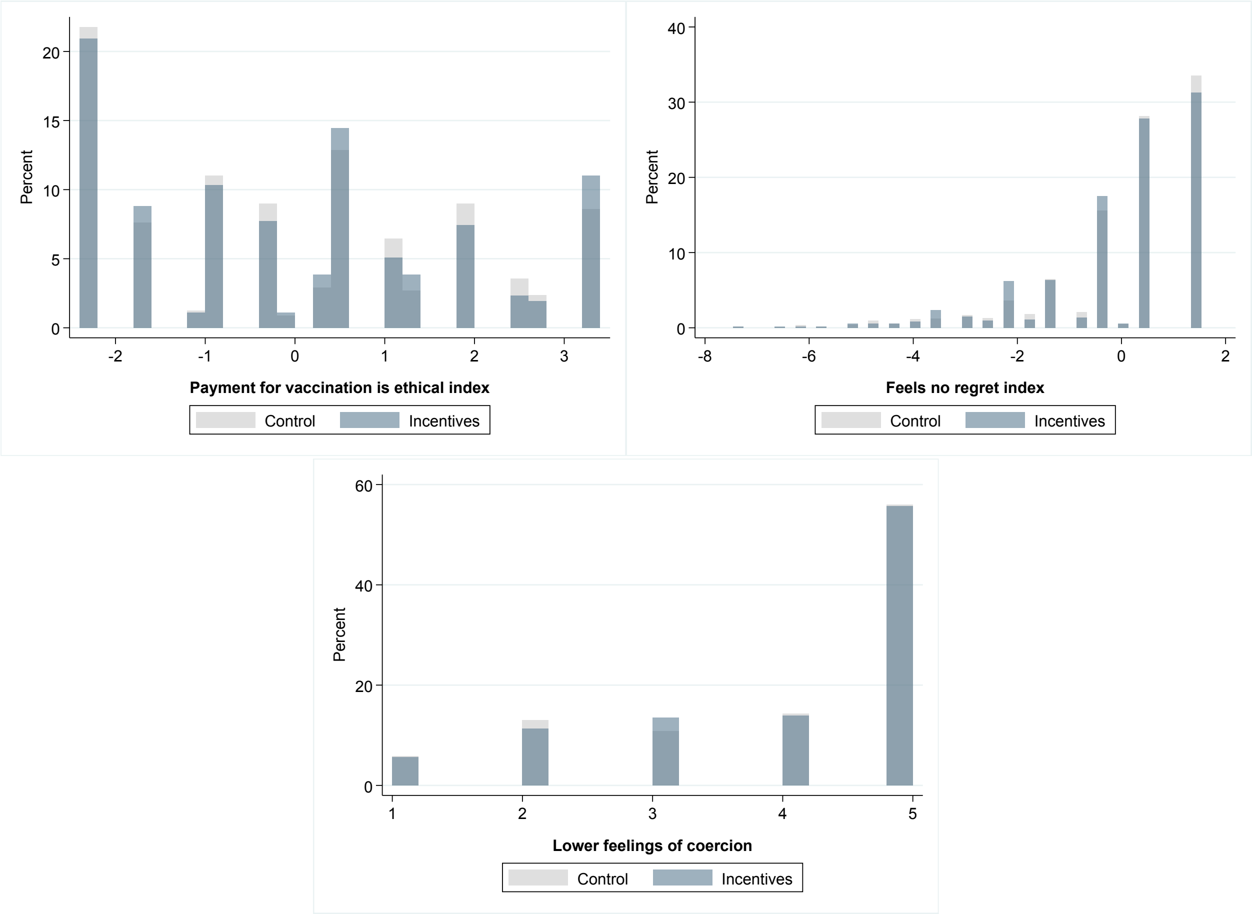

2.4.2 Treatment effects on dispersion of outcome variables

Figure S16: Treatment effects on dispersion of outcome variables. The following figure shows the impact of being in the financial incentives condition on the dispersion of the outcome variables. We define the dispersion as the absolute deviation of the participant outcome to the average outcome. Hence, the dispersion variable captures the size of the absolute deviations from means across the conditions, allowing us to test whether the financial incentives led to more variation in some outcomes. We then run OLS regressions with the absolute deviations as the dependent variable on an indicator for the financial incentives condition and controls. The results show that not only the means of the outcomes were very similar across conditions, but also the variation in the outcomes.

Figure S16: Treatment effects on dispersion of outcome variables

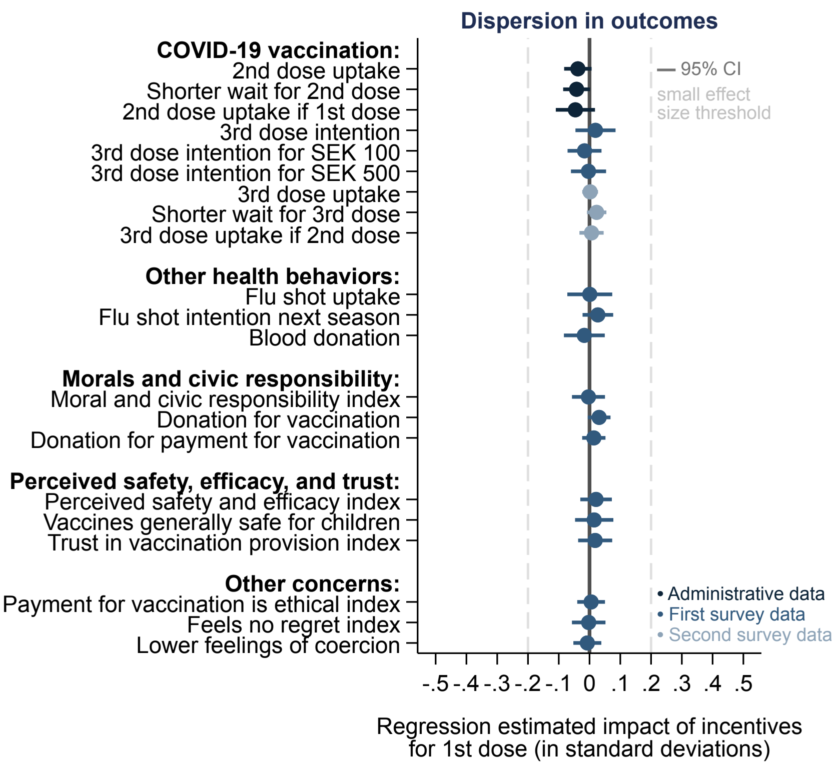

2.4.3 Treatment effects based on participants' characteristics

This section explores potential heterogeneous impacts of the financial incentives condition on the main outcomes based on participants' characteristics in the Swedish study. We first do heterogeneity analyses based on participants' vaccination attitudes. Considering vaccination attitudes is important because the theoretical concerns in the literature on unintended consequences are particularly relevant for individuals with positive vaccination attitudes; these are the individuals who are prosocially and intrinsically motivated, and who believe that vaccines are safe and effective. Financial incentives could erode these individuals' prosocial and intrinsic motivation and worsen risk and safety perceptions. On the other hand, offering incentives might make the hesitant even more skeptical of vaccination. Second, we do heterogeneity analyses based on different sociodemographics. Importantly, all characteristics were measured before assignment to the control or financial incentives condition.

Summary of results: We do not find any robust heterogeneities across 567 coefficient estimates. This holds true whether we use a within or between participant specifications.

Despite the very large number of coefficients tested, no coefficient is statistically significant at the 1% level. Only 9 coefficients are statistically significant at the 5% level (7 positive, 2 negative). If there were no heterogeneous impacts, by chance alone we would expect to see some of the 567 coefficients to be statistically significant at the 5% level. Hence, while some coefficients are statistically significant, there is no indication that there were consistent heterogeneities based on background characteristics.

Variable definitions: We consider different measures of vaccine hesitancy. All measures are based on the following COVID-19 related variables measured in the previous trial before the assignment to the treatment conditions:

- **Vaccine safety**: We asked participants whether they think that, in general, COVID-19 vaccines are safe (scale from 1 to 5).
- **Vaccine triggers diseases**: We asked participants whether they think diseases like autism, multiple sclerosis, and diabetes can be triggered through vaccination (scale from 1 to 5).
- **Worries about side-effects**: We asked participants whether they are worried about the side effects from COVID-19 vaccines (scale from 1 to 5).

- **Worries of needles**: We asked participants whether they are afraid of the needles used for vaccination (scale from 1 to 5).

For our main specification, we aggregate these four measures. The weight of the measures is determined by a regression of an indicator of whether a participant took a first dose of a COVID-19 vaccine within 30 days on all four measures (and all pre-registered controls). Specifically, we predict each participants likelihood of getting a first dose of a vaccine based on the estimated model and use the resulting likelihood to categorize participants as more hesitant and not hesitant.

Vaccine hesitancy (quintiles). First, we categorize participants' likelihood by quintiles. Participants in the first quintile have negative vaccination attitudes while participants in the fifth quintile have very positive attitudes: 35%, 66%, 82%, 86% and 93% of participants in the first, second, third, fourth and fifth quintile, respectively, took the first dose within 30 days. Note that the vaccination rates in the more hesitant groups here are still higher than in other contexts (as *56* and *57*).

Vaccine hesitancy (median split). Second, we categorize the likelihood by the median. Participants who have a predicted likelihood lower than 76% to vaccinate are classified as more hesitant, and participants who have a predicted likelihood higher than 76% are classified as less hesitant. Accordingly, more hesitant participants are participants who, based on sociodemographics and COVID-19 vaccination attitudes, were less likely to vaccinate. Indeed, while 89% of the less hesitant participants took a first dose of a COVID-19 vaccine within 30 day, only 56% of the more hesitant participants took the first dose within 30 days.

Third, we also provide results for the four COVID-19 related variables separately based on median splits. That is, we take the raw measures of each of the four variables described above and then split them by the median to create groups with different vaccine hesitancy.

Fourth, we provide results on heterogeneities across sociodemographics. To this end, we split the continuous variables based on median values, which are 34 for age and SEK 22,500 for income.

Specification to assess differences between participant groups: We use an OLS model where we interact an indicator for the financial incentives condition with indicators capturing in which quantile a participant is (e.g., above or below the median). That is, we regress each outcome on indicators capturing in which quantile a participant is for a given characteristic, and interactions between the financial incentives condition and the indicators for the quantiles. For example, when we categorize participants according to median splits, we use the following specification:

$$Outcome_i = b_0 + b_1 * 1(Above\_median)_i + b_2 * 1(Incentives)_i * 1(Above\_median)_i$$

$$+ b_3 * 1(Incentives)_i * 1(Below\_median)_i + b_4 * X_i + e_i$$

where $1(Incentives)_i$ has a value of 1 if participant i is in the financial incentives condition and a value of 0 otherwise, $1(Above\_median)_i$ has a value of 1 if the participant is above the median of a certain socio-demographic characteristic or COVID-19 vaccination attitude, and $1(Below\_median)_i$ has a value of 1 if the participant is below the corresponding median.

The coefficients for the interactions between the financial incentives condition and the indicator variables (e.g., b_2 and b_3 in the example above) give the *total* impact of offering financial incentives on the participant group with below median values and separately for the participant group with above median values. That is, the interaction effect can be interpreted as the total effect of being in the incentives condition given the participant's characteristic.

X_i is the vector of the pre-registered control variables and e_i is an individual specific error robust to heteroscedasticity. We indicate the interaction for participants with above median values of a characteristic with "high" (e.g., high age is above median age) and for participants with below median values of a characteristic as "low" (e.g., low age is below median age).

Specifications to assess differential changes within participants: In additional analyses, we use the fact that for Vaccine safety and Worries about side-effects (see definition above) we have the same measure twice: Once from the previous RCT before treatment conditions were assigned and once after treatment conditions were assigned as measured in our main (first) survey. This allows us to examine two aspects: i) we can check whether individuals in the financial incentives condition relative to the control condition showed different changes in safety perceptions and worries about side-effects and ii) we can check whether these within-participants changes varied across participants who were more worried about side-effects or safety and participants who were less worried about side-effects or safety.

To examine i) we run the following OLS regressions:

$$Outcome_{i,survey} - Outcome_{i,trial} = b_0 + b_1 * 1(Incentives)_i + b_4 * X_i + e_i$$

where the dependent variable captures within-participant changes between the trial and the survey, and 1(Incentives) is an indicator variable which is 1 if the participant was in the financial incentives condition. X_i is the vector of the pre-registered control variables.

To examine ii) we run the following OLS regressions:

$$Outcome_{i,survey} - Outcome_{i,trial} = b_0 + b_1 * 1(Incentives)_i +$$
$$b_2 * 1(Incentives)_i * Outcome_{i,trial} + b_4 * X_i + e_i$$

where the dependent variable captures within-participant changes between the trial and the survey, and 1(Incentives) is an indicator variable which is 1 if the participant was in the financial incentives condition. In contrast to the first specification, here we interact whether the participant was in the financial incentives condition with their baseline level of the either the variable capturing the safety perceptions or the worries about side-effects. The coefficient on the interaction term indicates whether participants with different levels of vaccine hesitancy before being offered financial incentives changed their view differently. X_i is the vector of the pre-registered control variables.

Note that the heterogeneous treatment effects, when added up, may slightly deviate from the average treatment effects reported in the main text. This is because, when we estimate the heterogeneous treatment effects, by construction we have to add additional variables as controls (the ones for which we check whether there are heterogeneous treatment effects). Hence, in some cases the set of controls differs between the main analysis and the analysis of heterogeneous treatment effects which leads to minor differences when computing the average treatment based on the heterogeneity analyses.

Tables S19 to S26: Heterogeneous treatment effects based on vaccine hesitancy and sociodemographics. Using the first specification described in the introduction of this section, these tables report the heterogeneous treatment effects for each condition based on participants' vaccine hesitancy and sociodemographics. As outlined in detail above, there is no indication of systematic, large, negative impacts of offering financial incentives when exploring these heterogeneities.

Table S19: Heterogeneous treatment effects based on vaccine hesitancy on 2nd dose uptake

Measure:	Outcomes		
	2nd dose uptake	Shorter wait 2dose	2nd dose if 1st
Vaccine hesitancy (quintiles):			
1st quintile x incentives	0.22**	0.18*	0.24*
	(0.10)	(0.10)	(0.13)
2nd quintile x incentives	0.01	0.01	0.13
	(0.08)	(0.08)	(0.08)
3rd quintile x incentives	0.01	-0.00	-0.07
	(0.06)	(0.06)	(0.08)
4th quintile x incentives	0.04	0.06	0.01
	(0.05)	(0.05)	(0.05)
5th quintile x incentives	-0.04	-0.05	-0.00
	(0.05)	(0.05)	(0.05)
Vaccine hesitancy (median split):			
More hesitant x incentives	0.13**	0.12**	0.14**
	(0.06)	(0.05)	(0.06)
Less hesitant x incentives	-0.03	-0.03	-0.03
	(0.04)	(0.04)	(0.04)
Vaccine safety:			
Vaccines are safe x incentives	0.02	0.03	0.04
	(0.04)	(0.04)	(0.03)
Vaccines are not safe x incentives	0.07	0.05	0.05
	(0.05)	(0.05)	(0.06)
Vaccine triggers diseases:			
Vaccines trigger diseases x incentives	0.10	0.08	0.10
	(0.07)	(0.06)	(0.07)
Vaccines do not trigger diseases x incentives	0.03	0.03	0.03
	(0.04)	(0.04)	(0.04)
Worries about side-effects:			
Worried about side-effect x incentives	0.10*	0.09	0.10
	(0.06)	(0.06)	(0.07)
Not worried about side-effect x incentives	0.01	0.01	0.01
	(0.04)	(0.04)	(0.03)
Worries of needles:			
Worried of needles x incentives	0.07	0.06	0.10*
	(0.05)	(0.05)	(0.05)
Not worried of needles x incentives	0.04	0.03	0.00
	(0.05)	(0.05)	(0.05)
Region x Age FE	yes	yes	yes
Controls	yes	yes	yes

Note: The table shows coefficient estimates from linear regressions of the standardized outcome on an indicator for the financial incentives condition interacted with indicators capturing in which quantile a participant's characteristic is (e.g., above or below the median). The corresponding coefficients indicate the total effect for each subgroup. Heteroscedasticity robust standard errors are shown in parentheses. All regressions use exactly the pre-registered controls, consisting of sociodemographics controls and age x region fixed effects.
* $p < 0.10$, ** $p < 0.05$, *** $p < 0.01$

Table S20: Heterogeneous treatment effects based on vaccine hesitancy on 3rd dose uptake

Measure:	Outcomes					
	3rd dose intention	3rd dose 100 SEK	3rd dose 500 SEK	3rd dose	Short wait 3rd	3rd dose if 2nd
Vaccine hesitancy (quintiles):						
1st quintile x incentives	0.07	0.10	0.07	0.11	0.13	0.02
	(0.10)	(0.10)	(0.10)	(0.10)	(0.10)	(0.13)
2nd quintile x incentives	-0.15	-0.20*	-0.18*	-0.04	-0.01	0.01
	(0.11)	(0.11)	(0.11)	(0.10)	(0.10)	(0.11)
3rd quintile x incentives	0.05	0.09	0.08	0.04	0.08	0.06
	(0.09)	(0.09)	(0.09)	(0.09)	(0.09)	(0.10)
4th quintile x incentives	-0.06	0.00	-0.01	-0.10	-0.04	-0.12
	(0.10)	(0.10)	(0.10)	(0.10)	(0.11)	(0.11)
5th quintile x incentives	-0.06	-0.02	-0.03	0.02	0.06	0.01
	(0.09)	(0.09)	(0.09)	(0.08)	(0.09)	(0.09)
Vaccine hesitancy (median split):						
More hesitant x incentives	-0.00	0.00	-0.02	0.04	0.06	0.02
	(0.06)	(0.06)	(0.06)	(0.06)	(0.06)	(0.07)
Less hesitant x incentives	-0.05	-0.00	-0.00	-0.04	0.02	-0.03
	(0.06)	(0.06)	(0.06)	(0.06)	(0.06)	(0.06)
Vaccine safety:						
Vaccines are safe x incentives	-0.04	0.02	0.00	0.07	0.12**	0.07
	(0.06)	(0.06)	(0.06)	(0.06)	(0.06)	(0.06)
Vaccines are not safe x incentives	-0.02	-0.02	-0.02	-0.06	-0.03	-0.09
	(0.06)	(0.06)	(0.06)	(0.06)	(0.06)	(0.07)
Vaccine triggers diseases:						
Vaccines trigger diseases x incentives	0.13*	0.09	0.11	-0.02	0.01	-0.06
	(0.07)	(0.07)	(0.07)	(0.08)	(0.08)	(0.09)
Vaccines do not trigger diseases x incentives	-0.11*	-0.04	-0.07	0.02	0.06	0.02
	(0.06)	(0.05)	(0.05)	(0.05)	(0.06)	(0.06)
Worries about side-effects:						
Worried about side-effect x incentives	0.01	0.01	0.00	-0.00	0.03	-0.05
	(0.06)	(0.06)	(0.06)	(0.07)	(0.07)	(0.08)
Not worried about side-effect x incentives	-0.06	-0.01	-0.02	0.00	0.05	0.03
	(0.06)	(0.06)	(0.06)	(0.06)	(0.06)	(0.06)
Worries of needles:						
Worried of needles x incentives	0.00	0.07	0.04	-0.01	0.04	-0.01
	(0.06)	(0.06)	(0.06)	(0.07)	(0.07)	(0.07)
Not worried of needles x incentives	-0.05	-0.07	-0.06	-0.00	0.02	-0.02
	(0.06)	(0.06)	(0.06)	(0.06)	(0.06)	(0.07)
Region x Age FE	yes	yes	yes	yes	yes	yes
Controls	yes	yes	yes	yes	yes	yes

Note: The table shows coefficient estimates from linear regressions of the standardized outcome on an indicator for the financial incentives condition interacted with indicators capturing in which quantile a participant's characteristic is (e.g., above or below the median). The corresponding coefficients indicate the total effect for each subgroup. Heteroscedasticity robust standard errors are shown in parentheses. All regressions use exactly the pre-registered controls, consisting of sociodemographics controls and age x region fixed effects.
* $p < 0.10$, ** $p < 0.05$, *** $p < 0.01$

Table S21: Heterogeneous treatment effects based on vaccine hesitancy on morals and civic responsibility, perceived safety and efficacy, and trust

Measure:	Outcomes					
	Moral and civic resp.	Donation for vacc.	Donation for pay. vacc.	Safety & effic. ind.	Vacc. safe children	Trust
Vaccine hesitancy (quintiles):						
1st quintile x incentives	0.10	-0.04	0.06	-0.02	-0.13	0.02
	(0.10)	(0.10)	(0.10)	(0.11)	(0.11)	(0.11)
2nd quintile x incentives	-0.05	-0.16	-0.03	-0.14	0.02	-0.01
	(0.10)	(0.10)	(0.10)	(0.10)	(0.10)	(0.10)
3rd quintile x incentives	0.06	0.02	0.02	0.07	0.12	0.06
	(0.09)	(0.10)	(0.09)	(0.09)	(0.08)	(0.09)
4th quintile x incentives	-0.13	-0.08	0.18*	-0.03	-0.09	-0.02
	(0.11)	(0.10)	(0.10)	(0.10)	(0.10)	(0.10)
5th quintile x incentives	0.00	0.14	0.11	-0.04	-0.05	-0.03
	(0.09)	(0.09)	(0.10)	(0.09)	(0.09)	(0.09)
Vaccine hesitancy (median split):						
More hesitant x incentives	0.05	-0.06	0.04	-0.03	-0.05	0.02
	(0.06)	(0.07)	(0.06)	(0.07)	(0.06)	(0.06)
Less hesitant x incentives	-0.05	0.02	0.10	-0.03	-0.01	-0.01
	(0.06)	(0.06)	(0.06)	(0.06)	(0.06)	(0.06)
Vaccine safety:						
Vaccines are safe x incentives	-0.02	0.02	0.08	-0.04	0.02	0.01
	(0.06)	(0.06)	(0.06)	(0.06)	(0.06)	(0.06)
Vaccines are not safe x incentives	0.01	-0.06	0.06	-0.03	-0.07	-0.01
	(0.06)	(0.06)	(0.06)	(0.06)	(0.06)	(0.06)
Vaccine triggers diseases:						
Vaccines trigger diseases x incentives	0.06	0.01	0.03	0.04	-0.00	0.10
	(0.07)	(0.08)	(0.08)	(0.08)	(0.08)	(0.07)
Vaccines do not trigger diseases x incentives	-0.03	-0.03	0.09*	-0.07	-0.04	-0.05
	(0.05)	(0.05)	(0.05)	(0.05)	(0.05)	(0.05)
Worries about side-effects:						
Worried about side-effect x incentives	0.04	-0.04	0.02	-0.05	-0.06	0.02
	(0.06)	(0.06)	(0.06)	(0.06)	(0.06)	(0.06)
Not worried about side-effect x incentives	-0.04	-0.00	0.11*	-0.01	0.00	-0.01
	(0.06)	(0.06)	(0.06)	(0.06)	(0.06)	(0.06)
Worries of needles:						
Worried of needles x incentives	0.05	0.06	0.16**	-0.01	-0.02	0.05
	(0.06)	(0.06)	(0.06)	(0.06)	(0.07)	(0.06)
Not worried of needles x incentives	-0.05	-0.10	-0.02	-0.05	-0.03	-0.04
	(0.06)	(0.06)	(0.06)	(0.06)	(0.06)	(0.06)
Region x Age FE	yes	yes	yes	yes	yes	yes
Controls	yes	yes	yes	yes	yes	yes

Note: The table shows coefficient estimates from linear regressions of the outcome on an indicator for the financial incentives condition interacted with indicators capturing in which quantile a participant's characteristic is (e.g., above or below the median). The corresponding coefficients indicate the total effect for each subgroup. Heteroscedasticity robust standard errors are shown in parentheses. All regressions use exactly the pre-registered controls, consisting of sociodemographics controls and age x region fixed effects.
* $p < 0.10$, ** $p < 0.05$, *** $p < 0.01$

Table S22: Heterogeneous treatment effects based on vaccine hesitancy on other health behaviors and feelings of regret

Measure:	Outcomes					
	Flu shot	Next flu shot	Blood donation	Paym. ethical	Regret index	Coercion feelings
Vaccine hesitancy (quintiles):						
1st quintile x incentives	0.13	0.14	-0.03	0.15	-0.13	-0.05
	(0.09)	(0.10)	(0.10)	(0.09)	(0.11)	(0.10)
2nd quintile x incentives	-0.00	-0.07	-0.06	-0.11	-0.15	-0.02
	(0.12)	(0.11)	(0.10)	(0.10)	(0.11)	(0.10)
3rd quintile x incentives	-0.09	-0.04	0.17	0.03	0.08	-0.00
	(0.08)	(0.09)	(0.10)	(0.10)	(0.08)	(0.09)
4th quintile x incentives	-0.03	0.09	-0.05	0.11	-0.02	-0.02
	(0.09)	(0.10)	(0.10)	(0.10)	(0.10)	(0.10)
5th quintile x incentives	-0.01	-0.07	-0.11	-0.06	0.01	0.03
	(0.10)	(0.09)	(0.08)	(0.10)	(0.09)	(0.09)
Vaccine hesitancy (median split):						
More hesitant x incentives	0.01	0.03	-0.05	0.01	-0.10	-0.04
	(0.06)	(0.06)	(0.06)	(0.06)	(0.07)	(0.06)
Less hesitant x incentives	-0.01	-0.01	0.01	0.04	0.02	0.01
	(0.06)	(0.06)	(0.06)	(0.06)	(0.06)	(0.06)
Vaccine safety:						
Vaccines are safe x incentives	0.03	-0.01	-0.00	-0.01	0.01	0.04
	(0.06)	(0.06)	(0.06)	(0.06)	(0.06)	(0.06)
Vaccines are not safe x incentives	-0.03	0.03	-0.04	0.05	-0.09	-0.07
	(0.06)	(0.06)	(0.06)	(0.06)	(0.06)	(0.06)
Vaccine triggers diseases:						
Vaccines trigger diseases x incentives	-0.03	0.02	-0.03	0.04	-0.05	-0.05
	(0.06)	(0.07)	(0.07)	(0.07)	(0.08)	(0.07)
Vaccines do not trigger diseases x incentives	0.02	0.00	-0.02	0.02	-0.03	0.01
	(0.06)	(0.05)	(0.05)	(0.05)	(0.05)	(0.05)
Worries about side-effects:						
Worried about side-effect x incentives	-0.02	0.02	-0.00	0.04	-0.09	-0.05
	(0.06)	(0.06)	(0.06)	(0.06)	(0.07)	(0.06)
Not worried about side-effect x incentives	0.02	0.01	-0.04	0.01	0.00	0.03
	(0.06)	(0.06)	(0.06)	(0.06)	(0.06)	(0.06)
Worries of needles:						
Worried of needles x incentives	0.02	0.03	-0.02	0.03	-0.05	-0.04
	(0.06)	(0.06)	(0.06)	(0.06)	(0.06)	(0.06)
Not worried of needles x incentives	-0.02	-0.01	-0.02	0.02	-0.03	0.01
	(0.06)	(0.06)	(0.06)	(0.06)	(0.06)	(0.06)
Region x Age FE	yes	yes	yes	yes	yes	yes
Controls	yes	yes	yes	yes	yes	yes

Note: The table shows coefficient estimates from linear regressions of the standardized outcome on an indicator for the financial incentives condition interacted with indicators capturing in which quantile a participant's characteristic is (e.g., above or below the median). The corresponding coefficients indicate the total effect for each subgroup. Heteroscedasticity robust standard errors are shown in parentheses. All regressions use exactly the pre-registered controls, consisting of sociodemographics controls and age x region fixed effects.
* $p < 0.10$, ** $p < 0.05$, *** $p < 0.01$

Table S23: Heterogeneous treatment effects based on sociodemographics on 2nd dose uptake

Measure:	Outcomes		
	2nd dose uptake	Shorter wait 2nd dose	2nd dose if 1st
Age:			
High age x incentives	0.08*	0.08*	0.04
	(0.05)	(0.05)	(0.04)
Low age x incentives	0.03	0.01	0.06
	(0.05)	(0.05)	(0.05)
Gender:			
Female x incentives	0.05	0.05	0.05
	(0.04)	(0.04)	(0.05)
Male x incentives	0.06	0.04	0.05
	(0.05)	(0.05)	(0.05)
Children:			
Has children x incentives	0.02	0.01	0.02
	(0.05)	(0.05)	(0.05)
Has no children x incentives	0.10**	0.08*	0.08*
	(0.05)	(0.05)	(0.05)
Single:			
Single x incentives	0.12*	0.12*	0.15**
	(0.07)	(0.06)	(0.07)
Non-single x incentives	0.03	0.02	0.01
	(0.04)	(0.04)	(0.04)
Income:			
High income x incentives	0.05	0.06	0.00
	(0.05)	(0.04)	(0.04)
Low income x incentives	0.06	0.03	0.09*
	(0.05)	(0.05)	(0.05)
College:			
College x incentives	0.03	0.04	0.03
	(0.04)	(0.04)	(0.04)
No college degree x incentives	0.08	0.06	0.07
	(0.05)	(0.05)	(0.06)
Region x Age FE	yes	yes	yes
Controls	yes	yes	yes

Note: The table shows coefficient estimates from linear regressions of the standardized outcome on an indicator for the financial incentives condition interacted with an indicator each for above and below median value of the participant characteristic. The corresponding coefficients indicate the total effect for each subgroup. Heteroscedasticity robust standard errors are shown in parentheses. All regressions use exactly the pre-registered controls, consisting of sociodemographics controls and age x region fixed effects.
* $p < 0.10$, ** $p < 0.05$, *** $p < 0.01$

Table S24: Heterogeneous treatment effects based on sociodemographics on 3rd dose uptake

Measure:	Outcomes					
	3rd dose intention	3rd dose SEK 100	3rd dose SEK 500	3rd dose	Short wait 3rd	3rd dose if 2nd
Age:						
High age x incentives	-0.03	0.01	-0.01	0.07	0.10	0.04
	(0.06)	(0.06)	(0.06)	(0.06)	(0.06)	(0.06)
Low age x incentives	-0.02	-0.01	-0.01	-0.09	-0.05	-0.08
	(0.06)	(0.06)	(0.06)	(0.07)	(0.07)	(0.07)
Gender:						
Female x incentives	-0.01	-0.01	-0.01	0.03	0.05	0.00
	(0.05)	(0.05)	(0.05)	(0.06)	(0.06)	(0.06)
Male x incentives	-0.05	0.02	-0.00	-0.06	-0.01	-0.04
	(0.07)	(0.07)	(0.07)	(0.07)	(0.07)	(0.08)
Children:						
Has children x incentives	-0.05	-0.01	-0.03	0.01	0.03	0.02
	(0.06)	(0.06)	(0.06)	(0.06)	(0.06)	(0.07)
Has no children x incentives	-0.00	0.01	0.01	-0.03	0.03	-0.06
	(0.06)	(0.06)	(0.06)	(0.07)	(0.07)	(0.07)
Single:						
Single x incentives	-0.03	-0.02	0.01	0.00	0.08	-0.05
	(0.08)	(0.08)	(0.08)	(0.09)	(0.09)	(0.09)
Non-single x incentives	-0.02	0.01	-0.01	-0.01	0.01	-0.00
	(0.05)	(0.05)	(0.05)	(0.05)	(0.05)	(0.06)
Income:						
High income x incentives	0.02	0.05	0.07	-0.03	0.03	-0.06
	(0.06)	(0.06)	(0.06)	(0.06)	(0.07)	(0.07)
Low income x incentives	-0.07	-0.04	-0.08	0.01	0.03	0.03
	(0.06)	(0.06)	(0.06)	(0.07)	(0.07)	(0.07)
College:						
College x incentives	-0.06	0.01	-0.01	0.03	0.08	0.02
	(0.06)	(0.06)	(0.06)	(0.06)	(0.06)	(0.06)
No college degree x incentives	0.02	-0.01	-0.01	-0.05	-0.04	-0.07
	(0.06)	(0.06)	(0.06)	(0.07)	(0.07)	(0.08)
Region x Age FE	yes	yes	yes	yes	yes	yes
Controls	yes	yes	yes	yes	yes	yes

Note: The table shows coefficient estimates from linear regressions of the standardized outcome on an indicator for the financial incentives condition interacted with an indicator each for above and below median value of the participant characteristic. The corresponding coefficients indicate the total effect for each subgroup. Heteroscedasticity robust standard errors are shown in parentheses. All regressions use exactly the pre-registered controls, consisting of sociodemographics controls and age x region fixed effects.
* $p < 0.10$, ** $p < 0.05$, *** $p < 0.01$

Table S25: Heterogeneous treatment effects based on sociodemographics on morals and civic responsibility, perceived safety and efficacy, and trust

Measure:	Outcomes					
	Moral and civic resp.	Donation for vacc.	Donation for pay. vacc.	Safety & effic. ind.	Vacc. safe children	Trust
Age:						
High age x incentives	-0.02	-0.06	0.06	-0.02	-0.02	-0.01
	(0.06)	(0.06)	(0.06)	(0.06)	(0.06)	(0.06)
Low age x incentives	0.02	0.02	0.08	-0.04	-0.03	0.02
	(0.06)	(0.06)	(0.06)	(0.06)	(0.06)	(0.06)
Gender:						
Female x incentives	-0.01	0.01	0.07	-0.04	-0.02	0.03
	(0.05)	(0.06)	(0.06)	(0.06)	(0.05)	(0.06)
Male x incentives	0.02	-0.06	0.06	-0.01	-0.04	-0.03
	(0.07)	(0.07)	(0.07)	(0.07)	(0.07)	(0.07)
Children:						
Has children x incentives	-0.02	-0.05	0.08	-0.03	-0.02	0.00
	(0.06)	(0.06)	(0.06)	(0.06)	(0.06)	(0.06)
Has no children x incentives	0.03	0.02	0.06	-0.03	-0.04	0.01
	(0.06)	(0.06)	(0.06)	(0.06)	(0.07)	(0.06)
Single:						
Single x incentives	0.02	0.14*	0.16*	-0.06	-0.07	-0.03
	(0.08)	(0.08)	(0.08)	(0.08)	(0.09)	(0.08)
Non-single x incentives	-0.01	-0.08	0.03	-0.02	-0.01	0.02
	(0.05)	(0.05)	(0.05)	(0.05)	(0.05)	(0.05)
Income:						
High income x incentives	0.04	0.04	0.16**	0.04	0.06	0.04
	(0.06)	(0.06)	(0.07)	(0.07)	(0.06)	(0.07)
Low income x incentives	-0.03	-0.07	-0.01	-0.09	-0.11*	-0.03
	(0.06)	(0.06)	(0.06)	(0.06)	(0.06)	(0.06)
College:						
College x incentives	-0.02	0.04	0.03	-0.00	-0.02	0.06
	(0.06)	(0.06)	(0.06)	(0.06)	(0.06)	(0.06)
No college degree x incentives	0.02	-0.09	0.12*	-0.06	-0.03	-0.07
	(0.06)	(0.07)	(0.06)	(0.07)	(0.06)	(0.07)
Region x Age FE	yes	yes	yes	yes	yes	yes
Controls	yes	yes	yes	yes	yes	yes

Note: The table shows coefficient estimates from linear regressions of the outcome on an indicator for the financial incentives condition interacted with an indicator each for above and below median value of the participant characteristic. The corresponding coefficients indicate the total effect for each subgroup. Heteroscedasticity robust standard errors are shown in parentheses. All regressions use exactly the pre-registered controls, consisting of sociodemographics controls and age x region fixed effects.
* $p < 0.10$, ** $p < 0.05$, *** $p < 0.01$

Table S26: Heterogeneous treatment effects based on sociodemographics on other health behaviors and feelings of regret

Measure:	Outcomes					
	Flu shot	Next flu shot	Blood donation	Paym. ethical	Regret index	Coercion feelings
Age:						
High age x incentives	0.01	0.06	-0.06	0.08	-0.04	-0.02
	(0.07)	(0.07)	(0.06)	(0.06)	(0.06)	(0.06)
Low age x incentives	-0.01	-0.03	0.01	-0.02	-0.04	-0.00
	(0.05)	(0.06)	(0.06)	(0.06)	(0.06)	(0.06)
Gender:						
Female x incentives	0.02	-0.02	-0.02	0.07	0.04	0.00
	(0.06)	(0.06)	(0.05)	(0.06)	(0.05)	(0.06)
Male x incentives	-0.02	0.06	-0.02	-0.03	-0.17**	-0.03
	(0.06)	(0.07)	(0.07)	(0.07)	(0.07)	(0.07)
Children:						
Has children x incentives	-0.00	0.03	-0.03	0.08	-0.05	-0.07
	(0.06)	(0.06)	(0.06)	(0.06)	(0.06)	(0.06)
Has no children x incentives	0.01	-0.01	-0.01	-0.04	-0.03	0.06
	(0.06)	(0.06)	(0.06)	(0.06)	(0.06)	(0.06)
Single:						
Single x incentives	0.06	0.07	-0.08	0.03	-0.05	0.05
	(0.09)	(0.08)	(0.08)	(0.08)	(0.08)	(0.08)
Non-single x incentives	-0.02	-0.01	-0.00	0.02	-0.04	-0.03
	(0.05)	(0.05)	(0.05)	(0.05)	(0.05)	(0.05)
Income:						
High income x incentives	-0.03	0.07	-0.06	0.13**	-0.08	-0.07
	(0.06)	(0.06)	(0.06)	(0.06)	(0.06)	(0.06)
Low income x incentives	0.02	-0.04	0.02	-0.07	-0.00	0.04
	(0.06)	(0.06)	(0.06)	(0.06)	(0.06)	(0.06)
College:						
College x incentives	0.03	0.00	0.01	-0.02	0.04	0.03
	(0.06)	(0.06)	(0.06)	(0.06)	(0.05)	(0.06)
No college degree x incentives	-0.03	0.02	-0.05	0.08	-0.14**	-0.06
	(0.06)	(0.06)	(0.06)	(0.06)	(0.07)	(0.07)
Region x Age FE	yes	yes	yes	yes	yes	yes
Controls FE	yes	yes	yes	yes	yes	yes

Note: The table shows coefficient estimates from linear regressions of the standardized outcome on an indicator for the financial incentives condition interacted with an indicator each for above and below median value of the participant characteristic. The corresponding coefficients indicate the total effect for each subgroup. Heteroscedasticity robust standard errors are shown in parentheses. All regressions use exactly the pre-registered controls, consisting of sociodemographics controls and age x region fixed effects.
* $p < 0.10$, ** $p < 0.05$, *** $p < 0.01$

Table S27: Within participant changes in worries about side-effects and safety perceptions. Using the second and third specification described in the introduction of this section, this table reports the heterogeneous treatment effects for each condition based on measurements of the same item before and after the intervention for each participant. We find no impact on changes in worries about side-effects and safety perceptions within participants. We also do not find any impact on changes within participants depending on the level of safety perceptions and worries about side-effects before the intervention.

Table S27: Within participant changes in worries about side-effects and safety perceptions

Dependent Variable	Change within participant			
	COVID-19 vacc. are safe	Not worried side-effects	COVID-19 vacc. are safe	Not worried side-effects
	(1)	(2)	(3)	(4)
Financial incentives condition	-0.06	-0.02	-0.03	-0.02
	(0.04)	(0.04)	(0.03)	(0.03)
Safe x incentives			0.02	
			(0.04)	
COVID-19 vaccines are safe			-0.68***	
			(0.02)	
Side-effects x incentives				-0.00
				(0.03)
Not worried about side-effects				-0.69***
				(0.02)
Age x Region FE	yes	yes	yes	yes
Controls	yes	yes	yes	yes
Observations	3,238	3,238	3,238	3,238

Note: The table shows coefficient estimates from linear regressions of changes in the outcome (standardized) on an indicator for the financial incentives condition in columns (1) and (2) and interacted with the baseline level of the outcome in columns (3) and (4) (standardized). The changes are first computed based on the non-standardized values (since items are measured on the same scale) and then standardized for the regressions. Heteroscedasticity robust standard errors are shown in parentheses. All regressions use exactly the pre-registered controls, consisting of sociodemographics controls and age x region fixed effects.
* $p < 0.10$, ** $p < 0.05$, *** $p < 0.01$

2.4.4 Results for single items of indices

Tables S28 to S32: Treatment effects on each single item of the indices. These tables show all coefficient estimates for each single (and standardized) item. The following tables provide the effect sizes from our main specification with the pre-registered set of controls for the indices alongside each single item that the respective index is composed of (see the Methods section for details on the composition of each index). The results for single items confirm the main results. Figure S17 shows the corresponding coefficient estimates graphically.

Table S28: Single items: morals and civic responsibility

Dependent Variable	Morals and civic resp. index	Pers. costs greater good	Civic duty and moral obl.	Socially appropriate
	(1)	(2)	(3)	(4)
Financial incentives condition	0.00	-0.03	-0.00	0.03
	(0.04)	(0.04)	(0.04)	(0.04)
Age x Region FE	yes	yes	yes	yes
Controls	yes	yes	yes	yes
Observations	3,238	3,238	3,238	3,238

Note: The table shows coefficient estimates from linear regressions of the standardized outcome on an indicator for the financial incentives condition. Heteroscedasticity robust standard errors are shown in parentheses. All regressions use exactly the pre-registered controls, consisting of sociodemographics controls and age x region fixed effects.
* $p < 0.10$, ** $p < 0.05$, *** $p < 0.01$

Table S29: Single items: safety and efficacy

Dependent Variable	Safety and efficacy index	COVID-19 vacc. safe	Not worried side-effects	COVID-19 vacc. effective
	(1)	(2)	(3)	(4)
Financial incentives condition	-0.03	-0.03	-0.03	-0.01
	(0.04)	(0.04)	(0.04)	(0.04)
Age x Region FE	yes	yes	yes	yes
Controls	yes	yes	yes	yes
Observations	3,238	3,238	3,238	3,238

Note: The table shows coefficient estimates from linear regressions of the standardized outcome on an indicator for the financial incentives condition. Heteroscedasticity robust standard errors are shown in parentheses. All regressions use exactly the pre-registered controls, consisting of sociodemographics controls and age x region fixed effects.
* $p < 0.10$, ** $p < 0.05$, *** $p < 0.01$

Table S30: Single items: trust in vaccination provision

Dependent Variable	Trust in vacc. provision index	pharmac. comp.	Trust in publ. health agency	researchers
	(1)	(2)	(3)	(4)
Financial incentives condition	0.00	-0.01	-0.00	0.02
	(0.04)	(0.04)	(0.04)	(0.04)
Age x Region FE	yes	yes	yes	yes
Controls	yes	yes	yes	yes
Observations	3,238	3,238	3,238	3,238

Note: The table shows coefficient estimates from linear regressions of the standardized outcome on an indicator for the financial incentives condition. Heteroscedasticity robust standard errors are shown in parentheses. All regressions use exactly the pre-registered controls, consisting of sociodemographics controls and age x region fixed effects.
* $p < 0.10$, ** $p < 0.05$, *** $p < 0.01$

Table S31: Single items: payment for vaccination is ethical

Dependent Variable	Payments ethical index	Payments ethical	Support SEK 500 paym.
	(1)	(2)	(3)
Financial incentives condition	0.03	0.04	0.00
	(0.04)	(0.04)	(0.04)
Age x Region FE	yes	yes	yes
Controls	yes	yes	yes
Observations	3,238	3,238	3,238

Note: The table shows coefficient estimates from linear regressions of the standardized outcome on an indicator for the financial incentives condition. Heteroscedasticity robust standard errors are shown in parentheses. All regressions use exactly the pre-registered controls, consisting of sociodemographics controls and age x region fixed effects.
* $p < 0.10$, ** $p < 0.05$, *** $p < 0.01$

Table S32: Single items: feelings of regret

Dependent Variable	Feels no regret index	No regrets taking vacc.	Enough info. collected
	(1)	(2)	(3)
Financial incentives condition	-0.04<br>(0.04)	0.01<br>(0.04)	-0.07*<br>(0.04)
Age x Region FE	yes	yes	yes
Controls	yes	yes	yes
Observations	3,238	3,238	3,238

Note: The table shows coefficient estimates from linear regressions of the standardized outcome on an indicator for the financial incentives condition. Heteroscedasticity robust standard errors are shown in parentheses. All regressions use exactly the pre-registered controls, consisting of sociodemographics controls and age x region fixed effects.
* $p < 0.10$, ** $p < 0.05$, *** $p < 0.01$

Figure S17: Indices and single items

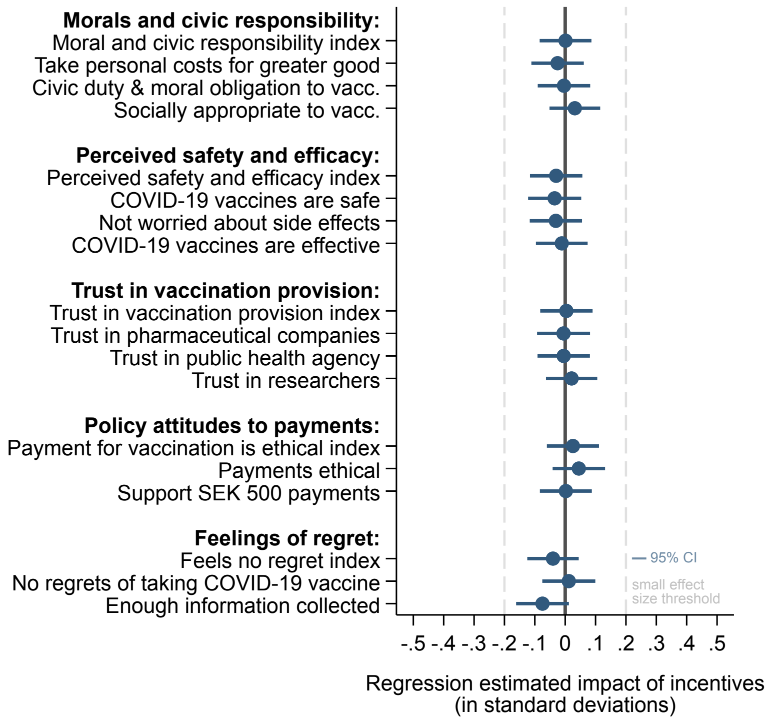

2.4.5 Secondary and exploratory outcome variables

Table S33 and S34: Treatment effects on secondary and exploratory variables. This table provides the effect sizes from our main specification with the pre-registered set of controls for secondary and exploratory outcome variables. Table S34 includes additional measure on intentions to take a 3rd dose. The tables shows that offering financial incentives does not have a statistically significant negative impact on any of the secondary and exploratory outcomes.

Table S33: Secondary and exploratory outcomes

Dependent Variable	Effectiveness of payments for vaccination	Will follow child vacc. plan	Partner vaccinated	Parents vaccinated	Support of vaccination mandate	Support of payment for blood donation
	(1)	(2)	(3)	(4)	(5)	(6)
Financial incentives condition	-0.00	-0.05	-0.07	-0.04	-0.06	0.05
	(0.04)	(0.05)	(0.05)	(0.05)	(0.04)	(0.04)
Age x Region FE	yes	yes	yes	yes	yes	yes
Controls	yes	yes	yes	yes	yes	yes
Observations	3,238	3,238	2,405	3,101	3,238	3,238

Note: The table shows coefficient estimates from linear regressions of the standardized outcome on an indicator for the financial incentives condition. Heteroscedasticity robust standard errors are shown in parentheses. Partner and parents vaccinated have lower number of observations since some participants are single or have no living parents. "Effectiveness of payments for vaccination" captures what share of Swedish residents, according to the guess of the participant, would have vaccinated had everyone been paid 200 SEK to get vaccinated in Spring 2021 (on a scale from 70% to 100%). "Will follow child vaccination plan" captures whether parents would follow the Swedish child vaccination schedule including vaccinations against measles and polio (5-point Likert scale). "Partner vaccinated" is 1 if the participant has a partner who is vaccinated and 0 for participants who have a partner who is not vaccinated. "Parents vaccinated" is 1 if the participant has at least 1 living parent who is vaccinated and 0 if the participant has at least 1 living parent but none of the parents is vaccinated. "Support of vaccination mandate" captures the support of a COVID-19 vaccination mandate for health care workers on a 5-point Likert scale. "Support of payments for blood donation" captures the support of payments for blood donations on a 5-point Likert scale. All regressions use exactly the pre-registered controls, consisting of sociodemographics controls and age x region fixed effects.
* $p < 0.10$, ** $p < 0.05$, *** $p < 0.01$

Table S34: Additional outcomes on 3rd dose intention

Dependent Variable	Reminder uptake	Inform. link click	Shorter wait intention 3rd dose
	(1)	(2)	(3)
Financial incentives condition	0.10**	-0.03	-0.01
	(0.04)	(0.04)	(0.04)
Age x Region FE	yes	yes	yes
Controls	yes	yes	yes
Observations	3,238	3,238	3,238

Note: The table shows coefficient estimates from linear regressions of the standardized outcome on an indicator for the financial incentives condition. Heteroscedasticity robust standard errors are shown in parentheses. "Reminder uptake": We offered participants in the first survey to send them a reminder email when the third dose of a COVID-19 vaccine becomes available for people in their region. We recorded whether participants chose to sign-up for the reminder. "Information click": At the end of the first survey, participants were presented with a link to a website with information on when the third dose of a COVID-19 vaccine would become available in the participant's region. We recorded whether participants clicked on the link. "Shorter wait intention 3rd dose": We asked participants, once the third dose is available to them, how long they plan to wait before getting it (reverse coded). All regressions use exactly the pre-registered controls, consisting of sociodemographics controls and age x region fixed effects.
* $p < 0.10$, ** $p < 0.05$, *** $p < 0.01$

2.5 Results Swedish complementary study

2.5.1 Overview

In sections 2.5.2 and 2.5.3, we provide descriptive statistics of our sample and a comparison with the Swedish population. We find that our sample is representative with respect to age, gender, income, and education.

In section 2.5.4, we provide the results of our main analysis. We examine whether people react differently when they are told that the government paid for vaccination or that researchers paid for vaccination. We do not find statistically significant treatment differences for any of the outcome measures. In section 2.5.5, we show that equivalence testing further confirms that there were no meaningful treatment effects across all outcomes.

In sections 2.5.6 and 2.5.7, we show that results are robust to including no controls, different sets of control variables, and using different inclusion criteria. In section 2.5.8, we explore whether any of the treatment effects differ based on vaccination hesitancy as well as sociodemographics such as income, education, age, and gender. We find that treatment effects are similarly mute across all subgroups.

Finally, in section 2.5.9 we investigate whether mentioning that a study is about COVID-19 vaccination in the consent form leads to sample selection. We conclude that mentioning that a study is about COVID- 19 vaccination in the consent form does not have a meaningful impact on selection into the study.

2.5.2 Summary statistics

Table S35 below provides descriptive statistics of our sample. The table first reports the summary statistics for our outcome variables. Note that all the presented indices are standardized. All other outcome variables are not standardized in Table S35, but are standardized for all analyses. We then report the proportion randomly assigned to the researcher condition. Finally, we report the summary statistics for the background variables, including participants' sociodemographics and answers to vaccine and COVID-19 specific questions measured *before* the incentives treatment (see SI Section 2.5.8).

Table S35: Summary statistics

Variable	Mean	SD	Min.	Max.	N
Outcome variables					
Additional dose	0.27	0.44	0.0	1.0	1,001
Additional dose new outbreak	0.72	0.45	0.0	1.0	1,001
Perceived safety and efficacy index	0.00	1.00	-2.6	1.5	1,001
Moral and civic responsibility index	0.00	1.00	-2.3	1.2	1,001
Treatment					
Researcher condition	0.51	0.50	0.0	1.0	1,001
Sociodemographics					
Age	31.56	8.47	18.0	48.0	1,001
Female	0.54	0.50	0.0	1.0	1,001
Elementary School of Lower	0.04	0.19	0.0	1.0	1,001
High-school	0.42	0.49	0.0	1.0	1,001
Professional Training	0.12	0.33	0.0	1.0	1,001
In College	0.09	0.29	0.0	1.0	1,001
College Degree	0.32	0.47	0.0	1.0	1,001
PhD	0.01	0.08	0.0	1.0	1,001
Income 0-5000kr	0.05	0.22	0.0	1.0	1,001
Income 5001-10000kr	0.05	0.22	0.0	1.0	1,001
Income 10001-15000kr	0.10	0.30	0.0	1.0	1,001
Income 15001-20000kr	0.11	0.31	0.0	1.0	1,001
Income 20001-25000kr	0.19	0.39	0.0	1.0	1,001
Income 25001-30000kr	0.22	0.41	0.0	1.0	1,001
Income 30001-35000kr	0.12	0.33	0.0	1.0	1,001
Income 35001-40000kr	0.08	0.27	0.0	1.0	1,001
Income 40001-45000kr	0.04	0.19	0.0	1.0	1,001
Income 45001-50000kr	0.03	0.16	0.0	1.0	1,001
Income 50001-55000kr	0.01	0.08	0.0	1.0	1,001
Income more than 55000kr	0.02	0.13	0.0	1.0	1,001
COVID-19 related baseline var.					
1st dose (vaccination status)	0.90	0.31	0.0	1.0	1,001
2nd dose	0.88	0.33	0.0	1.0	1,001
3rd dose	0.52	0.50	0.0	1.0	1,001
Vaccine efficacy	2.65	0.89	1.0	4.0	1,001
Vaccine safety	3.02	0.80	1.0	4.0	1,001
Trust researchers	3.83	1.19	1.0	5.0	1,001
Trust public health agency	3.71	1.17	1.0	5.0	1,001

2.5.3 General population sample

Next we compare the demographic composition of our general population sample in the survey data with the Swedish population (considering the same age restriction of ages 18 to 49). The survey company can only provide a sample that is representative in terms of age, gender, and region. However, we also compare other characteristics to the general population. In addition to age and gender, we also document how our sample compares to the general population in terms of average income and education.

We obtained the Swedish population data from the public registry data reported by Statistics Sweden. The sample is similar to the general Swedish population in terms of age, gender, education, and income.

Table S36: Trial sample, survey sample, and the Swedish population

**Variable**	Survey data			Sweden Ages 18-49
	N	Mean	SD	Mean
Age	1001	31.56	8.47	33.71
Female	1001	.54	.5	0.49
Share university education	1001	.42	.49	0.44
Average monthly income (SEK)	1001	24843	11514	24,211*

Note: Comparison of the survey data on age, gender, university education and average income with public registry data from Statistics Sweden. We constructed the variables such that they match the Statistics Sweden definition, with two exceptions: * is based on age >19.

2.5.4 Treatment effects

In this section, we provide the results of our main analysis. We use a linear regression to estimate treatment effects using ordinary least squares (OLS) regressions with heteroscedasticity-robust standard errors. We control for gender, age, education and income. In SI section 2.5.6 we show that results are robust to including no control variables.

The table and the figure show that we do not find statistically significant treatment differences for any of the outcome measures, and none of the 95% confidence intervals crosses the Cohen's d small effect size threshold of an effect of 0.2 standard deviations.

Figure S18: Treatment effects Swedish complementary study

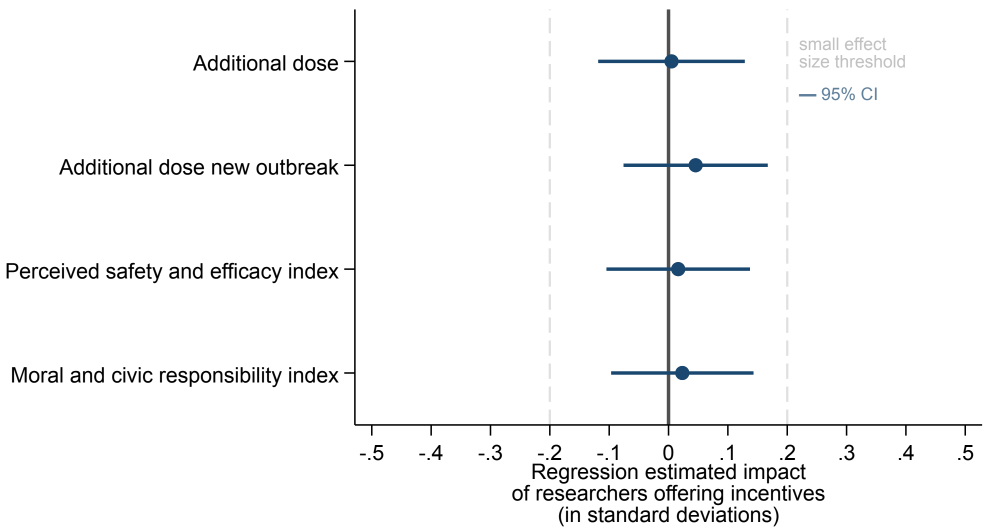

Table S37: Treatment effects Swedish complementary study

Dependent Variable	Additional dose	Add. dose new outbreak	Safety and efficacy	Moral and civic responsibility
	(1)	(2)	(3)	(4)
Researcher condition	0.01	0.05	0.02	0.02
	(0.06)	(0.06)	(0.06)	(0.06)
Controls	yes	yes	yes	yes
Observations	1001	1001	1001	1001

Note: The table shows coefficient estimates from linear regressions of the standardized outcome on an indicator for the researcher condition. Heteroscedasticity robust standard errors are shown in parentheses. All regressions control for sociodemographics (gender, age, education and income).
* $p < 0.10$, ** $p < 0.05$, *** $p < 0.01$

2.5.5 Equivalence testing

In this section we use equivalence tests to study whether we can reject meaningful differences between the researcher and the government condition (*54*, *55*). For each outcome, we test the null hypothesis that the estimated effect size is larger than a small effect benchmark.

Equivalence tests require researchers to specify the smallest effect size of interest (SESOI) as a benchmark (see discussion in SI section 2.2.4). We specify the SESOI as 0.20, which corresponds

both to i) the minimum effect size that the study design has power to detect (*55*), as stated in our pre-registration plan, and ii) the definition of a small effect size of 0.2 standard deviations based on Cohen's d (*76*).

We use the two one-sided t-tests (TOST) procedure (*55*). Accordingly, we do a one-sided t-test of each estimated effect size being more negative than the SESOI of -0.2 and a one-sided t-test of each estimated effect size being more positive than the SESOI of 0.2. When both one-sided tests can be statistically rejected, we can reject the hypothesis that the absolute value of a treatment effect is 0.2 standard deviations or larger.

Table S38 provides the results of these equivalence tests. The leftmost column shows the results of testing based on the null hypothesis that the effect size is more negative than -0.2 standard deviations. The middle column shows the results of testing based on the null hypothesis that the effect size is more positive than 0.2 standard deviations. The rightmost column then shows the maximal p-value of both tests. We can clearly reject impacts more negative (positive) than -0.2 (0.2) standard deviations for each outcome. All tests are highly statistically significant, with the largest p-value among all outcomes being 0.0065. Hence, for each outcome, we can rule out even a small effect of 0.2 standard deviations. We conclude that there is strong evidence for the absence of even small effects.

Table S38: Equivalence testing

Outcome	P-values equivalence testing		
	H0: Effect $<$ -0.2	H0: Effect $>$ 0.2	max P-value
Additional dose	0.000584	0.001006	0.001006
Additional dose new outbreak	0.000039	0.006462	0.006462
Perceived safety and efficacy index	0.000235	0.001482	0.001482
Moral and civic responsibility index	0.000137	0.001964	0.001964

2.5.6 Different sets of controls

Figures S19 to S22: Treatment effects including different sets of controls. For our main analysis we control for gender, age, education and income. The following figures show that our results are robust to including different sets of control variables.

Figure S19: Different controls: no controls

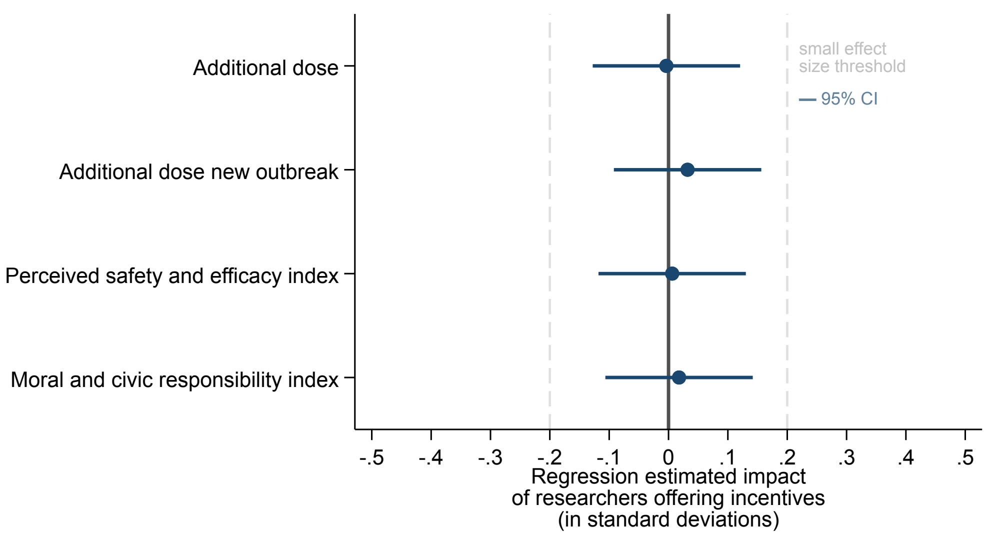

Figure S20: Different controls: controlling for age

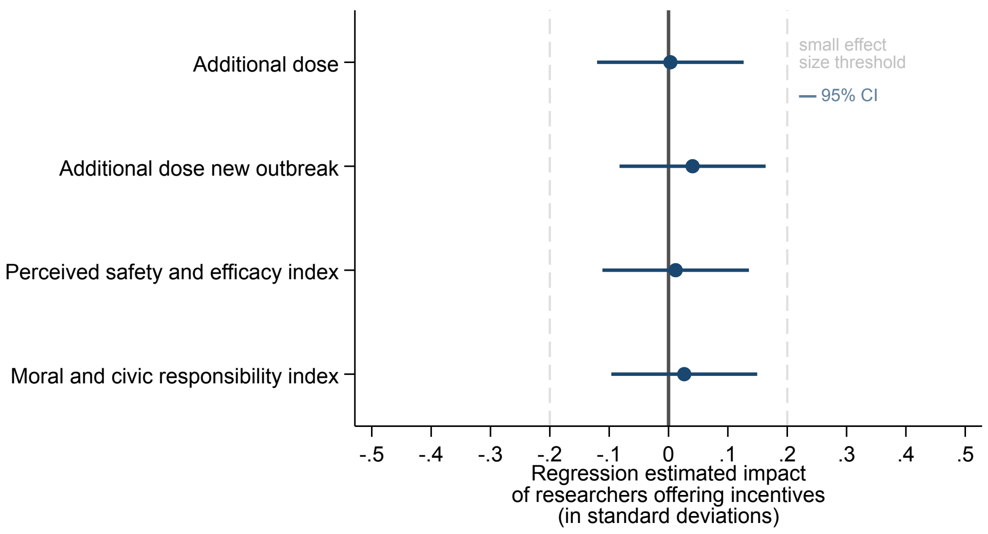

Figure S21: Different controls: controlling for age and gender

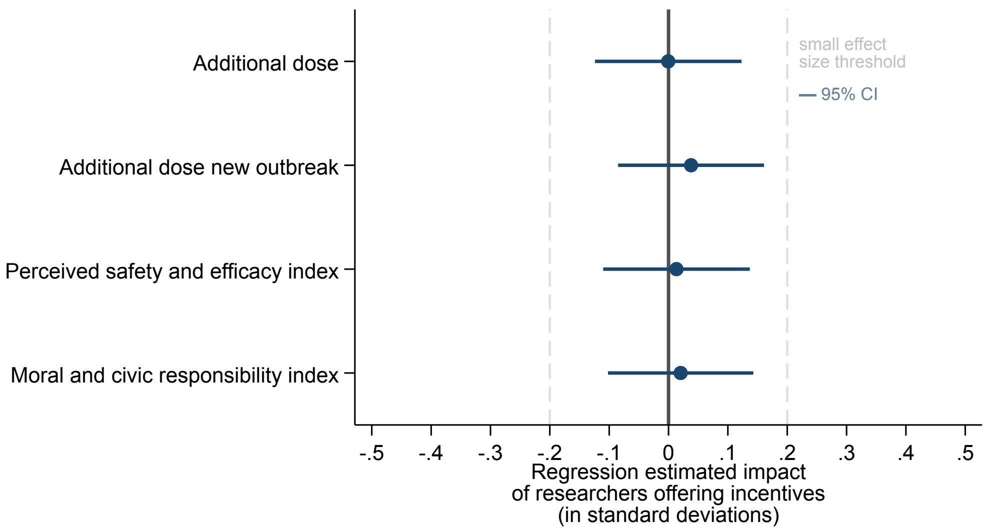

Figure S22: Different controls: controlling for age, gender and education

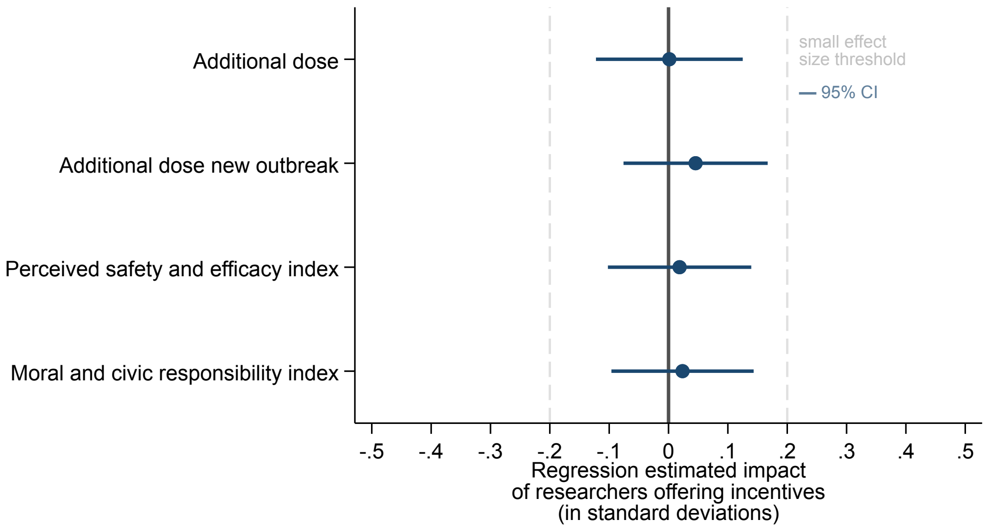

2.5.7 Treatment effects for individuals that were not aware of the Swedish RCT.

Figure S23: Treatment effects for individuals that were not aware of the Swedish RCT After we described the Swedish COVID-19 incentive program (the earlier RCT) to the participants, we asked them whether they had heard about this program before (see SI section 3). We find that most participants were indeed not aware of the RCT; only 10.49% say that they have definitively or probably heard about the RCT. Also, awareness does not differ across the two treatment conditions (t-test, t = 0.82, p = 0.411). Figure S23 shows that treatment estimates do not change when we only include participants that were not aware of the RCT (N = 896).

Figure S23: Focusing on individuals that were not aware of the Swedish RCT

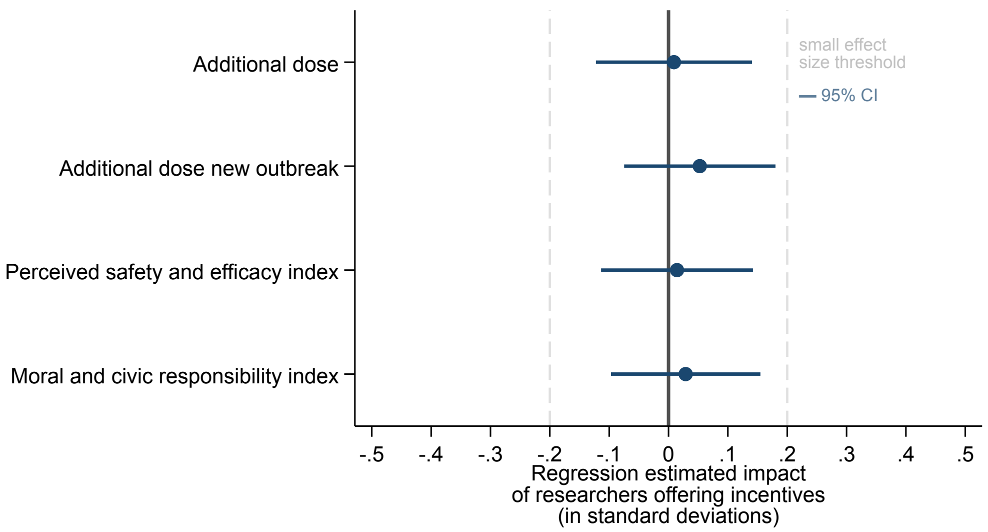

2.5.8 Treatment effects based on participants' characteristics

This section explores potential heterogeneous impacts of the researcher condition on the outcomes based on participants' characteristics. We do heterogeneity analyses based on participants' sociodemographics and vaccination attitudes. Importantly, all characteristics were measured before assignment to the government or researcher incentives condition.

Summary of results: We do not find any robust heterogeneities across 72 coefficient estimates. Despite the large number of coefficients tested, no coefficient is statistically significant at the 1%

level and only 1 coefficient is statistically significant at the 5% level. Hence, there is no indication that there were consistent heterogeneities based on background characteristics.

Variable definitions: We consider gender, age, education, income. Moreover, we consider the following measures of vaccine hesitancy:

- **Vaccination status**: We asked participants whether they have taken a first dose of a COVID-19 vaccine (scale from 0 to 1)
- **Vaccine safety**: We asked participants whether they think that the first two doses of the currently approved COVID-19 vaccines are safe (scale from 1 to 4).
- **Vaccine efficacy**: We asked participants how effective they think it would be for them to take an additional shot of a COVID-19 vaccine (scale from 1 to 4).
- **Trust researchers**: We asked participants whether they trust the Swedish health authorities when it comes to the COVID-19 vaccines (scale from 1 to 5).
- **Trust public health agency**: We asked participants whether they trust the researchers who are studying the effects of the COVID-19 vaccines (scale from 1 to 5).

We provide results on heterogeneities across vaccine attitudes and sociodemographics. To this end, we split the continuous variables (age, income, vaccine safety, vaccine efficacy, trust researchers, and trust public health agency) based on median values.

Specification: We use an OLS model where we interact an indicator for the researcher condition with an indicator capturing whether participant's characteristic is above or below the median. We use the following specification:

$$Outcome_i = b_0 + b_1 * 1(Above\_median)_i + b_2 * 1(Researcher\_condition)_i * 1(Above\_median)_i$$
$$+ \mathrm{b}_3 * 1(Researcher\_condition)_i * 1(Below\_median)_i + b_4 * X_i + e_i$$

where $1(Researcher\_condition)_i$ has a value of 1 if participant i is in the researcher condition and a value of 0 otherwise, $1(Above\_median)_i$ has a value of 1 if the participant is above the median of a certain socio-demographic characteristic or COVID-19 vaccination attitude, and $1(Below\_median)_i$ has a value of 1 if the participant is below the corresponding median.

The coefficients for the interactions between researcher condition and these indicator (b_2 and b_3) variables give the *total* impact of the researchers offering financial incentives (instead of the

government) on the participant group with below median values and separately for the participant group with above median values. That is, the interaction effect can be interpreted as the total effect of being in the researcher condition given the participant characteristic.

X_i is the vector of control variables used in our main specification and e_i is an individual specific error robust to heteroscedasticity. We indicate the interaction for participants with above median values of a characteristic with "high" (e.g., high age is above median age) and for participants with below median values of a characteristic as "low" (e.g., low age is below median age).

Table S39: Heterogeneous treatment effects based on sociodemographics and vaccine hesitancy. Using the specification described in the introduction of this section, this table reports the heterogeneous treatment effects for based on participants' sociodemographics and vaccine hesitancy. As outlined in detail above, there is no indication of systematic treatment effect.

Table S39: Heterogeneous treatment effects Swedish complementary study

Measure:	Outcomes			
	Additional dose	Additional dose new outbreak	Perceived safety and efficacy index	Moral and civic responsibility index
Gender:				
Female x researcher cond.	0.04	-0.02	-0.02	-0.07
	(0.09)	(0.08)	(0.08)	(0.08)
Male x researcher cond.	-0.03	0.13	0.06	0.13
	(0.09)	(0.09)	(0.09)	(0.09)
Age:				
High age x researcher cond.	-0.05	0.02	0.02	0.02
	(0.09)	(0.08)	(0.09)	(0.09)
Low age x researcher cond.	0.06	0.07	0.02	0.03
	(0.08)	(0.09)	(0.08)	(0.09)
College:				
College x researcher cond.	-0.15	-0.01	0.03	-0.07
	(0.10)	(0.09)	(0.10)	(0.09)
No college degree x researcher cond.	0.11	0.08	0.01	0.10
	(0.08)	(0.09)	(0.08)	(0.08)
Income:				
High income x researcher cond.	-0.02	0.15*	0.01	0.02
	(0.09)	(0.08)	(0.09)	(0.08)
Low income x researcher cond.	0.03	-0.06	0.02	0.03
	(0.09)	(0.09)	(0.09)	(0.09)
Vaccination status:				
Vaccinated x researcher cond.	0.22**	0.13	0.03	-0.01
	(0.10)	(0.11)	(0.17)	(0.15)
Unvaccinated x researcher cond.	-0.02	0.02	0.00	0.01
	(0.07)	(0.06)	(0.06)	(0.06)
Vaccine safety:				
Safe x researcher cond.	-0.08	0.10	-0.04	0.02
	(0.13)	(0.06)	(0.08)	(0.08)
Not safe x researcher cond.	0.05	0.04	0.07	0.05
	(0.07)	(0.08)	(0.07)	(0.07)
Vaccine efficacy:				
Effective x researcher cond.	0.15	0.06	0.06	0.11
	(0.17)	(0.06)	(0.11)	(0.09)
Not effective x researcher cond.	-0.04	0.04	-0.00	-0.01
	(0.06)	(0.07)	(0.07)	(0.07)
Trust researchers:				
Hight trust x researcher cond.	-0.10	0.06	0.07	0.09
	(0.11)	(0.06)	(0.06)	(0.06)
Low trust x researcher cond.	0.06	0.01	-0.06	-0.05
	(0.07)	(0.09)	(0.07)	(0.08)
Trust public health agency:				
High trust x researcher cond.	0.05	0.06	0.03	0.06
	(0.13)	(0.05)	(0.07)	(0.07)
Low trust x researcher cond.	-0.02	0.03	-0.01	-0.01
	(0.07)	(0.08)	(0.07)	(0.07)
Controls	yes	yes	yes	yes

Note: The table shows coefficient estimates from linear regressions of the standardized outcome on an indicator for the researcher condition interacted with an indicator capturing a participant's characteristics. The corresponding coefficients indicate the total effect for each subgroup. Heteroscedasticity robust standard errors are shown in parentheses. All regressions controls for age, gender, income and education.
* $p < 0.10$, ** $p < 0.05$, *** $p < 0.01$

2.5.9 Treatment effects selection experiment

As part of the Swedish complementary study, we also investigated whether mentioning that a study is about COVID-19 vaccination in the consent form leads to sample selection.

Only a very small proportion of participants, namely 1.99%, discontinued the survey on the consent form screen and 96.30% of all participants completed the survey. Drop out rates on the consent form screen and completion rates do not differ across the treatment conditions. These results suggest that mentioning that the study is about COVID-19 vaccination in the consent form did not lead to sample selection.

To further study selection into the study, we regress sociodemographics and vaccination attitudes (see the definition in SI section 2.5.7) on an indicator variable that is 1 if the participant was in the treatment that mentioned that the study is about COVID-19 vaccination in the consent form and 0 otherwise. Our pre-registered main outcome measure is "No 2nd dose", which measures whether a participants got a second dose of the COVID-19 vaccine. Figure S24 provides the estimated treatment effects. Overall, we do not find that mentioning that the study is about COVID-19 vaccination in the consent form impacted selection into the study.

Figure S24: Treatment effects selection experiment

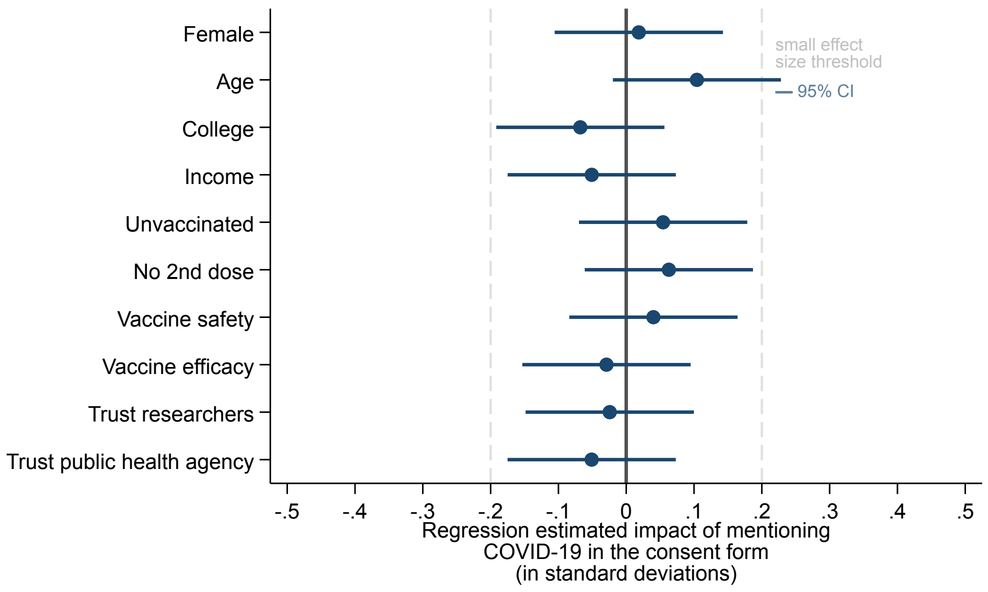

2.6 Results US study

2.6.1 Balance and survey participation

Of 3,980 participants who participated in the information provision survey, 3,062 also participated in the follow-up survey. There was no substantial or statistically significant difference across conditions, with a 76.15% survey participation rate in the incentives condition and a 77.93% participation rate in the control condition (see also column 1 in Table S40).

Regression results confirm equivalent participation rates across conditions. Table S40 first tests whether follow-up survey participation and completion differed by condition. Participants in the incentives condition did not participate at a different rate than participants in the control condition.

Tables S41 and S42 examine whether follow-up survey participation in both treatment conditions is balanced in terms of sociodemographics and COVID-19 related variables (measured before survey participation).

To do so, we use an OLS specification to regress each of these variables on "participated", an indicator for follow-up survey participation, and "participated x financial", the interaction of whether the subject participated and an indicator for being in the incentives condition. We then report the corresponding coefficient, standard errors, and significance levels. We do not see substantial differences in overall survey participation or condition-specific survey participation across different variables. This implies that survey participation is indeed balanced across the treatment conditions. SI Section 2.6.6 also shows no substantial differences in the coefficients of our main results when using different sets of controls, again confirming random assignment and no differential survey participation.

Table S40: Participation follow-up survey by condition

Dependent Variable	Survey participation
	(1)
Incentives condition	-0.02<br>(0.01)
Observations	3980

Note: The table shows coefficient estimates from linear regressions of an indicator capturing participation in the follow-up survey on an indicator for the incentives condition. Heteroscedasticity robust standard errors are shown in parentheses.
* $p < 0.10$, ** $p < 0.05$, *** $p < 0.01$

Table S41: Survey participation based on COVID-19 related variables

Dependent Variable	Unvaccinated	Tested positive	Vacc. safe	Vacc. effective	Willingness add. shot	Benefit add. shot	Vacc. cause disease	Trust CDC
	(1)	(2)	(3)	(4)	(5)	(6)	(7)	(8)
Participated	0.02	0.09	-0.06	-0.02	-0.07	-0.01	0.02	-0.04
	(0.05)	(0.05)	(0.05)	(0.05)	(0.05)	(0.05)	(0.05)	(0.05)
Participated x incentives cond.	0.00	0.00	0.06	-0.00	0.01	0.00	0.04	0.09
	(0.07)	(0.08)	(0.07)	(0.07)	(0.07)	(0.07)	(0.07)	(0.07)
Incentives condition	-0.02	-0.01	-0.05	0.04	0.04	0.05	-0.00	-0.05
	(0.07)	(0.07)	(0.06)	(0.06)	(0.06)	(0.07)	(0.06)	(0.06)
Observations	3980	3980	3980	3980	3980	3980	3980	3980

Note: The table shows coefficient estimates from linear regressions of the standardized characteristic variable on an indicator for the incentives condition, an indicator for follow-up survey participation, and the interaction of the two. Heteroscedasticity robust standard errors are shown in parentheses.
* $p < 0.10$, ** $p < 0.05$, *** $p < 0.01$

Table S42: Survey participation based on sociodemographics

Dependent Variable	Age	Female	White	Income	College	Republican
	(1)	(2)	(3)	(4)	(5)	(6)
Participated	0.22***	-0.10*	-0.04	0.10*	0.01	-0.04
	(0.05)	(0.05)	(0.05)	(0.05)	(0.05)	(0.06)
Participated x incentives cond.	0.04	0.04	0.03	-0.05	-0.06	-0.00
	(0.07)	(0.08)	(0.07)	(0.08)	(0.08)	(0.08)
Incentives condition	-0.02	-0.04	-0.03	0.02	0.01	-0.05
	(0.06)	(0.07)	(0.07)	(0.07)	(0.07)	(0.07)
Observations	3980	3980	3980	3980	3980	3980

Note: The table shows coefficient estimates from linear regressions of the standardized characteristic on an indicator for the incentives condition, an indicator for follow-up survey participation, and the interaction of the two. Heteroscedasticity robust standard errors are shown in parentheses.
* $p < 0.10$, ** $p < 0.05$, *** $p < 0.01$

2.6.2 Summary statistics

Table S43 below provides descriptive statistics of our sample. The table first reports the summary statistics for our outcome variables. Note that all the presented indices are standardized. All other outcome variables are not standardized in Table S43, but are standardized for all analyses. We then report the proportion randomly assigned to the incentives condition. Finally, we report the summary statistics for the background variables, including participants' sociodemographics and answers to vaccine and COVID-19 specific questions measured *before* the incentives treatment (see SI section 2.6.8). Note that we decided to add ”Additional dose for $20” and ”Trust in state government” after data collection already started. Hence, we miss data for these outcomes for a few participants.

Table S43: Summary statistics

Variable	Mean	SD	Min.	Max.	N
Outcome variables					
Additional dose	0.43	0.50	0.0	1.0	3,062
Additional dose new outbreak	0.66	0.48	0.0	1.0	3,062
Additional dose for $ 20	0.66	0.48	0.0	1.0	2,935
Flu shot intention next season	0.54	0.50	0.0	1.0	3,062
Blood donation intention	0.12	0.33	0.0	1.0	3,062
Perceived safety and efficacy index	0.00	1.00	-2.3	1.3	3,062
Moral and civic responsibility index	0.00	1.00	-1.9	1.3	3,062
Trust in state government	2.65	0.81	1.0	4.0	2,908
Treatment					
Incentives condition	0.50	0.50	0.0	1.0	3,062
Sociodemographics					
Age	36.76	13.54	18.0	93.0	3,062
Female	0.50	0.50	0.0	1.0	3,062
White	0.70	0.46	0.0	1.0	3,062
University education	0.63	0.48	0.0	1.0	3,062
Income	48592	43329	5000	200000	3,062
Republican	0.21	0.41	0.0	1.0	3,062
Democrat	0.41	0.49	0.0	1.0	3,062
Independent	0.38	0.49	0.0	1.0	3,062
Voted for Trump	0.27	0.45	0.0	1.0	3,062
California	0.15	0.35	0.0	1.0	3,062
Florida	0.14	0.35	0.0	1.0	3,062
Illinoise	0.06	0.24	0.0	1.0	3,062
Kentucky	0.03	0.18	0.0	1.0	3,062
Louisiana	0.03	0.16	0.0	1.0	3,062
Michigan	0.07	0.25	0.0	1.0	3,062
Missouri	0.04	0.19	0.0	1.0	3,062
New York	0.11	0.31	0.0	1.0	3,062
North Carolina	0.06	0.23	0.0	1.0	3,062
Ohio	0.10	0.29	0.0	1.0	3,062
Pennsylvania	0.09	0.28	0.0	1.0	3,062
Texas	0.15	0.35	0.0	1.0	3,062
COVID-19 related baseline var.					
Unvaccinated (vaccination status)	0.22	0.41	0.0	1.0	3,062
Ever tested positive	1.72	0.49	1.0	3.0	3,062
Vaccine safety	3.12	0.87	1.0	4.0	3,062
Vaccine efficacy	3.05	0.91	1.0	4.0	3,062
Willingness additional dose	3.49	1.53	1.0	5.0	3,062
Vaccine efficacy additional dose	2.82	1.00	1.0	4.0	3,062
Vaccine cause disease	1.92	1.10	1.0	5.0	3,062
Trust CDC	3.41	1.30	1.0	5.0	3,062

2.6.3 Treatment effects on awareness

To check whether participants in the incentive condition remembered the information that we gave them in the information provision survey, we included two questions at the end of the follow-up survey (see SI Section 3 for the exact wording of the questions). We used two questions to gauge

awareness: We first asked participants whether their states had offered incentives for COVID-19 vaccination. We then briefly described the incentive program implemented in their state and asked them whether they had heard of it.

Participants in the incentives condition indeed remembered the state incentive programs. Figures S25 gives the distribution of the responses to the more general questions and S26 gives the distribution of the responses to the more specific question. Note that 61% of participants in the Incentives condition and 38% of participants in the Control condition were aware that their states offered incentives for COVID-19 vaccination (that is, they answered ”Definitively yes” or ”Probably yes”). When we look at the awareness about the specific state incentive program implemented in their states, 62% of participants in the Incentives condition and 30% of participants in the Control condition report that they where aware of it. Figure S27 shows that, on average, participants in the incentives condition were 0.50 standard deviations more likely to say that their government offered incentives ($p < 0.001$) and about 0.68 standard deviations more likely to say that they heard of the specific incentive program ($p < 0.001$).

Figure S25: Distribution awareness of state incentive programs

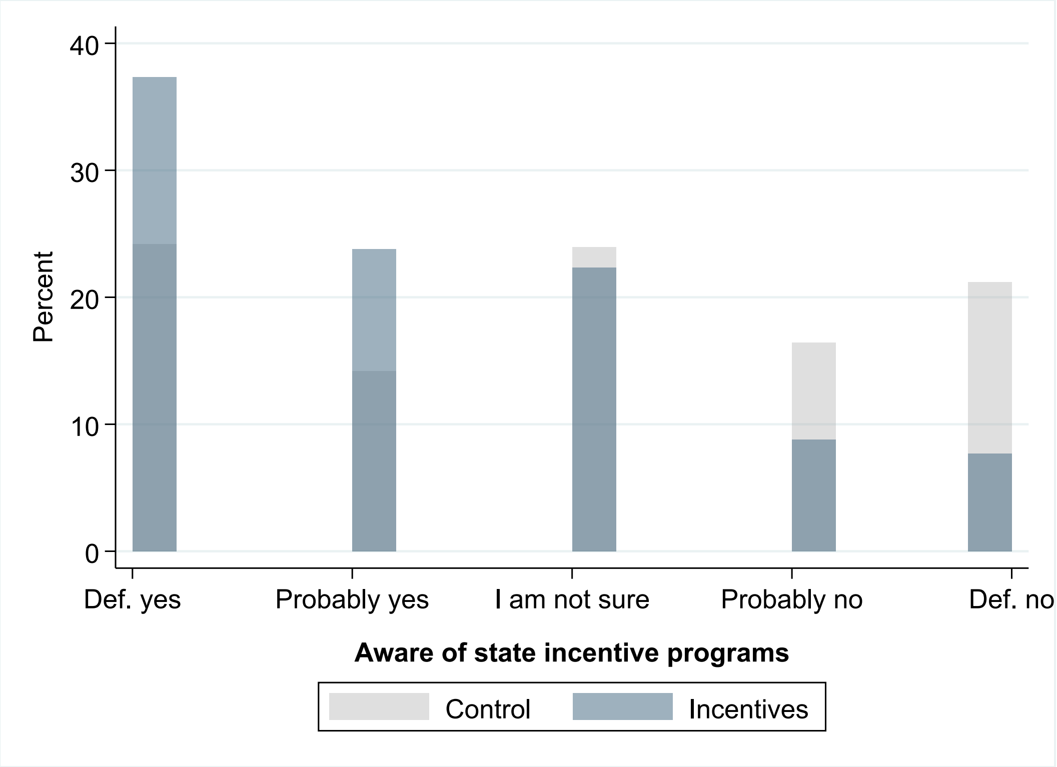

Figure S26: Distribution awareness of specific state incentive program

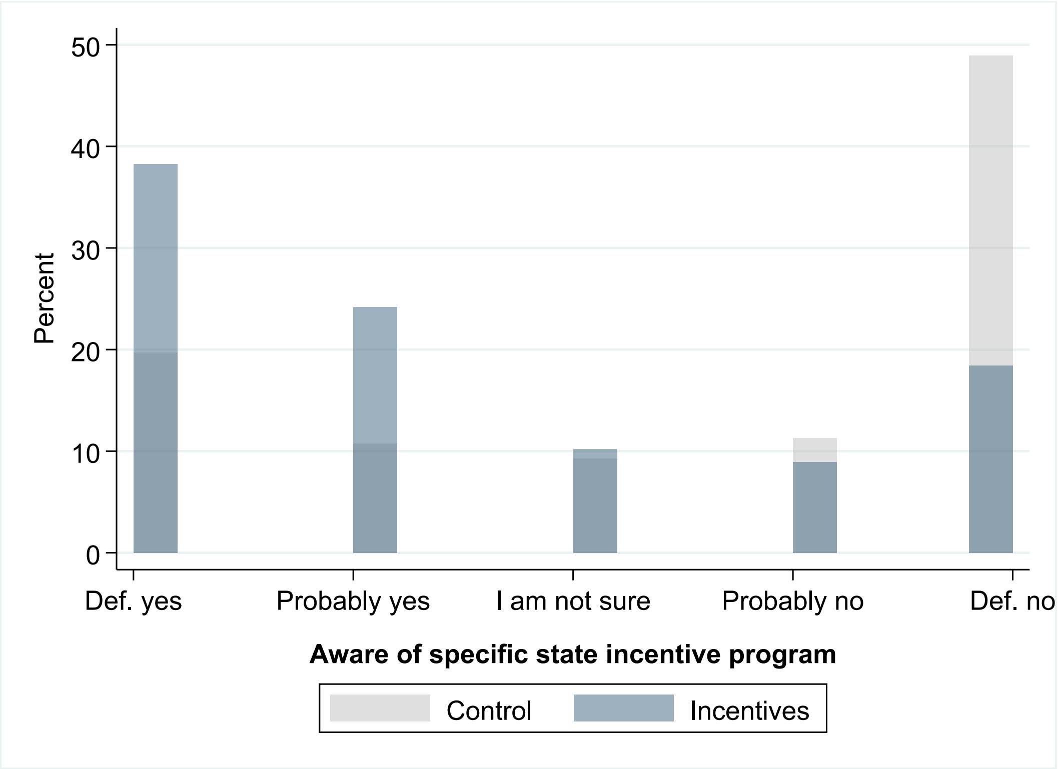

Figure S27: Treatment effects on awareness

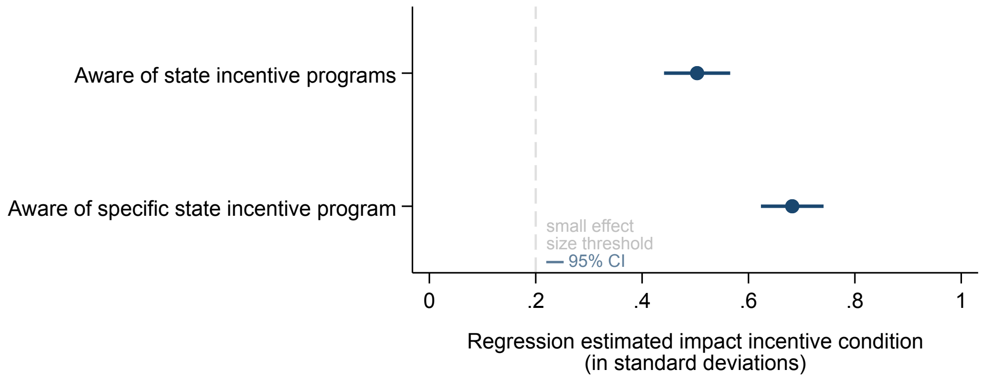

2.6.4 Treatment effects

The following figures and tables report the main treatment effects on a number of health behaviors and perceptions of the incentives condition (i.e., providing participants with information about the incentive program implemented in their state). The variables "Flu shot uptake next season", "Blood donation intention", "Moral and civic responsibility index" and "Perceived safety and efficacy index" are defined exactly as in the Swedish study (see SI Section 3 for the exact wording of the questions, which was slightly adapted to fit the US setting in June 2022). "Additional dose" captures participants' intention to take an additional COVID-19 vaccine dose within the next 6 months. "Additional dose new outbreak" captures participants' intention to take an additional COVID-19 vaccine dose if there would be a next outbreak within the next 6 months and the government would recommend taking the shot. "Additional dose for $20" captures participants' intention to take an additional COVID-19 vaccine dose *if* their government would pay everyone who took it $20 .

The following figure and tables show that there were no meaningful differences in the outcome variables between participants in the incentives and control group.

Figure S28: Treatment effects US study

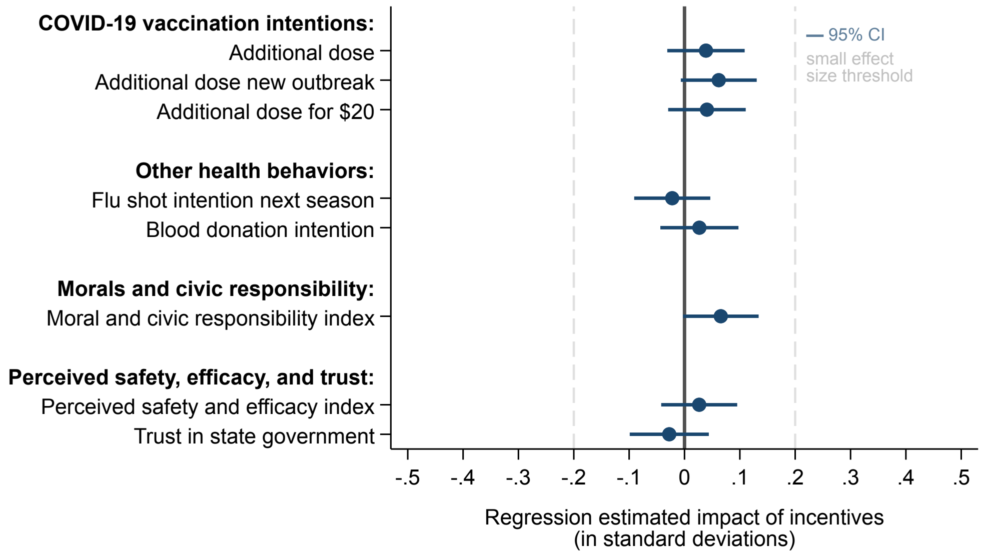

Table S44: Treatment effects US study (COVID-19 vaccination)

Dependent Variable	Additional dose	Add. dose new outbreak	Add. dose for USD 20
	(1)	(2)	(3)
Incentives condition	0.04	0.06*	0.04
	(0.04)	(0.03)	(0.04)
Controls	yes	yes	yes
Observations	3062	3062	2935

Note: The table shows coefficient estimates from linear regressions of the standardized outcome on an indicator for the financial incentives condition. Heteroscedasticity robust standard errors are shown in parentheses. All regressions control for sociodemographics (gender, age, ethnicity, education, employment status and income) and state fixed effects.
* $p < 0.10$, ** $p < 0.05$, *** $p < 0.01$

Table S45: Treatment effects US study (Other health behavior)

Dependent Variable	Flu shot intention next season	Blood donation intention
	(1)	(2)
Incentives condition	-0.02	0.03
	(0.04)	(0.04)
Controls	yes	yes
Observations	3062	3062

Note: The table shows coefficient estimates from linear regressions of the standardized outcome on an indicator for the financial incentives condition. Heteroscedasticity robust standard errors are shown in parentheses. All regressions control for sociodemographics (gender, age, ethnicity, education, employment status and income) and state fixed effects.
* $p < 0.10$, ** $p < 0.05$, *** $p < 0.01$

Table S46: Treatment effects US study (Perceived safety and morals)

Dependent Variable	Safety and efficacy	Moral and civic responsibility	Trust in state government
	(1)	(2)	(3)
Incentives condition	0.03	0.07*	-0.03
	(0.04)	(0.03)	(0.04)
Controls	yes	yes	yes
Observations	3062	3062	2908

Note: The table shows coefficient estimates from linear regressions of the standardized outcome on an indicator for the financial incentives condition. Heteroscedasticity robust standard errors are shown in parentheses. All regressions control for sociodemographics (gender, age, ethnicity, education, employment status and income) and state fixed effects.
* $p < 0.10$, ** $p < 0.05$, *** $p < 0.01$

2.6.5 Equivalence testing

In this section we use equivalence tests to study whether we can reject meaningful negative impacts of financial incentives (*54*, *55*). For each outcome, we test the null hypothesis that the estimated effect size is more negative than a small negative effect benchmark.

Equivalence tests require researchers to specify the smallest effect size of interest (SESOI) as a benchmark (see discussion in SI section 2.2.4). We specify the SESOI as 0.20, which corresponds both to i) the minimum effect size that the study design has power to detect (*55*), as stated in our pre-registration plan, and ii) the definition of a small effect size of 0.2 standard deviations based on Cohen's d (*76*). Accordingly, the null hypothesis (H0) we test against is that the effect size is more negative than -0.2 standard deviations, and the alternative hypothesis (H1) is that the effect size is less negative than -0.2 standard deviations. In practice, this means that we do a one-sided t-test of each estimated effect size being less negative than the SESOI of -0.2 (*55*).

Table S47 provides the results of these equivalence tests. We can clearly reject impacts more negative than -0.2 standard deviations (H0) for each outcome. All tests are highly statistically significant, with the largest p-value among all outcomes being smaller than 0.0001. Hence, for each outcome, we can rule out even a small effect of -0.2 standard deviations. We conclude that there is strong evidence for the absence of even small negative effects.

Table S47: Equivalence testing

Outcome	P-values equivalence testing SESOI = -0.2
COVID-19 vaccination:	
Additional dose	0.0000
Additional dose new outbreak	0.0000
Additional dose for $20	0.0000
Other health behaviors:	
Flu shot intention next season	0.0000
Blood donation intention	0.0000
Moral and civic responsibility:	
Moral and civic responsibility index	0.0000
Perceived safety, efficacy, and trust:	
Perceived safety and efficacy index	0.0000
Trust in state government	0.0000

2.6.6 Different sets of controls

Figures S29 to S32: Treatment effects including different sets of controls. The following figures replicate the main results described above using different sets of controls. The control variables used in each specification are described in the title of each figure.

Figure S29: Different controls: no controls

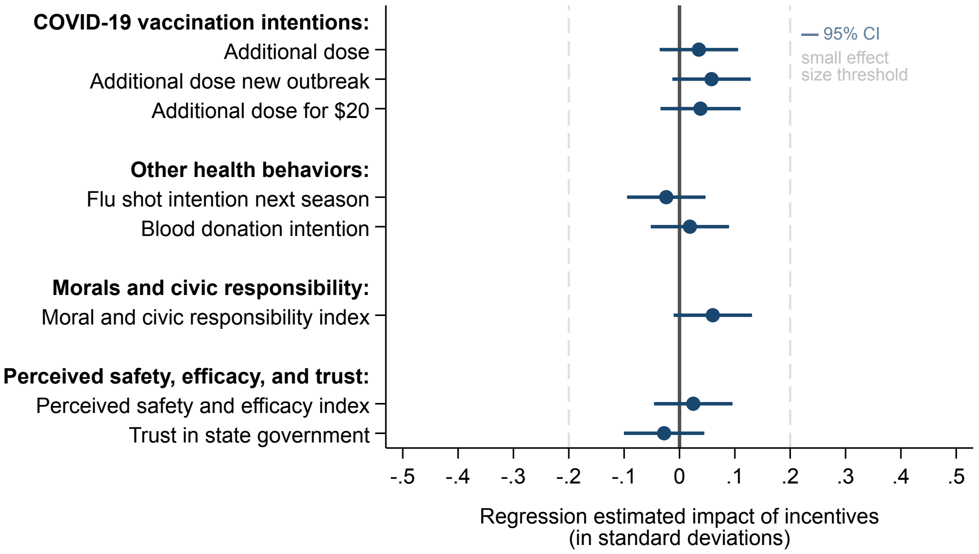

Figure S30: Different controls: controlling for state fixed effects

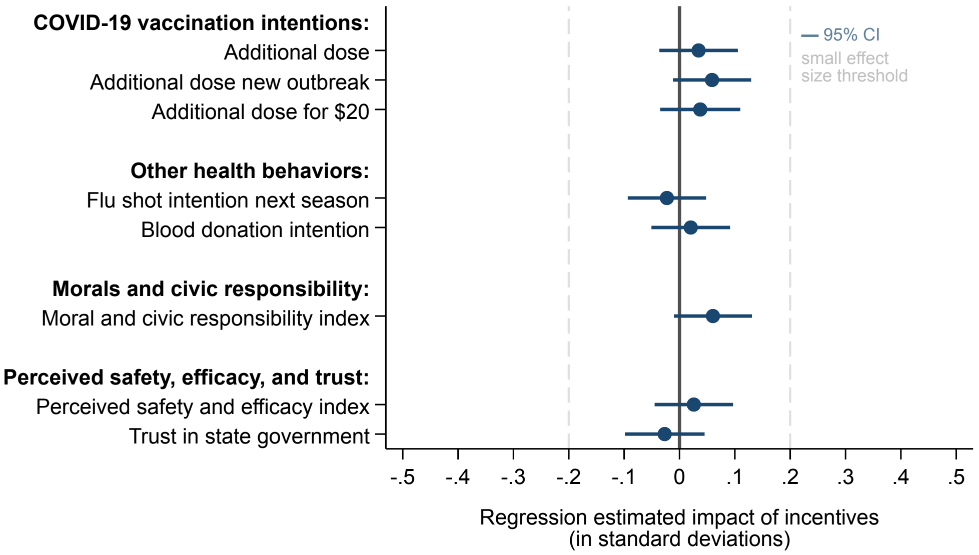

Figure S31: Different controls: controlling for state fixed effects, age, gender and ethnicity

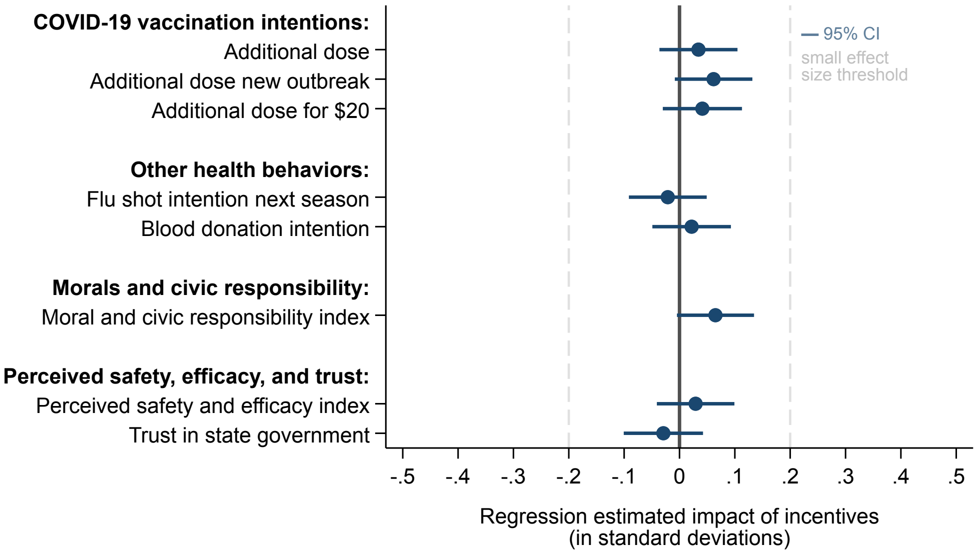

Figure S32: Different controls: controlling for state fixed effects, age, gender, ethnicity, education and employment status

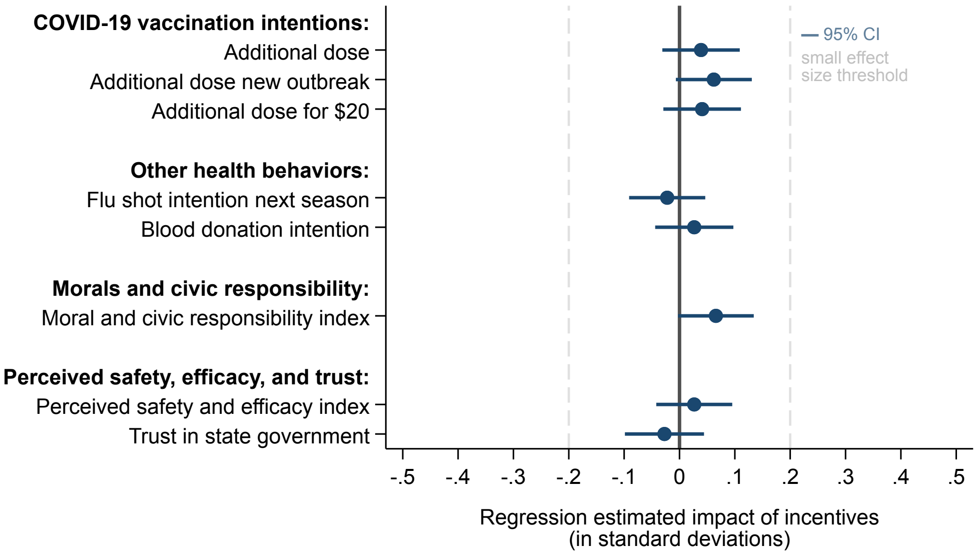

2.6.7 Treatment effects for individuals that were not aware of US state incentive programs.

We elicited two measures of awareness of state incentive programs in the information provision survey (see SI section 3). First, before we allocated participants to the incentives and the control conditions, we asked them whether, in 2021, any governmental organization in their state offered any financial compensation to people who got vaccinated against COVID-19 (”Awareness of state incentive programs”). Second, after we described the incentive program to the participants in the incentives condition, we asked them whether they had heard about this program before (”Awareness of specific state incentive programs”). We find that only a minority of participants were aware of the state incentive programs; 44.81% were aware that any governmental institutions in their state offered any financial compensation and 37.08% said that they had definitively or probably heard about the specific state incentive program.

Table S48 provides regression results on which types of participants were more or less aware that their government offered incentives for vaccination. On average, females, non-whites, and vaccinated participants were more aware of the incentive programs.

Table S49 provides regression results on which types of participants were more or less aware of the specific state incentive programs implemented in their state. Not surprisingly, we find similar results to the previous table, with the exception that the unvaccinated were no longer less aware and that people with higher income were now more aware.

Finally, Figure S33 shows the treatment effects for those participants who were initially not aware that their government offered incentives for vaccination. These results further strengthen the main conclusions that there were no unintended negative consequences of the incentive programs implemented in the US.

Table S48: Awareness of state incentive programs among different groups

	(1)	(2)	(3)	(4)	(5)	(6)	(7)	(8)
Age	-0.003***							-0.003***
	(0.00)							(0.00)
Female		0.072***						0.088***
		(0.02)						(0.02)
White			-0.098***					-0.086***
			(0.02)					(0.02)
Income				0.006*				0.010***
				(0.00)				(0.00)
University education					0.029*			0.006
					(0.02)			(0.02)
Republican						-0.010		0.027
						(0.02)		(0.02)
Unvaccinated							-0.072***	-0.067***
							(0.02)	(0.02)
State FE	yes	yes	yes	yes	yes	yes	yes	yes
Observations	3062	3062	3062	3062	3062	3062	3062	3062

Note: The table shows coefficient estimates from linear regressions of an indictator for being aware that the state of residence in 2021 implemented COVID-19 incentive programs on sociodemographics. All regressions controls for state fixed effects.
* $p < 0.10$, ** $p < 0.05$, *** $p < 0.01$

Table S49: Awareness of specific state incentive programs among different groups

	(1)	(2)	(3)	(4)	(5)	(6)	(7)	(8)
Age	0.001							0.000
	(0.00)							(0.00)
Female		0.054**						0.063***
		(0.02)						(0.02)
White			0.002					-0.005
			(0.03)					(0.03)
Income				0.009**				0.009**
				(0.00)				(0.00)
University education					0.050**			0.025
					(0.02)			(0.02)
Republican						-0.025		-0.028
						(0.03)		(0.03)
Unvaccinated							-0.036	-0.021
							(0.03)	(0.03)
State FE	yes	yes	yes	yes	yes	yes	yes	yes
Observations	1521	1521	1521	1521	1521	1521	1521	1521

Note: The table shows coefficient estimates from linear regressions of an indictator for being aware of the specific state COVID-19 incentive program used in the study on sociodemographics. All regressions controls for state fixed effects.
* $p < 0.10$, ** $p < 0.05$, *** $p < 0.01$

Figure S33: Focusing on individuals that were not aware of the state incentive programs

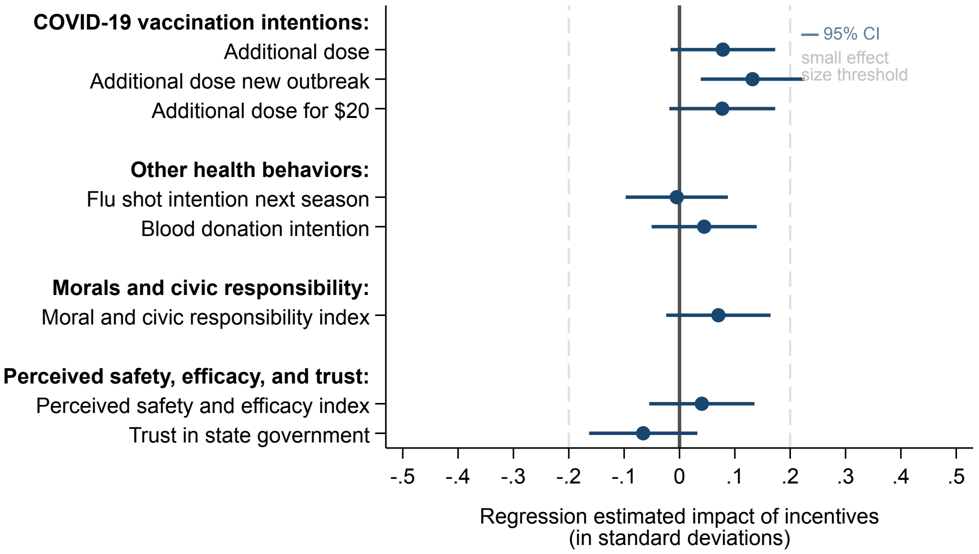

2.6.8 Treatment effects based on participants' characteristics

This section explores potential heterogeneous impacts of the incentives condition on the outcomes based on participants' characteristics. We do heterogeneity analyses based on participants' sociodemographics and vaccination attitudes. Importantly, all characteristics were measured before assignment to the incentives or control condition.

Summary of results: We do not find any robust heterogeneities across 208 coefficient estimates. Despite the large number of coefficients tested, only 3 coefficients are statistically significant negative at the 5% level and only 1 coefficient is statistically significant negative at the 1% level. Hence, there is no indication that there were consistent heterogeneities based on background characteristics.

Variable definitions: We consider gender, age, ethnicity, education, income and political position. Moreover, we consider the following measures of vaccine hesitancy:

- **Vaccination status**: We asked participants how many COVID-19 vaccine doses they have taken. We classified participants into unvaccinated (0 dose) and vaccinated (otherwise).

- **Vaccine safety**: We asked participants whether they think that the first two doses of the currently approved COVID-19 vaccines are safe (scale from 1 to 4).
- **Vaccine efficacy**: We asked participants how effective they think the first two doses of the currently approved COVID-19 vaccines are in terms of reducing the risk of becoming seriously ill from COVID-19 (scale from 1 to 4).
- **Willingness additional dose**: We asked participants how willing they would be to take another COVID-19 vaccine shot within the next year if it was recommended to them to do so (scale from 1 to 5).
- **Vaccine efficacy additional dose**: We asked participants how effective they think it would be for them to take an additional shot of a COVID-19 vaccine (scale from 1 to 4).
- **Vaccine cause disease**: We asked participants whether they agree with the statement that "diseases like autism, multiple sclerosis, and diabetes might be triggered through vaccination" (scale from 1 to 5)
- **Trust CDC**: We asked participants whether they trust the US Center for Disease Control when it comes to the COVID-19 vaccine (scale from 1 to 5).

We provide results on heterogeneities across vaccine attitudes and sociodemographics. To this end, we split the continuous variables (age, income, vaccine safety, vaccine efficacy, willingness additional dose, vaccine efficacy additional dose, vaccine cause disease, trust CDC) based on median values.

Specification: We use an OLS model where we interact an indicator for the incentives condition with an indicator capturing whether participant's characteristic is above or below the median (or, for the binary variables, whether the variable has a value of 0 or 1). We use the following specification:

$$Outcome_i = b_0 + b_1 * 1(Above\_median)_i + b_2 * 1(incentives\_condition)_i * 1(Above\_median)_i$$
$$+ \text{b}_3 * 1(incentives\_condition)_i * 1(Below\_median)_i + b_4 * X_i + e_i$$

where $1(incentives\_condition)_i$ has a value of 1 if participant i is in the incentives condition and a value of 0 otherwise, $1(Above\_median)_i$ has a value of 1 if the participant is above the median of a certain sociodemographic characteristic or COVID-19 vaccination attitude, and $1(Below\_median)_i$ has a value of 1 if the participant is below the corresponding median.

The coefficients for the interactions between incentives condition and these indicator variables (b_2 and b_3) give the *total* impact of the incentives condition on the participant group with below median values and separately for the participant group with above median values. That is, the interaction effect can be interpreted as the total effect of being in the incentives condition given the participant characteristic.

X_i is the vector of control variables used in our main specification and e_i is an individual specific error robust to heteroscedasticity. We indicate the interaction for participants with above median values of a characteristic with "high" (e.g., high age is above median age) and for participants with below median values of a characteristic as "low" (e.g., low age is below median age).

Note that the heterogeneous treatment effects, when added up, may slightly deviate from the average treatment effects reported in the main text. This is because, when we estimate the heterogeneous treatment effects, by construction we have to add additional variables as controls (the ones for which we check whether there are heterogeneous treatment effects). Hence, in some cases the set of controls differs between the main analysis and the analysis of heterogeneous treatment effects which leads to minor differences when computing the average treatment based on the heterogeneity analyses.

Table S50: Heterogeneous treatment effects based on vaccine attitudes

Measure:	Outcomes				
	Add. dose	Add. dose new outbreak	Add. dose for USD 20	Flu shot intention next season	Blood donation intention
Vaccination status:					
Vaccinated x incentives cond.	0.03	0.06*	0.02	-0.04	0.05
	(0.04)	(0.03)	(0.03)	(0.04)	(0.04)
Unvaccinated x incentives cond.	0.04	0.01	0.01	-0.00	-0.05
	(0.04)	(0.05)	(0.05)	(0.05)	(0.07)
Vaccine safety:					
Safe x incentives cond.	0.08	0.06*	0.03	-0.06	0.05
	(0.06)	(0.03)	(0.03)	(0.05)	(0.06)
Not safe x incentives cond.	-0.00	0.05	0.03	-0.01	0.01
	(0.04)	(0.05)	(0.05)	(0.04)	(0.04)
Vaccine efficacy:					
Effective x incentives cond.	0.02	0.07**	0.03	-0.05	0.11*
	(0.06)	(0.03)	(0.03)	(0.05)	(0.06)
Not effective x incentives cond.	0.03	0.04	0.01	-0.02	-0.02
	(0.04)	(0.05)	(0.05)	(0.04)	(0.05)
Willingness add. dose:					
Willing x incentives cond.:	0.05	0.03	-0.01	-0.05	0.08
	(0.05)	(0.02)	(0.02)	(0.05)	(0.06)
Unwilling x incentives cond.:	-0.00	0.05	0.04	-0.04	-0.00
	(0.04)	(0.05)	(0.05)	(0.04)	(0.05)
Vaccine efficacy add. shot:					
Effective x incentives cond.	0.04	0.04	0.01	-0.05	0.04
	(0.06)	(0.03)	(0.03)	(0.06)	(0.07)
Not effective x incentives cond.	0.03	0.07	0.05	-0.01	0.02
	(0.04)	(0.04)	(0.04)	(0.04)	(0.04)
Vacc. cause disease:					
Not cause disease x incentives cond.:	0.02	0.03	0.03	-0.02	0.02
	(0.06)	(0.07)	(0.07)	(0.06)	(0.07)
Cause disease x incentives cond.:	0.05	0.09**	0.06	-0.01	0.03
	(0.04)	(0.04)	(0.04)	(0.04)	(0.04)
Trust CDC:					
High trust x incentives cond.	0.11	0.06	0.03	-0.15**	0.07
	(0.07)	(0.04)	(0.04)	(0.06)	(0.08)
Low trust x incentives cond.	0.03	0.08*	0.06	0.02	0.02
	(0.04)	(0.04)	(0.04)	(0.04)	(0.04)
Controls	yes	yes	yes	yes	yes

Note: The table shows coefficient estimates from linear regressions of the standardized outcome on an indicator for the incentives condition interacted with an indicator capturing a participant's characteristics. The corresponding coefficients indicate the total effect for each subgroup. Heteroscedasticity robust standard errors are shown in parentheses. All regressions controls for age, gender, income, employment status, education and residence state.
* $p < 0.10$, ** $p < 0.05$, *** $p < 0.01$

Table S51: Heterogeneous treatment effects based on vaccine attitudes

Measure:	Outcomes		
	Perceived safety and efficacy index	Trust in state government	Moral and civic responsibility index
Vaccination status:			
Vaccinated x incentives cond.	0.01	0.02	0.06*
	(0.03)	(0.04)	(0.03)
Unvaccinated x incentives cond.	0.03	-0.17**	0.03
	(0.06)	(0.08)	(0.05)
Vaccine safety:			
Safe x incentives cond.	0.00	0.01	0.03
	(0.03)	(0.06)	(0.04)
Not safe x incentives cond.	0.02	-0.05	0.07
	(0.04)	(0.05)	(0.04)
Vaccine efficacy:			
Effective x incentives cond.	0.02	-0.04	0.06
	(0.03)	(0.06)	(0.04)
Not effective x incentives cond.	0.00	-0.01	0.04
	(0.04)	(0.05)	(0.04)
Willingness add. dose:			
Willing x incentives cond.:	0.00	-0.01	0.02
	(0.03)	(0.06)	(0.03)
Unwilling x incentives cond.:	-0.00	-0.03	0.05
	(0.04)	(0.05)	(0.04)
Vaccine efficacy add. shot:			
Effective x incentives cond.	0.02	-0.07	0.05
	(0.04)	(0.07)	(0.04)
Not effective x incentives cond.	0.02	-0.01	0.06
	(0.04)	(0.04)	(0.04)
Vacc. cause disease:			
Not cause disease x incentives cond.:	0.02	-0.09	0.08
	(0.06)	(0.07)	(0.06)
Cause disease x incentives cond.:	0.05	-0.01	0.08**
	(0.03)	(0.04)	(0.04)
Trust CDC:			
High trust x incentives cond.	0.01	-0.05	0.04
	(0.04)	(0.08)	(0.05)
Low trust x incentives cond.	0.05	-0.03	0.09**
	(0.04)	(0.04)	(0.04)
Controls	yes	yes	yes

Note: The table shows coefficient estimates from linear regressions of the standardized outcome on an indicator for the incentives condition interacted with an indicator capturing a participant's characteristics. The corresponding coefficients indicate the total effect for each subgroup. Heteroscedasticity robust standard errors are shown in parentheses. All regressions controls for age, gender, income, employment status, education and residence state.
* $p < 0.10$, ** $p < 0.05$, *** $p < 0.01$

Table S52: Heterogeneous treatment effects based on sociodemographics

Measure:	Outcomes				
	Add. dose	Add. dose new outbreak	Add. dose for USD 20	Flu shot intention next season	Blood donation intention
Age:					
High age x incentives cond.	0.06	0.05	-0.01	0.01	0.04
	(0.05)	(0.05)	(0.05)	(0.05)	(0.05)
Low age x incentives cond.	0.02	0.07	0.09*	-0.05	0.01
	(0.05)	(0.05)	(0.05)	(0.05)	(0.05)
Income:					
High income x incentives cond.	0.09*	0.09*	0.04	-0.01	0.03
	(0.05)	(0.05)	(0.05)	(0.05)	(0.06)
Low income x incentives cond.	-0.00	0.04	0.04	-0.03	0.02
	(0.05)	(0.05)	(0.05)	(0.05)	(0.05)
Gender:					
Female x incentives cond.	0.06	0.10**	0.09*	0.01	0.07
	(0.05)	(0.05)	(0.05)	(0.05)	(0.05)
Male x incentives cond.	0.02	0.03	-0.01	-0.06	-0.02
	(0.05)	(0.05)	(0.05)	(0.05)	(0.05)
White:					
White x incentives cond.	0.09**	0.06	0.02	0.00	0.07
	(0.04)	(0.04)	(0.04)	(0.04)	(0.04)
Non-White x incentives cond.	-0.09	0.06	0.07	-0.09	-0.07
	(0.07)	(0.06)	(0.06)	(0.06)	(0.06)
College:					
College x incentives cond.	0.05	0.11***	0.08*	0.02	0.03
	(0.05)	(0.04)	(0.04)	(0.04)	(0.05)
No college degree x incentives cond.	0.02	-0.02	-0.02	-0.09	0.03
	(0.06)	(0.06)	(0.06)	(0.06)	(0.06)
Political position:					
Republican x incentives cond.	0.06	0.08	0.02	0.03	0.01
	(0.07)	(0.08)	(0.08)	(0.07)	(0.08)
Not Republican x incentives cond.	0.02	0.04	0.03	-0.05	0.03
	(0.04)	(0.04)	(0.04)	(0.04)	(0.04)
Controls	yes	yes	yes	yes	yes

Note: The table shows coefficient estimates from linear regressions of the standardized outcome on an indicator for the incentives condition interacted with an indicator capturing a participant's characteristics. The corresponding coefficients indicate the total effect for each subgroup. Heteroscedasticity robust standard errors are shown in parentheses. All regressions controls for age, gender, income, employment status, education and residence state.
* $p < 0.10$, ** $p < 0.05$, *** $p < 0.01$

Table S53: Heterogeneous treatment effects based on sociodemographics

Measure:	Outcomes		
	Perceived safety and efficacy index	Trust in state government	Moral and civic responsibility index
Age:			
High age x incentives cond.	-0.01	0.08	0.05
	(0.05)	(0.06)	(0.05)
Low age x incentives cond.	0.06	-0.13***	0.08*
	(0.05)	(0.05)	(0.05)
Income:			
High income x incentives cond.	0.05	-0.01	0.14***
	(0.05)	(0.05)	(0.05)
Low income x incentives cond.	0.01	-0.04	0.00
	(0.05)	(0.05)	(0.05)
Gender:			
Female x incentives cond.	0.08	-0.11**	0.09*
	(0.05)	(0.05)	(0.05)
Male x incentives cond.	-0.02	0.05	0.04
	(0.05)	(0.05)	(0.05)
White:			
White x incentives cond.	0.03	-0.01	0.08*
	(0.04)	(0.04)	(0.04)
Non-White x incentives cond.	0.01	-0.06	0.04
	(0.06)	(0.07)	(0.06)
College:			
College x incentives cond.	0.05	-0.04	0.11**
	(0.04)	(0.05)	(0.04)
No college degree x incentives cond.	-0.00	-0.01	0.00
	(0.06)	(0.06)	(0.06)
Political position:			
Republican x incentives cond.	0.00	-0.07	0.11
	(0.08)	(0.09)	(0.07)
Not Republican x incentives cond.	0.01	-0.02	0.03
	(0.04)	(0.04)	(0.04)
Controls	yes	yes	yes

Note: The table shows coefficient estimates from linear regressions of the standardized outcome on an indicator for the incentives condition interacted with an indicator capturing a participant's characteristics. The corresponding coefficients indicate the total effect for each subgroup. Heteroscedasticity robust standard errors are shown in parentheses. All regressions controls for age, gender, income, employment status, education and residence state.
* $p < 0.10$, ** $p < 0.05$, *** $p < 0.01$

2.6.9 Treatment effects based on state

This section explores potential heterogeneous impacts of the incentives condition on the outcomes based on participants' state of residence in 2021. The participants that were randomly assigned to the incentives condition received detailed information about their state's COVID-19 vaccine incentive program. These state incentive programs differed substantially across states (see SI section 1.2.2). Hence, treatment effects could differ across states.

One reason for potential differences in effects could be the success of state incentive programs in fostering vaccination. The evidence on whether state-offered lottery incentives increased vacci-

nation rates in the US is mixed (*58-63*). There is some evidence that in some states the incentive programs (including both, guaranteed incentives and lotteries) may have been more successful than in others in increasing vaccination. For example, Acharya and Dhakal (*60*) found positive effects in Ohio, Oregon, Washington, New Mexico and New York, but no effects in Arkansas, Kentucky, and West Virginia. Some states offered guaranteed incentives for vaccination; evidence suggests that guaranteed incentives may have been more successful in increasing vaccination than lottery incentives (*59*).

The theoretical literature predicts unintended consequences even in the absence of positive effects on vaccination. For example, individuals may not be more likely to vaccinate in response to incentives, but may grow more suspicious of vaccinations or change their morals around vaccination. However, the unintended consequences of the incentives could still depend on the success of the program.

We collected data from states in which there is some evidence that the lottery incentive programs were more successful (Ohio, New York, Kentucky) and states that offered guaranteed cash payments (California, Florida, Louisiana, Pennsylvania, see Table S1), which allows us to probe the question whether there were unintended consequences in states where incentive programs were more or less successful.

Summary of results: We do not find any robust evidence that state incentive programs had negative unintended consequences in any of the 12 states. No coefficient is statistically significantly negative at the 5% level. Hence, there is no indication that there were heterogeneities based on state of residence. We also do not find any evidence for negative unintended consequences when we focus on states in which there is some evidence that the incentive programs were more successful (Ohio, New York, California, Florida, Louisiana, Pennsylvania) or states in which the incentive programs were less successful (Kentucky).

Specification: We use an OLS model where we interact an indicator for the incentives condition with indicators capturing the state of residency of the participant. We use the following specification:

$$Outcome_i = b_1 * 1(California)_i + ... + b_{12} * 1(Texas)_i + b_{13} * 1(incentives\_condition)_i * 1(California)_i + ... + b_{24} * 1(incentives\_condition)_i * 1(Texas)_i + b_{25} * X_i + e_i$$

where $1(incentives\_condition)_i$ has a value of 1 if participant i is in the incentives condition and a value of 0 otherwise and $1(State)_i$ has a value of 1 if the participant resided in state $State$

in 2021. X_i is the vector of control variables used in our main specification and e_i is an individual specific error robust to heteroscedasticity.

The coefficients for the interactions between incentives condition and the state indicator variables (b_{13} to b_{24}) give the *total* impact of the incentives condition for each state. Table S54 and S55 give the estimates for these state treatment effects.

To study whether there are unintended consequences in states where incentive programs have been more or less successful, we pool data from states in which there is some evidence that lottery incentive programs were more successful (Ohio and New York; see *60*, *61*, *63*) and states in which the incentive programs were less successful (Kentucky; see *60*). We use the following specification:

$$Outcome_i = b_1 * 1(Ohio\ or\ New\ York)_i + b_2 * 1(Kentucky)_i + b_3 * 1(Other\ state)_i + b_4 * 1(incentives\_condition)_i * 1(Ohio\ or\ NewYork)_i + b_5 * 1(incentives\_condition)_i * 1(Kentucky)_i + b_6 * 1(incentives\_condition)_i * 1(Other\ state)_i * 1(incentives\_condition)_i + b_7 * X_i + e_i$$

where $1(Ohio\ or\ New\ York)_i$ has a value of 1 if the participant resided either in Ohio or New York in 2021, $1(Kentucky)_i$ has a value of 1 if the participant resided either in Kentucky or Pennsylvania in 2021 and $1(Other\ state)_i$ has a value of 1 if the participant resided in any of the other states in 2021.

The coefficients for the interactions between incentives condition and the state indicator variables (b_4 to b_6) give the *total* impact of the incentives condition for each state. Table S56 and S57 give the estimates for these state treatment effects.

To further study whether there are unintended consequences in states where incentive programs have been more or less successful, we can also pool data from states that offered guaranteed incentives (California, Florida, Louisiana, Pennsylvania), which have arguably been more successful in increasing vaccination rates than lottery incentives (*59*). We use the following specification:

$$Outcome_i = b_1 * 1(Guaranteed)_i + b_2 * 1(Lottery)_i + b_3 * 1(incentives\_condition)_i * 1(Guaranteed)_i + b_4 * 1(incentives\_condition)_i * 1(Lottery)_i + b_5 * X_i + e_i$$

where $1(Guaranteed)_i$ has a value of 1 if the participant resided in a state that offered guaranteed cash incentives in 2021 (or a state that offered both guaranteed and lottery incentives), $1(Lottery)_i$ has a value of 1 if the participant resided in a state that lottery incentives in 2021.

The coefficients for the interactions between incentives condition and the state indicator variables (b_3 and b_4) give the *total* impact of the incentives condition for each state. Table S58 and S59 give the estimates for these state treatment effects.

Table S54: Heterogeneous treatment effects based on state

Measure:	Outcomes				
	Add. dose	Add. dose new outbreak	Add. dose for USD 20	Flu shot uptake next season	Blood donation intention
State of residence 2021:					
California x incentives	0.11	0.01	0.05	-0.13	-0.14
	(0.09)	(0.09)	(0.09)	(0.09)	(0.09)
Florida x incentives	0.01	0.11	-0.00	0.05	0.11
	(0.09)	(0.10)	(0.10)	(0.10)	(0.10)
Illinois x incentives	0.04	-0.05	-0.00	0.13	-0.15
	(0.15)	(0.14)	(0.14)	(0.14)	(0.16)
Kentucky x incentives	0.01	0.00	-0.05	0.05	-0.14
	(0.20)	(0.20)	(0.20)	(0.20)	(0.20)
Louisiana x incentives	0.11	-0.05	-0.09	0.21	0.40
	(0.22)	(0.22)	(0.22)	(0.20)	(0.27)
Michigan x incentives	0.09	0.07	0.07	-0.17	0.14
	(0.14)	(0.14)	(0.14)	(0.14)	(0.14)
Missouri x incentives	0.02	-0.05	0.01	-0.07	-0.09
	(0.18)	(0.17)	(0.17)	(0.17)	(0.18)
New York x incentives	0.10	0.10	0.10	-0.07	0.23**
	(0.11)	(0.10)	(0.11)	(0.11)	(0.11)
North Carolina x incentives	0.05	0.21	0.20	0.10	0.17
	(0.15)	(0.15)	(0.15)	(0.15)	(0.17)
Ohio x incentives	-0.00	0.23*	0.16	0.09	0.04
	(0.12)	(0.12)	(0.12)	(0.11)	(0.12)
Pennsylvania x incentives	0.21*	0.01	-0.05	0.02	0.14
	(0.12)	(0.12)	(0.12)	(0.12)	(0.12)
Texas x incentives	-0.16*	0.00	-0.00	-0.13	-0.15*
	(0.09)	(0.09)	(0.10)	(0.09)	(0.09)
Controls	yes	yes	yes	yes	yes

Note: The table shows estimated treatment effects for each state. The coefficient estimates are from linear regressions of the standardized outcome on an indicators for the incentives condition interacted with indicators capturing a participant's state of residence in 2021. The corresponding coefficients indicate the total effect for participants residing in this state in 2021. Heteroscedasticity robust standard errors are shown in parentheses. All regressions controls for age, gender, income, employment status, education and residence state.
* $p < 0.10$, ** $p < 0.05$, *** $p < 0.01$

Table S55: Heterogeneous treatment effects based on state

Measure:		Outcomes	
	Perceived safety and efficacy index	Trust in state government	Moral and civic responsibility index
State of residence 2021:			
California x incentives	0.06	-0.08	0.02
	(0.08)	(0.10)	(0.09)
Florida x incentives	0.05	-0.09	0.04
	(0.10)	(0.10)	(0.10)
Illinois x incentives	-0.14	-0.02	-0.04
	(0.14)	(0.15)	(0.14)
Kentucky x incentives	0.07	0.17	0.16
	(0.20)	(0.18)	(0.19)
Louisiana x incentives	-0.06	0.17	-0.12
	(0.23)	(0.23)	(0.21)
Michigan x incentives	-0.03	-0.07	0.02
	(0.14)	(0.13)	(0.14)
Missouri x incentives	-0.06	-0.03	-0.09
	(0.18)	(0.20)	(0.18)
New York x incentives	0.06	0.13	0.20*
	(0.10)	(0.11)	(0.10)
North Carolina x incentives	0.15	-0.14	0.16
	(0.15)	(0.13)	(0.15)
Ohio x incentives	0.32***	-0.11	0.27**
	(0.12)	(0.12)	(0.12)
Pennsylvania x incentives	-0.09	0.17	0.02
	(0.11)	(0.11)	(0.12)
Texas x incentives	-0.10	-0.12	0.02
	(0.09)	(0.10)	(0.09)
Controls	yes	yes	yes

Note: The table shows coefficient estimates from linear regressions of the standardized outcome on an indicator for the incentives condition interacted with an indicator capturing a participant's characteristics. The corresponding coefficients indicate the total effect for each subgroup. Heteroscedasticity robust standard errors are shown in parentheses. All regressions controls for age, gender, income, employment status, education and residence state.
* $p < 0.10$, ** $p < 0.05$, *** $p < 0.01$

Table S56: Heterogeneous treatment effects based on state

Measure:	Outcomes				
	Add. dose	Add. dose new outbreak	Add. dose for USD 20	Flu shot uptake next season	Blood donation intention
State of residence 2021:					
Ohio or New York x incentives	0.05	0.16**	0.13*	0.01	0.14*
	(0.08)	(0.08)	(0.08)	(0.08)	(0.08)
Kentucky x incentives	0.01	0.00	-0.05	0.04	-0.14
	(0.20)	(0.19)	(0.20)	(0.20)	(0.20)
Other states x incentives	0.04	0.04	0.02	-0.04	0.00
	(0.04)	(0.04)	(0.04)	(0.04)	(0.04)
Controls	yes	yes	yes	yes	yes

Note: The table shows estimated treatment effects when we pool data from states in which there is some evidence that the incentive programs were more successful (Ohio and New York) and states in which the incentive programs were less successful (Kentucky and Pennsylvania). The coefficient estimates are from linear regressions of the standardized outcome on an indicators for the incentives condition interacted with indicators capturing a participant's state of residence in 2021. The corresponding coefficients indicate the total effect for participants residing in this state in 2021. Heteroscedasticity robust standard errors are shown in parentheses. All regressions controls for age, gender, income, employment status, education and residence state.
* $p < 0.10$, ** $p < 0.05$, *** $p < 0.01$

Table S57: Heterogeneous treatment effects based on state

Measure:	Outcomes		
	Perceived safety and efficacy index	Trust in state government	Moral and civic responsibility index
State of residence 2021:			
Ohio or New York x incentives	0.18**	0.02	0.23***
	(0.08)	(0.08)	(0.08)
Kentucky x incentives	0.07	0.18	0.16
	(0.20)	(0.18)	(0.19)
Other states x incentives	-0.02	-0.05	0.02
	(0.04)	(0.04)	(0.04)
Controls	yes	yes	yes

Note: The table shows estimated treatment effects when we pool data from states in which there is some evidence that the incentive programs were more successful (Ohio and New York) and states in which the incentive programs were less successful (Kentucky and Pennsylvania). The corresponding coefficients indicate the total effect for each subgroup. Heteroscedasticity robust standard errors are shown in parentheses. All regressions controls for age, gender, income, employment status, education and residence state.
* $p < 0.10$, ** $p < 0.05$, *** $p < 0.01$

Table S58: Heterogeneous treatment effects based on state

Measure:	Outcomes				
	Add. dose	Add. dose new outbreak	Add. dose for USD 20	Flu shot uptake next season	Blood donation intention
State of residence 2021:					
Guaranteed x incentives	0.10*	0.04	0.00	-0.02	0.04
	(0.06)	(0.06)	(0.06)	(0.06)	(0.06)
Lottery x incentives	-0.00	0.07	0.07	-0.03	0.02
	(0.05)	(0.05)	(0.05)	(0.05)	(0.05)
Controls	yes	yes	yes	yes	yes

Note: The table shows estimated treatment effects when we pool data from states that offered guaranteed cash incentives (California, Florida, Lousiana, Pennsylvania) and data from the states that offered lotteries. The coefficient estimates are from linear regressions of the standardized outcome on an indicators for the incentives condition interacted with indicators capturing a participant's state of residence in 2021. The corresponding coefficients indicate the total effect for participants residing in this state in 2021. Heteroscedasticity robust standard errors are shown in parentheses. All regressions controls for age, gender, income, employment status, education and residence state.
* $p < 0.10$, ** $p < 0.05$, *** $p < 0.01$

Table S59: Heterogeneous treatment effects based on state

Measure:	Outcomes		
	Perceived safety and efficacy index	Trust in state government	Moral and civic responsibility index
State of residence 2021:			
Guaranteed x incentives	0.01	-0.01	0.02
	(0.05)	(0.06)	(0.06)
Lottery x incentives	0.03	-0.04	0.10**
	(0.05)	(0.05)	(0.05)
Controls	yes	yes	yes

Note: The table shows estimated treatment effects when we pool data from states that offered guaranteed cash incentives (California, Florida, Lousiana, Pennsylvania) and data from the states that offered lotteries. The corresponding coefficients indicate the total effect for each subgroup. Heteroscedasticity robust standard errors are shown in parentheses. All regressions controls for age, gender, income, employment status, education and residence state.
* $p < 0.10$, ** $p < 0.05$, *** $p < 0.01$

2.6.10 Results for single items of indices

Figure S34: Treatment effects on each single item of the indices. Figure S34 shows all coefficient estimates for each single (and standardized) item. The figure provides the effect sizes from our main specification for the indices alongside each single item that the respective index is composed of (see the Methods section for details on the composition of each index). The results for single items confirm the main results.

Figure S34: Treatment effects on each single item of the indices

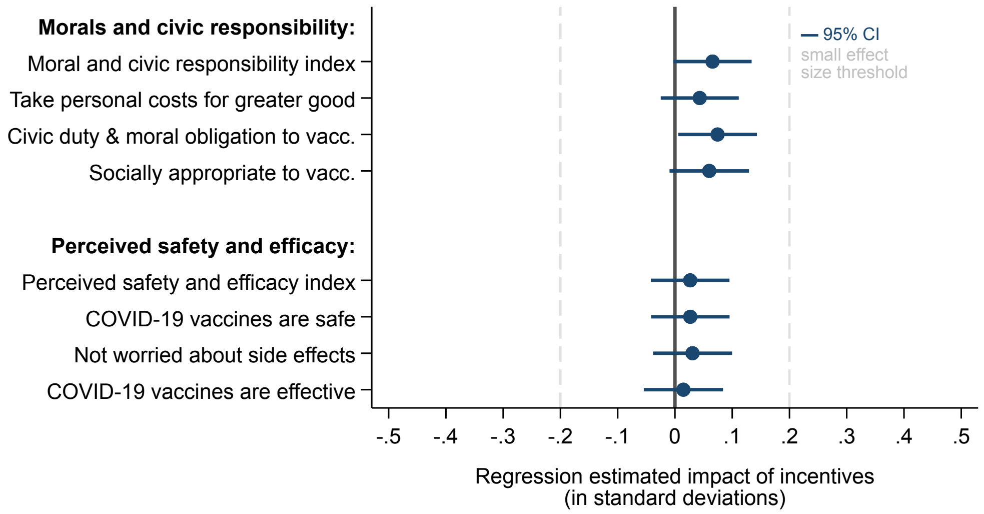

3 Questionnaires

3.1 Surveys Sweden

What follows are the survey questions that all participants filled out. The trial was conducted in Swedish, we include an English translation of the text. For the items used in previous research, whenever possible we used researcher-provided translations of the questions. The consent forms of the first and the second surveys were equivalent (except for the expected duration and reward). After the consent form, we first show the outcome variables from the first study and second we show the outcome variables for the second study.

3.1.1 Consent form

Consent form

We are a group of researchers from Sweden, Denmark, Switzerland and the USA who are conducting a study on preferences, opinions and attitudes related to the coronavirus. Answering the questions in this survey takes about 5 minutes and you will get 50 Norstat coins (SEK 50). By participating in the study, you contribute to research in this area. The principal researcher for the project is Lund University. No special knowledge is required to participate in the study, and your answers will remain confidential and anonymous.

Insurance and compensation

As your participation in the study does not involve any obvious risks, there is no special insurance linked to the study. When you have completed the survey, you will receive SEK 50. The compensation is taxable.

Data management and confidentiality

The project will collect and record information about you and your opinions. Your answers will be protected so that unauthorized persons will not be able to access them. Lund University is responsible for your personal data. Your answers will be linked to information from a previous Norstat study that you participated in regarding your preferences, attitudes and behaviors related to the coronavirus and vaccine, including information about whether you took the vaccine. According to the EU Data Protection Regulation, you have the right to access the information about you that is handled in the study free of charge, and if necessary correct any errors. You can also request that information about you be deleted and that the processing of your personal data be restricted. If you want to access this information, please contact the project manager Erik Wengström, who

can be reached on telephone 046-222 0123 or via email erik.wengstrom@nek.lu.se. The Data Protection Ombudsman at Lund University, Kristin Asgermyr, can be reached on telephone 046-222 0000 or via email dataskyddsombud@lu.se. If you are dissatisfied with how your personal data is processed, you have the right to submit a complaint to the Data Inspectorate/ Privacy Protection Authority, which is the supervisory authority (from 1 January 2021, the Data Inspectorate will instead be called the Privacy Protection Authority). Deidentified data may be shared with other researchers and will be saved permanently so that future researchers can take part in the research and evaluate it.

How can I get information about the project results?

The results of the project will be presented in research reports. The research reports will only contain summary statistics about the participants, i.e. number of participants, proportion of women, age distribution, etc. Research reports can be ordered from the responsible researcher (see below). It usually takes time (more than a year) before there is a complete report. Aggregate information about other participants' decisions can be obtained on request.

How can I save the information on this page

If you want to save the information on this page, please print the page as a pdf file and save the file to your computer. Alternatively, after the study, you can access the information again on the following website: https://sites.google.com/site/erikwengstrom/corona.

Responsible for the study

The person responsible for the study is Professor Erik Wengström, Department of Economics at Lund University. Address: Department of Economics, Box 7080, 220 07 Lund.
Email: erik.wengstrom@nek.lu.se. Phone: 046 222 0123.

Participation

Your participation is voluntary and you can choose to cancel your participation at any time. If you choose not to participate or want to cancel your participation, you do not need to state why. If you wish to cancel your participation, please contact the person responsible for the study (see below).

Consent to participate in the study

I have received information about the study and data management and I agree to participate in the study

3.1.2 Outcome measures first survey

- Are you planning to take a third dose of COVID-19 vaccine (booster shot) when it becomes available to you? If you have already taken a third dose of a COVID-19 vaccine, please select "Yes". (Yes/No)
- From the moment the third dose of COVID-19 vaccine becomes available to you, how long do you think you will wait before taking it? (Less than a month/Between 1 and 2 months/Between 3 and 4 months/More than 4 months/I don't plan to take it)
- Have you been vaccinated against the seasonal flu in the last 5 months? (Yes/No)
- Between 0% and 100%, how likely is it that you will be vaccinated against the seasonal flu in next winter (2022-2023)?
- If you had a newborn child, would you follow the vaccination program for children in Sweden (which includes, for example, vaccinations against measles and polio)? (Absolutely not/Probably not/Maybe/Probably yes/Absolutely yes)
- Have you donated blood in the last 5 months? (Yes/No)
- Do you have a partner? (Yes/No)
- Are your parents alive? (No, none of them/Yes, one of them/Yes, both of them)
- (If the participant has a partner) Has your partner taken a first dose of a COVID-19 vaccine? (Yes/No/I don't know)
- (If the participant has one parent alive) Has your parent taken a first dose of a COVID-19 vaccine? (Yes/No/I don't know)
- (If the participant has two parents alive) Have your parents taken a first dose of a COVID-19 vaccine? (Yes/No/I don't know)
- To what extent do you agree with the following statements (5-points Likert scales):
  - The COVID-19 vaccines are generally safe
  - I'm worried about the effects of the COVID-19 vaccines
  - COVID-19 vaccines provide effective protection for my health

    - Taking a third dose of a COVID-19 vaccine is unnecessary and causes some discomfort
    - In general, vaccines given to children, such as the measles vaccine, are safe for healthy children

- To what extent do you agree with the following statements (5-points Likert scales):

    - I am willing to take the personal expenses associated with taking a COVID-19 vaccine (e.g., time, discomfort, mild side effects) for the benefit of the community
    - I think people have a civic duty or a moral obligation to take a COVID-19 vaccine
    - In general, it is considered socially inappropriate not to be vaccinated against COVID-19
    - When it comes to the COVID-19 vaccines, I trust pharmaceutical companies that develop the vaccines
    - When it comes to the COVID-19 vaccines, I trust the researchers who are studying the effects of the vaccines
    - When it comes to the COVID-19 vaccine, I trust the Public Health Agency of Sweden

- Have you taken a first dose of a COVID-19 vaccine? (Yes/No)

- When you look back at your decision to take, or not take, a first dose of a COVID-19 vaccine, to what extent do you agree with the following statements agree (5-points Likert scales):

    - I regret my decision
    - I gathered enough information to understand the benefits and risks of the vaccine.
    - I felt forced to make the decision I made

- We offer to send you a reminder email when a third dose of a COVID-19 vaccine becomes available to people in your region and age group. The reminder will be sent by Norstat and contain information on how you can make an appointment to receive the vaccine. Do you want to receive the reminder? (Yes/No)

- Do the following statements apply to you? Please note that we will provide summary information on how you participants answer these questions to the Public Health Agency of Sweden and politicians. Given that your opinions may therefore affect health policy, it is very important that you make careful choices (5-points Likert scales):

  - Vaccination against COVID-19 should be mandatory for health care workers
  - It is unethical to offer financial rewards to those who are vaccinated against COVID-19
  - I would support the introduction of a reward of SEK 500 to those who get vaccinated (or have been vaccinated) against COVID-19
  - I would support the introduction of financial rewards for blood donors

- Now the computer will randomly select 10 participants and give them an initial allocation of SEK 100. If you are one of the 10 participants who are awarded SEK 100, how much of the SEK 100 do you want to donate to the Global Alliance for Vaccines and Immunization (GAVI)? GAVI is a non-governmental organization that collects donations to provide COVID-19 vaccines in areas with limited access to vaccines. Donating a COVID-19 vaccine costs SEK 50. Think carefully about your decision. If you are selected as one of the 10 participants, we will give the amount you choose to GAVI and pay you what is left from the SEK 100 in Norstat coins.

  - How much do you want to give to GAVI to provide COVID-19 vaccines? (we pay you the rest)

- Now the computer will randomly select 10 additional participants and give them an initial allocation of SEK 100. If you are one of the 10 participants who are awarded SEK 100, how much of the SEK 100 do you want to donate to the New Incentives Organization (NIO)? NIO is a non-governmental organization that tries to increase the willingness to vaccinate by paying people to get vaccinated. Their only mission is to ”use cash transfers to increase the degree of immunization”. Therefore, all donations sent to this organization are used to pay people to get vaccinated. Think carefully about your decision. If you are selected as one of the 10 participants, we will give the amount you choose to NIO and pay you what is left from the SEK 100 in Norstat coins.

  - How much do you want to give to NIO to pay for vaccination? (we pay you the rest)

- Suppose a third dose of a COVID-19 vaccine is available to you. To increase the uptake of the vaccine, assume that your region pays SEK 100 for everyone who takes the vaccine (including you). Would you take a third dose of a COVID-19 vaccine? (No, absolutely not/No, probably not/Maybe/Yes, probably/Yes, absolutely)

- Suppose a third dose of a COVID-19 vaccine is available to you. To increase the uptake of the vaccine, assume that your region pays SEK 500 for everyone who takes the vaccine (including you). Would you take a third dose of a COVID-19 vaccine? (No, absolutely not/No, probably not/Maybe/Yes, probably/Yes, absolutely)
- Currently, around 85% of all adult Swedes have taken a first dose of a COVID-19 vaccine. Assume that the regions in Sweden in the spring of 2021 had tried to increase the uptake of COVID-19 vaccination by paying people SEK 200 to get vaccinated. What percentage of Swedes do you think would have taken a first dose of COVID-19 vaccine in that case?
- Between 0% and 100%, how likely is it that you will be offered a cash reward by the government or your region for taking a third dose of COVID-19 vaccine?
- Click here for information on when the third dose of a COVID-19 vaccine will be available to you in your region (the link will open in a new window).

3.1.3 Outcome measures second survey

- Have you taken a third dose of a COVID-19 vaccine? (Yes/No)
- (If third dose = Yes) When did you take your third dose of COVID-19 vaccine? (Before January 2022, January 2022, February 2022, March 2022, April 2022, May 2022, June 2022)

3.2 Swedish complementary study

The Swedish complementary study was conducted in Swedish, we include an English translation of the text. For the items used in previous research, whenever possible we used researcher-provided translations of the questions.

3.2.1 Consent Form

Background

We are a group of researchers led by Professor Erik Wengström from Lund University who are conducting a **[TREATMENT SELECTION: study on vaccination against the coronavirus (COVID-19) / CONTROL SELECTION: research study]**. No special knowledge is required to participate in the study, and your answers will remain confidential and anonymous.

Data management and confidentiality

The project will collect and record information about you and your opinions. Your answers will be protected so that unauthorized persons will not be able to access them. The results of the project will be presented in research reports. The research reports will only contain summary statistics about the participants, that is, the number of participants, the proportion of women, age distribution, etc.

How do I get information about the project results?

Research reports can be ordered from the responsible researcher (see below). It usually takes-time (more than a year) before there is a complete report. Aggregate information about other participants' decisions can be obtained on request.

Responsible researcher

Lund University is responsible for the project. The researcher responsible is Professor Erik Wengström at the Department of Economics (email: erik.wengstrom@nek.lu.se).

Your participation is voluntary and you can choose to cancel your participation at any time by pressing a button that cancels the questionnaire. If you choose not to participate or want to cancel your participation, you do not need to state why.

[] I have received information on data confidentiality and agree to participate in the study

3.2.2 Vaccine history and attitudes

Before allocating participants into the government condition and the researcher condition, we measured participants COVID-19 vaccination history and vaccination attitudes.

- Have you taken a first dose of a COVID-19 vaccine? (Yes/No)
- Have you taken a second dose of a COVID-19 vaccine? (Yes/No)
- Have you taken a third dose of a COVID-19 vaccine? (Yes/No)
- Have you taken a fourth dose of a COVID-19 vaccine? (Yes/No)
- Have you taken a vaccine against the flu in the last 6 months? (Yes/No)
- Have you donated blood in the last 6 months? (Yes/No)
- How safe do you think the first two doses of the currently approved COVID-19 vaccines are? (Completely safe/Quite safe/Not very safe/Not safe at all)

- In terms of reducing the risk of becoming seriously ill from COVID-19, how effective do you think it would be for you to take an additional shot of a COVID-19 vaccine? (Very effective/Quite effective/Not very effective/Not effective at all)

- To what extent do you agree with the following statements? (Completely disagree, Disagree, Neither agree nor disagree, Agree, Completely agree)

  - When it comes to the COVID-19 vaccine, I trust the Public Health Agency of Sweden

  - When it comes to the COVID-19 vaccines, I trust the researchers who are studying the effects of the vaccines

3.2.3 Information of the incentive program

Please read the following information carefully and answer the questions below.

During the COVID-19 vaccination campaign between May and July 2021, some individuals in Sweden were invited to participate in a COVID-19 incentive program. Participants were paid a financial incentive of 200 Swedish kronor if they proceeded to get vaccinated within 30 days after the vaccine became available to them. **[TREATMENT RESEARCHER: A team of researchers participated in the implementation of the incentive program / TREATMENT GOVERNMENT: The Public Health Agency of Sweden participated in the implementation of the incentive program].**

- Are the following statements true or false?

  - The individuals who participated in the incentive program received 200 kr if they took a first dose of a COVID-19 vaccine

  - (TREATMENT RESEARCHER: A team of researchers / TREATMENT GOVERNMENT: Folkhälsomyndigheten participated in the implementation of the incentive program)

- Have you heard before that some individuals were invited to participate in this COVID-19 incentive program? (Definitively yes, Probably yes, I am not sure, Probably no, Definitively no)

3.2.4 Filler questions (preferences and sociodemographics)

To obfuscate the purpose of the study, thereby avoiding experimenter demand effects, we added a screen of filler questions unrelated to COVID-19.

- In the following, we will ask you several questions about your willingness to act in certain ways. Please indicate your answer on a scale from 0 to 10. A 0 means "completely unwilling to do so," and a 10 means "very willing to do so." You can also use any number between 0 and 10 to indicate where you fall on the scale, using 0, 1, 2, 3, 4, 5, 6, 7, 8, 9, or 10.
  - How willing are you to give to good causes without expecting anything in return?
  - In general, how willing are you to take risks?
  - How willing are you to give up something that is beneficial for you today in order to benefit more from that in the future?
- What year were you born? (Response scale: 1955-2005)
- Do you identify yourself as a woman or a man? (Response scale: woman, man, neither man nor woman)
- What education do you have? (fill in the highest you have.) (Response scale: elementary or lower, high-school, professional training, ongoing university studies, university studies, research studies)
- How much is your total income per month after taxes including public benefits? Calculate also your loan if you are a student. Please answer even if you are not sure. (Response scale: 0-5000kr, 5001-10000kr, 10001-15000kr, 15001-20000kr, 20001-25000kr, 25001-30000kr, 30001-35000kr, 35001-40000kr , 40001-45000kr, 45001-50000kr, 50000kr-55000kr, more than 55000kr)

3.2.5 Outcome measures

- Do you plan to take a COVID-19 vaccine shot (regardless of whether it is your first, second, third, or fourth shot) within the next 6 months? (yes, no)
- Suppose that there would be a new outbreak of the COVID-19 pandemic in 6 months and the Swedish Public Health Agency would recommend people to take an additional COVID-19

vaccine shot (regardless of the number of shots they got in the past). Thinking of such a situation, would you take an additional shot? (yes, no)

- To what extent do you agree with the following statements (5-points Likert scales):
  - In general, COVID-19 vaccines are safe
  - I am worried about the side effects from COVID-19 vaccines
  - In general, COVID-19 vaccines are highly effective at protecting my health
- Suppose that there would be a new outbreak of the COVID-19 pandemic in 6 months and the Swedish Public Health Agency would recommend people to take an additional COVID-19 vaccine shot (regardless of the number of shots they got in the past). In this situation, to what extent do you agree with the following statements? (5-points Likert scales):
  - I would be willing to take the personal costs of getting an additional COVID-19 vaccine shot (e.g., time, discomfort, mild side effects) for the greater good of society
  - I think people would have a civic duty or a moral obligation to get an additional COVID-19 vaccine shot
  - Not taking the COVID-19 vaccine shot would be generally viewed as socially inappropriate in this situation

3.3 US study

3.3.1 Consent form

Welcome

This study on decision-making is conducted by researchers at the University of Zurich, Switzerland.

The study takes about 4 minutes to complete. You will receive a fixed payment of $1.00 (=£0.82) for completing the study.

Your participation in this study does not pose any risks to your health or well-being. All the information gathered in this study is kept strictly anonymous and will never be associated with your identity.

By continuing you are acknowledging that you understand the above information and give your consent to participate in the study. Participation in this study is voluntary. You may withdraw

your consent and discontinue participation in the study at any time. Doing so will not result in any penalty.

Do you want to participate in this study?

- I want to participate
- I do NOT want to participate

3.3.2 Sociodemographics, beliefs about existence of state incentive program and vaccination attitudes

- What was your state of residence in 2021?
- What is your age?
- Do you identify yourself as a woman or a man? (Woman, Man, Neither man nor woman)
- How would you describe your ethnicity/race? (European American / White, African American / Black, Hispanic / Latino, Asian / Asian American, Other)
- Which category best describes your highest level of education? (Eighth Grade or less, Some High School, High School degree / GED, Some College, 2-year College Degree, 4-year College Degree, Master's Degree, Doctoral Degree, Professional Degree (JD, MD, MBA))
- What is your current employment status? (Full-time employee, Part-time employee, Self-employed or small business owner, Unemployed and looking for work, Student, Not in labor force (for example: retired, or full-time parent))
- What was your TOTAL household income, before taxes, last year (2021)? ($0 - $9,999, $10,000 - $14,999, $15,000 - $19,999, $20,000 - $29,999, $30,000 - $39,999, $40,000 - $49,999, $50,000 - $74,999, $75,000 - $99,999, $100,000 - $124,999, $125,000 - $149,999, $150,000 - $199,999, $200,000+)
- In politics, as of today, do you consider yourself a Republican, a Democrat or an independent? (Republican, Democrat, Independent)
- Who did you support in the presidential election in 2020? If you did not vote, just choose the person you wanted to win the election at that time. (Joe Biden, Donald Trump, Other)

- (If Independent) As of today, do you lean more to the Democratic Party or the Republican Party? (Democratic Party, Republican Party)
- Last year (2021), did any governmental organization in [STATE OF RESIDENCE 2021] (for example, the state government) offer any financial compensation (for example, a cash payment or participation in a lottery) to people who got vaccinated against COVID-19?
- Last year (2021), did any governmental organization in [STATE OF RESIDENCE 2021] (for example, the state government) offer any financial compensation (for example, a cash payment or participation in a lottery) to people who got vaccinated against the flu?
- Last year (2021), did any governmental organization in [STATE OF RESIDENCE 2021] (for example, the state government) offer any financial compensation (for example, a cash payment or participation in a lottery) to people who donated blood?
- Last year (2021), did any governmental organization in [STATE OF RESIDENCE 2021] (for example, the state government) offer any financial compensation (for example, a cash payment or participation in a lottery) to people who quit smoking?
- How many COVID-19 vaccine shots have you taken? (None, 1 shot, 2 shots, 3 shots, 4 or more shots)
- (If $>$ 0 shots) In which month and year did you get your first dose of a COVID-19 vaccine? If you don't remember, please answer with your best guess
- Have you ever tested positive for COVID-19? (Yes, No, I don't know)
- How safe do you think the first two doses of the currently approved COVID-19 vaccines are? (Completely safe, Quite safe, Not very safe, Not safe at all)
- In terms of reducing the risk of becoming seriously ill from COVID-19, how effective do you think the first two doses of the currently approved COVID-19 vaccines are? (Very effective, Quite effective, Not very effective, Not effective at all)
- How willing would you be to take another COVID-19 vaccine shot within the next year if it was recommended that you do so? (Very willing, Willing, Neither willing nor unwilling, Unwilling, Very unwilling)

- In terms of reducing the risk of becoming seriously ill from COVID-19, how effective do you think it would be for you to take an additional shot of a COVID-19 vaccine? (Very effective, Quite effective, Not very effective, Not effective at all)

- To what extent do you agree with the following statements? (Completely disagree, Disagree, Neither agree nor disagree, Agree, Completely agree)

  - Diseases like autism, multiple sclerosis, and diabetes might be triggered through vaccination.

  - When it comes to the COVID-19 vaccines, I trust pharmaceutical companies that develop the vaccines

  - When it comes to the COVID-19 vaccines, I trust the researchers who are studying the effects of the vaccines

  - When it comes to the COVID-19 vaccine, I trust the US Center for Disease Control

3.3.3 Incentives condition

The participants in the information condition received detailed information about their state's (or counties') COVID-19 vaccine incentive program. We provide the descriptions of the incentive programs that we gave to participants below. Participants also had to answer the following two comprehension questions:

- Which governmental organization implemented the [NAME OF THE INCENTIVE PROGRAM] program? (3 options)

- When did the [NAME OF THE INCENTIVE PROGRAM] program end? (3 options)

They could only continue with the study if they answered both comprehension questions correctly. We then also asked participants whether they were aware of the program:

- Had you heard before about the "Vax for the Win" program? (Definitively yes, Probably yes, I am not sure, Probably no, Definitively no)

Here are the descriptions of the incentive programs that were shown to participants:

(STATE OF RESIDENCE 2021 = California) **California's "Vax for the Win" program**

The “Vax for the Win” Program was a Californian $116.5 million COVID-19 vaccine incentive program implemented by the government of California which took place between May 2021 and January 2022. The purpose of this program was to increase COVID-19 vaccination rates.

- All Californians aged 12 and over who were at least partially vaccinated against COVID were automatically eligible for the cash prize drawings. In total $16.5 million in cash prizes were paid out.
- Moreover, two million people who completed their COVID-19 vaccination were automatically eligible to receive a $50 present or grocery card, worth a total of $100 million.

(STATE OF RESIDENCE 2021 = Florida) **Alachua County and Flagler County vaccine incentive programs**

The Alachua County vaccine incentive program and the Flagler County vaccine incentive program were Florida COVID-19 vaccine incentive program implemented by Florida counties. The purpose of these programs was to increase COVID-19 vaccination rates.

- Alachua County vaccine incentive program: Residents of Alachua County who had received a dose of a COVID-19 vaccine (including booster shots) in September 2021 received $25 Visa gift cards.
- Flagler County vaccine incentive program: Residents of Flagler County were paid a $10 food coupon as incentive to receive a dose of a COVID-19 by July 2021. The incentive was only offered at specific locations and days of the week.

(STATE OF RESIDENCE 2021 = Illinois) **Illinois “All in for the win” program**

The “All in for the win” program was an Illinois $10 million COVID-19 vaccine incentive program implemented by the government of Illinois which took place between July 2021 and August 2021. The purpose of this program was to increase COVID-19 vaccination rates.

- Illinois residents aged 18 and over who had received at least one dose of a COVID-19 vaccine automatically participated in three drawings to win a $1 million cash prize and forty $100k cash prizes.
- Youth between the ages of 12 and 17 who had received at least one dose of a COVID-19 vaccine automatically participated in twenty drawings to win $150,000 scholarships.

(STATE OF RESIDENCE 2021 = Kentucky) **Kentucky's "Shot At A Million" program**

The "Shot At A Million" program was a Kentucky COVID-19 vaccine incentive program implemented by the government of Kentucky which took place between June 2021 and August 2021. The purpose of this program was to increase COVID-19 vaccination rates.

- Kentuckians aged 18 and over who were at least partially vaccinated against COVID were eligible for cash prize drawings. Three individuals each received a cash prize of $1 million.
- Youth between the ages of 12 and 17 who were at least partially vaccinated against COVID participated in fifteen drawings of a full scholarships (including room-and-board and tuition) to any Kentucky public college, university, technical or trade school.

(STATE OF RESIDENCE 2021 = Louisiana) **Louisiana's "Shot For $100" program**

The "Shot For $100" program was a Louisiana COVID-19 vaccine incentive program implemented by the Louisiana Department of Health which took place between October 2021 and December 2021. The purpose of this program was to increase COVID-19 vaccination rates.

- Anyone who took a first dose of a COVID-19 vaccine was eligible to receive a $100 incentive card. Minors were eligible for the cash incentive but required parental consent to get the vaccine.

(STATE OF RESIDENCE 2021 = Michigan) **Michigan's "Shot To Win Sweepstakes" program**

The "Shot To Win Sweepstakes" program was a Michigan $5.5 million COVID-19 vaccine incentive program implemented by the government of Michigan which ended in July 2021. The purpose of this program was to increase COVID-19 vaccination rates.

- Michigan residents aged 18 and over who were at least partially vaccinated against COVID were eligible for cash prize drawings. In total $5 million in cash prizes were paid out, including 30 daily prizes of $50,000, a $1 million prize and a $2 million grand prize.
- Youth between the ages of 12 and 17 who were at least partially vaccinated against COVID automatically participated in nine drawings to win $55,000 scholarships.

(STATE OF RESIDENCE 2021 = Missouri) **Missouri's "MO VIP" program**

The "MO VIP" program was a Missourian $9 million COVID-19 vaccine incentive program implemented by the government of Missouri which took place between July 2021 and October 2021. The purpose of this program was to increase COVID-19 vaccination rates.

- Missourian residents aged 18 and over who were at least partially vaccinated against COVID were eligible for cash prize drawings. 800 individuals each received a cash prize of $10,000.
- Youth between the ages of 12 and 17 who were at least partially vaccinated against COVID participated in 100 drawings to win $10,000 scholarships.

(STATE OF RESIDENCE 2021 = New York) **New York's "Vax Scratch" program**

The "Vax Scratch" Program was a New York million COVID-19 vaccine incentive program implemented by the government of New York which took place between May 2021 and June 2021. The purpose of this program was to increase COVID-19 vaccination rates.

- New Yorkers aged 18 and over who took a first dose of a COVID-19 vaccine at select state-run vaccination sites received a lottery scratch ticket with prizes from $20 up to $5 million. The lottery scratch tickets, which are also sold by retailers across the state, were worth $20.

(STATE OF RESIDENCE 2021 = North Carolina) **North Carolina's "Summer Cash Drawing" program**

The "Summer Cash Drawing" program was a North Carolinian $4.5 million COVID-19 vaccine incentive program implemented by the government of North Carolina which took place between June 2021 and August 2021. The purpose of this program was to increase COVID-19 vaccination rates.

- North Carolinians aged 18 and over who had received at least one dose of a COVID-19 vaccine automatically participated in four drawings for a chance to win a $1 million cash prize.
- Youth between the ages of 12 and 17 who had received at least one dose of a COVID-19 vaccine automatically participated in four drawings to win $125,000 to pay for their post-secondary education.

(STATE OF RESIDENCE 2021 = Ohio) **Ohio's "Vax-A-Million" program**

The "Vax-A-Million" program was an Ohioan $5 million COVID-19 vaccine incentive program implemented by the government of Ohio which took place between May 2021 and June 2021. The purpose of this program was to increase COVID-19 vaccination rates.

- Ohioans aged 18 and older who had received at least one dose of a COVID-19 vaccine could enter a lottery to win one of five $1 million prizes.
- Ohioans ages 12-17 who had received at least one dose of a COVID-19 vaccine could enter a lottery to win one of five scholarships which would pay for their college degree at any Ohio state college or university.

(STATE OF RESIDENCE 2021 = Pennsylvania) **Philadelphia's vaccine incentive program**

The Philadelphia vaccine incentive program was a Pennsylvania COVID-19 vaccine incentive program implemented by the Philadelphia Health Department which ended in March 2022. The purpose of this program was to increase COVID-19 vaccination rates.

- Philadelphia residents who completed their COVID vaccine series at a participating Health Department vaccine clinic received a payment of $100. In total, $20,000 in incentives were paid out.

(STATE OF RESIDENCE 2021 = Texas) **Houston's vaccine incentive program**

The Houston vaccine incentive program was a Texan COVID-19 vaccine incentive program implemented by the Houston Health Department which ended in March 2022. The purpose of this program was to increase COVID-19 vaccination rates.

- The program provided a total of fourteen $1,000 weekly prizes to people who had gotten their first dose or booster shot. People of all ages eligible for vaccination were allowed to participate in the incentive program.

3.3.4 Outcome measures

- Do you plan to take a COVID-19 vaccine shot (regardless of whether it is your first, second, third, or fourth shot) within the next 6 months? (yes, no)

- Suppose that there would be a new outbreak of the COVID-19 pandemic in 6 months and the Center for Disease Control would recommend people to take an additional COVID-19 vaccine shot (regardless of the number of shots they got in the past). Thinking of such a situation, would you take an additional shot? (yes, no)
- To what extent do you agree with the following statements (5-points Likert scales):
  - In general, COVID-19 vaccines are safe
  - I am worried about the side effects from COVID-19 vaccines
  - In general, COVID-19 vaccines are highly effective at protecting my health
- Suppose that there would be a new outbreak of the COVID-19 pandemic in 6 months and the Center for Disease Control would recommend people to take an additional COVID-19 vaccine shot (regardless of the number of shots they got in the past). In this situation, to what extent do you agree with the following statements? (5-points Likert scales):
  - I would be willing to take the personal costs of getting an additional COVID-19 vaccine shot (e.g., time, discomfort, mild side effects) for the greater good of society
  - I think people would have a civic duty or a moral obligation to get an additional COVID-19 vaccine shot
  - Not taking the COVID-19 vaccine shot would be generally viewed as socially inappropriate in this situation
- Do you plan to take a flu vaccine next winter (2022-2023)?
- Do you plan to donate blood in the next 6 months?
- How much trust and confidence do you have in the government of the state where you live when it comes to handling state problems – a great deal, a fair amount, not very much or none at all?
- Suppose that there would be a new outbreak of the COVID-19 pandemic in 6 months, the Center for Disease Control would recommend people to take an additional COVID-19 vaccine shot (regardless of the number of shots they got in the past) and that every person getting an additional shot would receive $20. Thinking of such a situation, would you take an additional shot? (yes, no)

3.3.5 Awareness incentive programs

- In 2021, did any governmental organization in your state offer any monetary compensation (for example, participation in a vax lottery or payments) to people who got a COVID-19 shot? (Definitively yes, Probably yes, I am not sure, Probably no, Definitively no)
- (Description of the state incentive program in the residence country of the participant) Had you heard about the [Name of the state vaccine incentive program] before filling out today's survey? (Definitively yes, Probably yes, I am not sure, Probably no, Definitively no)

4 References

72. Mathieu, E. et al. (2021) “A global database of COVID-19 vaccinations,” Nature Human Behaviour, 5(7): 947-953.

73. Eurostat (2022) “Gini coefficient of equivalised disposable income - EU-SILC survey,” URL: https://appsso.eurostat.ec.europa.eu/nui/show.do?dataset=ilc_di12.

74. Falk, A., Becker, A., Dohmen, T., Enke, B., Huffman, D. and Sunde, U. (2018) “Global Evidence on Economic Preferences,” Quarterly Journal of Economics, 133(4): 1645-1692.

75. WGI (2018) “CAF world giving index 2018.”

76. Altay, S., Schwartz, M., Hacquin, A. S., Allard, A., 72 Blancke, S., and Mercier, H. (2022) “Scaling up interactive argumentation by providing counterarguments with a chatbot,” Nature Human Behaviour, 1-14.
