# Supplementary material for: Financial incentives for vaccination do not have negative unintended consequences
Source: Nature. 2023 Jan 11;613(7944):526–33. doi: 10.1038/s41586-022-05512-4 (PMC9833033; doi:10.1038/s41586-022-05512-4)
Supplement: Supplementary file 2 — Reporting Summary [file 41586_2022_5512_MOESM2_ESM.pdf]

nature portfolio

Corresponding author(s): Florian H. Schneider, Pol Campos-Mercade, Armando N. Meier

Last updated by author(s): 2022/10/18

Reporting Summary

Nature Portfolio wishes to improve the reproducibility of the work that we publish. This form provides structure for consistency and transparency in reporting. For further information on Nature Portfolio policies, see our Editorial Policies and the Editorial Policy Checklist.

Statistics

For all statistical analyses, confirm that the following items are present in the figure legend, table legend, main text, or Methods section.

n/a	Confirmed	
☐	☒	The exact sample size (n) for each experimental group/condition, given as a discrete number and unit of measurement
☐	☒	A statement on whether measurements were taken from distinct samples or whether the same sample was measured repeatedly
☐	☒	The statistical test(s) used AND whether they are one- or two-sided<br>*Only common tests should be described solely by name; describe more complex techniques in the Methods section.*
☐	☒	A description of all covariates tested
☐	☒	A description of any assumptions or corrections, such as tests of normality and adjustment for multiple comparisons
☐	☒	A full description of the statistical parameters including central tendency (e.g. means) or other basic estimates (e.g. regression coefficient) AND variation (e.g. standard deviation) or associated estimates of uncertainty (e.g. confidence intervals)
☐	☒	For null hypothesis testing, the test statistic (e.g. F, t, r) with confidence intervals, effect sizes, degrees of freedom and P value noted<br>*Give P values as exact values whenever suitable.*
☒	☐	For Bayesian analysis, information on the choice of priors and Markov chain Monte Carlo settings
☒	☐	For hierarchical and complex designs, identification of the appropriate level for tests and full reporting of outcomes
☐	☒	Estimates of effect sizes (e.g. Cohen's d, Pearson's r), indicating how they were calculated

Our web collection on statistics for biologists contains articles on many of the points above.

Software and code

Policy information about availability of computer code

Data collection	Administrative vaccination data and survey data. The surveys were programmed in Qualtrics (instructions are provided in SI section 3).
Data analysis	Stata 16, code and data are available on the referenced Zenodo repository: https://doi.org/10.5281/zenodo.7214856.

For manuscripts utilizing custom algorithms or software that are central to the research but not yet described in published literature, software must be made available to editors and reviewers. We strongly encourage code deposition in a community repository (e.g. GitHub). See the Nature Portfolio guidelines for submitting code & software for further information.

Data

Policy information about availability of data

All manuscripts must include a data availability statement. This statement should provide the following information, where applicable:

- Accession codes, unique identifiers, or web links for publicly available datasets
- A description of any restrictions on data availability
- For clinical datasets or third party data, please ensure that the statement adheres to our policy

Data to reproduce the analyses is available on the referenced Zenodo repository: https://doi.org/10.5281/zenodo.7214856.

Field-specific reporting

Please select the one below that is the best fit for your research. If you are not sure, read the appropriate sections before making your selection.

☐ Life sciences ☒ Behavioural & social sciences ☐ Ecological, evolutionary & environmental sciences

For a reference copy of the document with all sections, see nature.com/documents/nr-reporting-summary-flat.pdf

Behavioural & social sciences study design

All studies must disclose on these points even when the disclosure is negative.

Study description

We implement three quantitative experimental studies, two in Sweden and one in the US.

Details Swedish main study: We use a combination of random exposure to financial incentives, population wide administrative vaccination records, and rich survey data from two surveys. To causally measure the unintended consequences of offering financial incentives, we exploit a randomized controlled trial (RCT) in the context of financial incentives for COVID-19 vaccination. In this previous RCT, participants were randomly allocated to either a financial incentives condition that offered monetary payments conditional on getting a first dose of a COVID-19 vaccine or a control condition that did not offer any financial incentives. The setting allows us to compare individuals who were randomly offered financial incentives for vaccination with individuals who were not offered any financial incentives. To measure the unintended consequences, we then combine the RCT data with novel Swedish administrative records for second dose uptake and with rich individual-level survey data on behaviors, morals, perceptions, and feelings from two surveys.

Details Swedish complementary study: We examine the question whether the impact of incentives differs when paid by the government rather than by researchers in a complementary study. We use the fact that the previous RCT was implemented in a collaboration between researchers and the Public Health Agency of Sweden. Some study participants were told that “a team of researchers participated in the implementation of the incentive program” while others were told that the Public Health Agency did so. We study whether people’s reactions to incentives depend on whether the government or researchers offered the payments.

Details US study: We complement our evidence from Sweden with evidence on the effects of large-scale incentive programs implemented by US state governments. Participants randomly assigned to the incentives condition received detailed information about their state’s COVID-19 vaccine incentive program, while participants in the control condition did not receive this information. Since a majority of the participants were unaware that their state offered incentives for vaccination, this experimental design creates random variation in perceived exposure to incentives. We analyzed whether the provision of information had any impact on participants’ willingness to get future shots of a COVID-19 vaccine, morals, and safety perceptions. To avoid experimenter demand effects, we measured these outcomes in an apparently unrelated follow-up survey about five days after the initial survey where we provided the information (in SI section 2.6.3, we show that participants retained the information from the initial survey).

Research sample

Swedish main study: Representative sample of participants in terms of age and region of the Swedish population aged 18-49 (N=5,020, 42% male, average age=34.62, s.d.=8.27). We collected survey data with the aid of survey company Norstat which allowed us to sample a general population sample representative of age and region of individuals in the desired age range. Accordingly, we invited participants from the initial RCT again because it provides us with random assignment of participants to financial incentives for taking a first dose.

Swedish complementary study: Representative sample of participants in terms of age and region of the Swedish population aged 18-49 (N=1,001, 46% male, average age=31.56, s.d.=8.47). We collected survey data with the aid of survey company Norstat. We chose this sample to have a comparable sample to the Swedish main study.

US study: Representative sample of participants in terms of gender of the US population (N=3,062, 50% male, 41% Democrats, 22% unvaccinated, average age=36.76, s.d.=13.54). We targeted a broad sample including vaccine positive and vaccine hesitant individuals, as well as a substantial share of Republicans and independents. We collected survey data with the aid of survey company Prolific. We chose Prolific because they allowed us to recruit substantial share of Republicans and independents and vaccine positive and vaccine hesitant individuals.

Sampling strategy

Swedish main study: A random sampling procedure was used to allocate participants to the financial incentives and the control condition in the initial RCT. We pre-registered the data collection (and analysis) at the AEA RCT Registry (https://www.socialscienceregistry.org/trials/8727 and www.socialscienceregistry.org/trials/9580). We aimed to have enough power to detect even small impacts of offering financial incentives on behaviors, morals, perceptions, and feelings. Hence, for both surveys, we asked the survey company to recruit as many participants as possible who participated in the RCT. We pre-registered that, according to our power calculations, this would give us 80% power to detect an effect size (= Cohen’s d) of about 0.12 standard deviations.

Swedish complementary study: A random sampling procedure was used to allocate participants to the researcher and the government condition. We pre-registered the data collection (and analysis) at the AEA RCT Registry (www.socialscienceregistry.org/trials/9583 and www.socialscienceregistry.org/trials/9584). We pre-registered that, according to our power calculations, this would give us 80% power to detect an effect size (= Cohen’s d) of about 0.2 standard deviations.

US study: A random sampling procedure was used to allocate participants to the incentives and the control condition. We pre-registered the data collection (and analysis) at the AEA RCT Registry (hwww.socialscienceregistry.org/trials/9607). We pre-registered that, according to our power calculations, this would give us 80% power to detect an effect size (= Cohen’s d) of about 0.2 standard

deviations.

Data collection

Swedish study: Population-wide vaccination records were collected and linked by the Public Health Agency of Sweden. The survey data were collected via online surveys that participants reached through email links. The sample was collected by the survey company Norstat based on an existing, actively recruited panel. The survey company was blind to the experimental conditions and the study's hypotheses.

Swedish complementary study: The data were collected via online surveys that participants reached through email links. The sample was collected by the survey company Norstat based on an existing, actively recruited panel and exluded participants who participated in the previous RCT. The survey company was blind to the experimental conditions and the study's hypotheses.

US study: The data were collected via online surveys that participants reached through an online platform. The sample was collected by the survey company Prolific based on an existing panel. The survey company was blind to the experimental conditions and the study's hypotheses.

Timing

Swedish main study, first survey: The data for the first study were collected between 2022-01-11 and 2022-01-18.
Swedish main study, second survey: The data were collected between 2022-06-16 and 2022-07-05.
Swedish complementary study: The data were collected between 2022-06-16 and 2022-06-28.
US study, first survey: The data were collected between 2022-06-22 and 2022-07-02.
US study, second survey: The data were collected between 2022-06-24 and 2022-07-07.

Data exclusions

Swedish main study: We excluded 19 (27) participants who did not finish the first (second) survey, which is less than 1% of survey participants. Inclusion of those participants does not affect the results and non-completion rates did not differ across conditions (see SI sections 2.1.2 and 2.3.3).
Swedish complementary study: We excluded 39 participants who did not finish the survey (see SI section 2.5.9). Inclusion of those participants does not affect the results and non-completion rates did not differ across conditions.
US study: In total, 3,980 people responded the first survey and 3,062 people responded to the follow-up survey. We can therefore match the two surveys for 3,062 participants. Since the outcome variables are collected in the follow-up survey (see Methods section), we perform the data analysis with the 3,062 participants who answered the follow-up survey (and hence exclude the 918 participants who did not respond to it).

Non-participation

Swedish main study: We have Swedish administrative records for second dose uptake for all participants of the RCT, and hence use data for each participant. Regarding survey measures, we contacted each participant to fill out the surveys and 64% of them completed the first survey and 54% completed the second survey. In both surveys, survey participation was balanced across both conditions, with no differential attrition based on personality characteristics, vaccination status, vaccine hesitancy, or sociodemographics (see SI section 2.1).
Swedish complementary study: 96.30% of all participants completed the survey. Drop out rates do not differ across the treatment conditions (see SI section 2.5.9).
US study: 77% of participants that responded to the first survey also responded to the follow-up survey. Participation in the follow-up survey was balanced across both conditions, with no differential attrition based on vaccination status, vaccine hesitancy, or sociodemographics (see SI section 2.6.1).

Randomization

Swedish main study: Participants were randomly allocated to a financial incentives condition and a control condition.
Swedish complementary study: Participants were randomly allocated to a researcher condition and a government condition.
US study: Participants were randomly allocated to an incentives condition and a control condition.

Reporting for specific materials, systems and methods

We require information from authors about some types of materials, experimental systems and methods used in many studies. Here, indicate whether each material, system or method listed is relevant to your study. If you are not sure if a list item applies to your research, read the appropriate section before selecting a response.

Materials & experimental systems

n/a	Involved in the study	
☒	☐	Antibodies
☒	☐	Eukaryotic cell lines
☒	☐	Palaeontology and archaeology
☒	☐	Animals and other organisms
☐	☒	Human research participants
☒	☐	Clinical data
☒	☐	Dual use research of concern

Methods

n/a	Involved in the study	
☒	☐	ChIP-seq
☒	☐	Flow cytometry
☒	☐	MRI-based neuroimaging

Human research participants

Policy information about studies involving human research participants

Population characteristics

Swedish main study: Representative sample in terms of age, region, and gender of the Swedish population aged 18-49. We provide detailed information on sample characteristics in SI section 2.1.3.
Swedish complementary study: Representative sample in terms of age, region, and gender of the Swedish population aged 18-49. We provide detailed information on sample characteristics in SI section 2.5.3.

	US study: Representative sample of participants in terms of gender of the US population. We targeted a broad sample including vaccine positive and vaccine hesitant individuals, as well as a substantial share of Republicans and independents. We provide detailed information on sample characteristics in SI section 2.6.2.
Recruitment	Swedish main study: The sample was collected by the survey company Norstat based on an existing, actively recruited panel and based on participants in the initial RCT.<br>Swedish complementary study: The sample was also collected by the survey company Norstat based on an existing, actively recruited panel (who did not take part in the initial RCT).<br>US study: The sample was collected by the survey company Prolific based on an online panel. Panel participants are primarily recruited to Prolific via word of mouth, including word of mouth via social media. Regarding self-selection bias, due to informed consent procedures, people may have chosen to participate based on their knowledge of or interest in our survey topic. This is true for any survey study that involves participant consent. Because participants were randomly assigned to condition after the decision to participate, it is unlikely self-selection would result in the effects observed in our experiments. Moreover, we provide empirical evidence that bias from self-selection is unlikely (see SI sections 1.1.6 and 2.5.9).
Ethics oversight	Swedish main study: The Swedish ethical review authority (Etikprövningsmyndigheten) approved the protocols of the study (reference number 2021-06367-02). Informed consent was obtained from all study participants as part of the survey.<br><br>Swedish complementary study: The Human Subjects Committee of the Faculty of Economics, Business Administration, and Information Technology at University of Zurich approved the protocols of the study (reference numbers 2022-045). Informed consent was obtained from all study participants as part of the survey.<br><br>US study: The Human Subjects Committee of the Faculty of Economics, Business Administration, and Information Technology at University of Zurich approved the protocols of the study (reference numbers 2022-045). Informed consent was obtained from all study participants as part of the survey.

Note that full information on the approval of the study protocol must also be provided in the manuscript.
